# Supplementary material for: Multi‐omic network analysis identified betacellulin as a novel target of omega‐3 fatty acid attenuation of western diet‐induced nonalcoholic steatohepatitis
Source: EMBO Mol Med. 2023 Oct 20;15(11):e18367. doi: 10.15252/emmm.202318367 (PMC10630881; doi:10.15252/emmm.202318367)
Supplement: Supplementary file 1 — Appendix S1 [file EMMM-15-e18367-s009.pdf]

Table of Contents **Page number**

Appendix Figure S1	2
Appendix Figure S2	4
Appendix Table S1	7

Appendix Figure S1

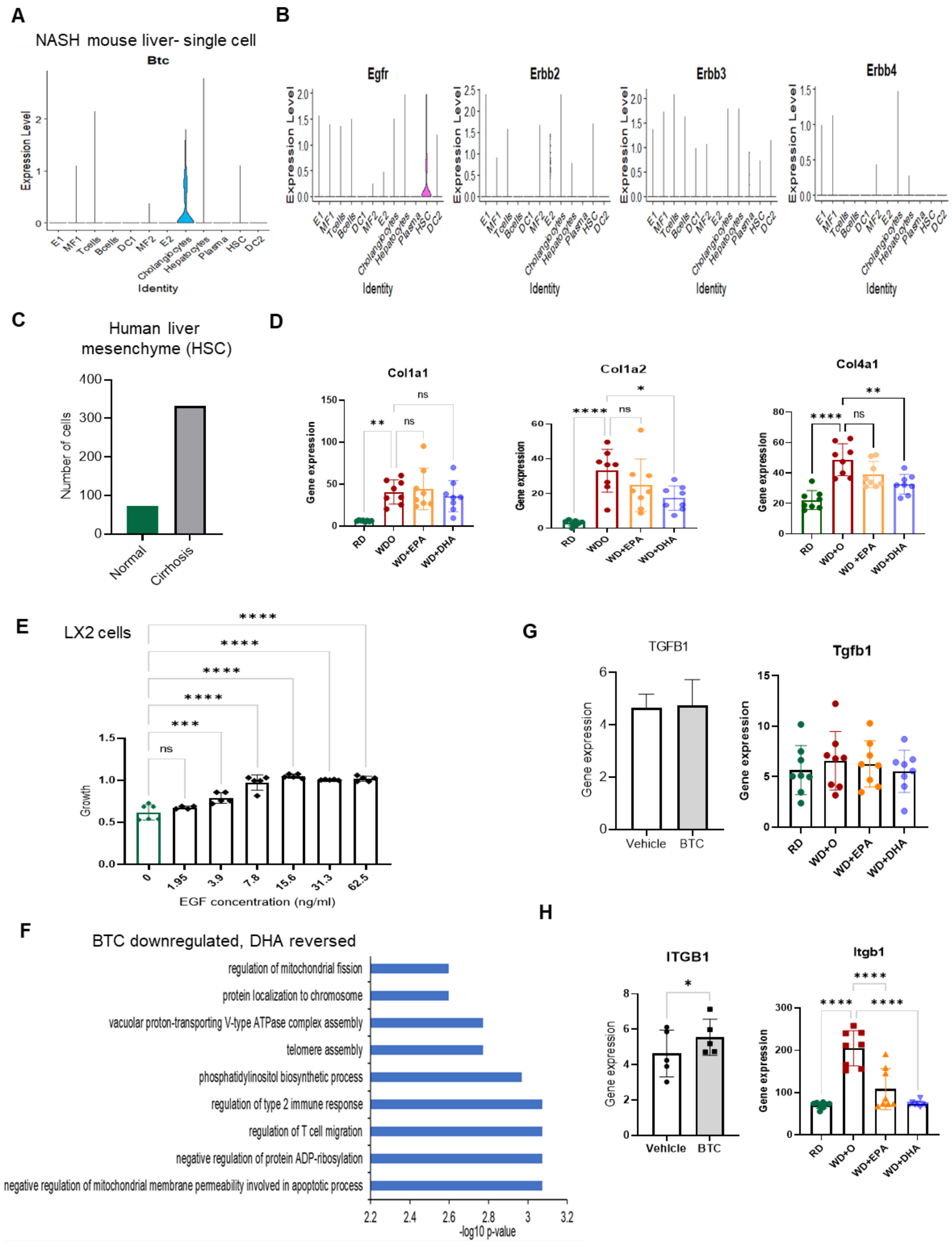

Appendix Figure S1. A. The expression of Btc in Mouse NASH liver single cell RNA sequence data shown here with the maximum expression in liver cholangiocytes (MF1-KC; MF2 -NAM).

B. The expression of Egfr and other Erbbs in the NASH mouse model single cell RNA sequence data.
C. The number of Mesenchymal cells (Hepatic stellate cells) in Human liver samples with enrichment in Cirrhosis with more than 3-fold increase in numbers than normal liver samples.
D. The different collagen genes expression in the NASH preventive model shown is in bar graphs colored by treatment effects (Data are mean +/- SD, N=8 mice/treatment group. Ordinary One-way ANOVA, with multiple comparisons test with WD+O, ns (not significant), *p<0.05, ** p<0.001, ****p<0.0001).
E. The growth of LX2 cells in response to EGF in a dose dependent manner shown in the bar graph. (Ordinary One-way ANOVA, with multiple comparisons test with Control, ns (not significant), *** p<0.005, ****p<0.0001)
F. The gene enrichment analysis shown in a bar plot, regulation of mitochondrial fission and mitochondrial membrane permeability mediated apoptotic pathway are significantly down regulated by BTC treatment in LX2 cells while they are reversed by DHA treatment in the *in vivo* mouse model.
G. TGFB1 expression in LX2 cells treated with BTC (grey) (20 ng/ml; N=5 experiments, paired, one-sided t-test, ns (not significant) and in the NASH preventive model is shown in bar graphs colored by treatment effects (Ordinary One-way ANOVA, with multiple comparisons test with WD+O, ns (not significant).
H. ITGB1 expression in LX2 cells treated with BTC (grey) (20 ng/ml; N=5 experiments, paired, one-sided t-test, *p<0.05) and in the NASH preventive model is shown in bar graphs colored by treatment effects (Data are mean +/- SD, N=8 mice/treatment group. Ordinary One-way ANOVA, with multiple comparisons test with WD+O, ns (not significant), *p<0.05, ** p<0.001, *** p<0.005, ****p<0.0001).

Appendix Figure S2

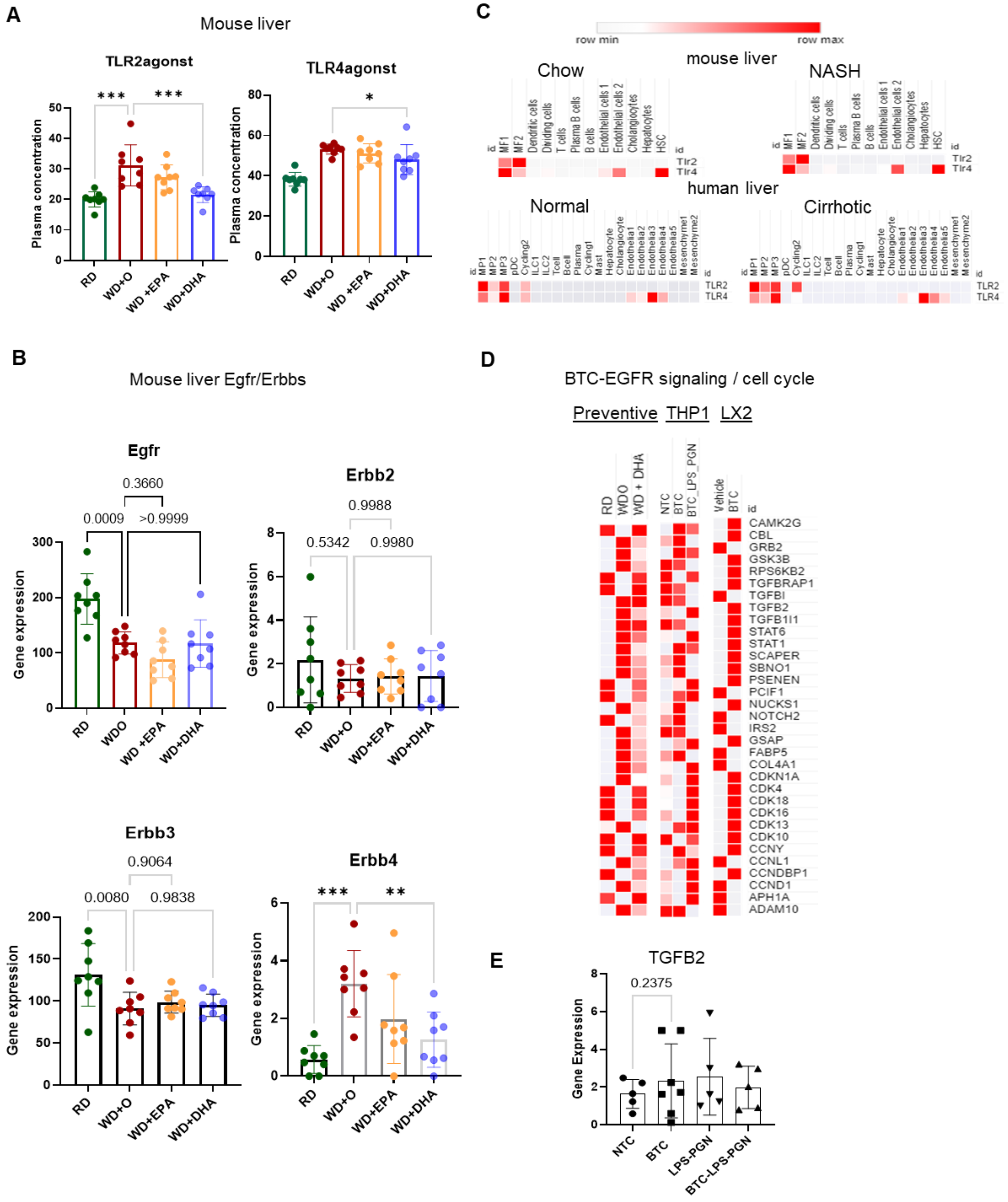

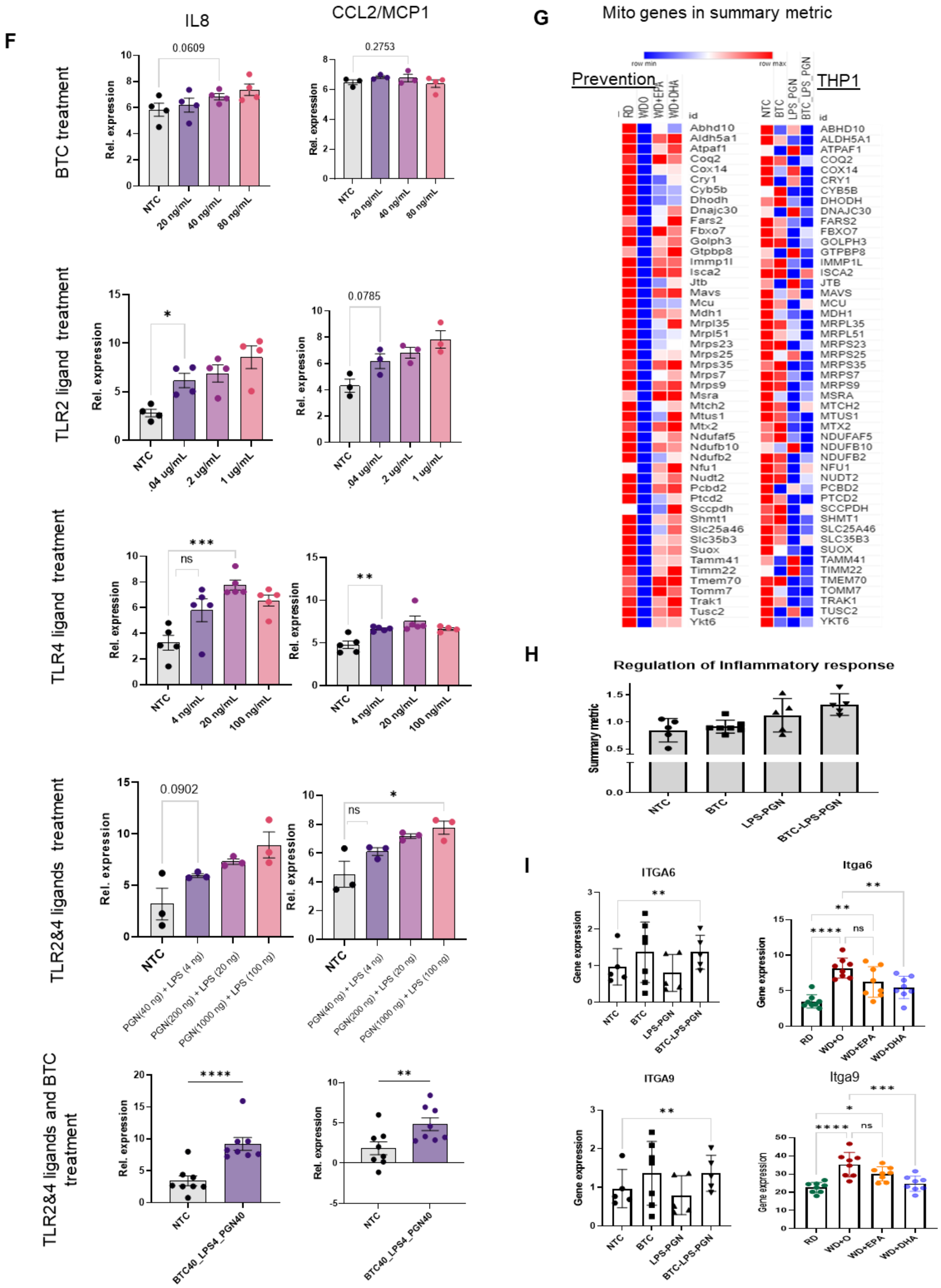
F
G
H
I
IL8
CCL2/MCP1
BTC treatment
Rel. expression
0.0609
0.2753
NTC
20 ng/mL
40 ng/mL
80 ng/mL
TLR2 ligand treatment
*
0.0785
.04 ug/mL
.2 ug/mL
1 ug/mL
TLR4 ligand treatment
***
ns
**
4 ng/mL
20 ng/mL
100 ng/mL
TLR2&4 ligands treatment
0.0902
PGN(40 ng) + LPS (4 ng)
PGN(200 ng) + LPS (20 ng)
PGN(1000 ng) + LPS (100 ng)
TLR2&4 ligands and BTC treatment
****
BTC40_LPS4_PGN40
Mito genes in summary metric
row min
row max
Prevention
THP1
RD
WDO
WD+EPA
WD+DHA
NTC
BTC
LPS_PGN
BTC_LPS_PGN
id
Abhd10
Aldh5a1
Atpaf1
Coq2
Cox14
Cry1
Cyb5b
Dhodh
Dnajc30
Fars2
Fbxo7
Golph3
Gtpbp8
Immp1l
Isca2
Jtb
Mavs
Mcu
Mdh1
Mrpl35
Mrpl51
Mrps23
Mrps25
Mrps35
Mrps7
Mrps9
Msra
Mtch2
Mtus1
Mtx2
Ndufaf5
Ndufb10
Ndufb2
Nfu1
Nudt2
Pcbd2
Ptcd2
Sccpdh
Shmt1
Slc25a46
Slc35b3
Suox
Tamm41
Timm22
Tmem70
Tomm7
Trak1
Tusc2
Ykt6
ABHD10
ALDH5A1
ATPAF1
COQ2
COX14
CRY1
CYB5B
DHODH
DNAJC30
FARS2
FBXO7
GOLPH3
GTPBP8
IMMP1L
ISCA2
JTB
MAVS
MCU
MDH1
MRPL35
MRPL51
MRPS23
MRPS25
MRPS35
MRPS7
MRPS9
MSRA
MTCH2
MTUS1
MTX2
NDUFAF5
NDUFB10
NDUFB2
NFU1
NUDT2
PCBD2
PTCD2
SCCPDH
SHMT1
SLC25A46
SLC35B3
SUOX
TAMM41
TIMM22
TMEM70
TOMM7
TRAK1
TUSC2
YKT6
Regulation of Inflammatory response
Summary metric
LPS-PGN
BTC-LPS-PGN
ITGA6
Itga6
Gene expression
WD+O
WD+EPA
WD+DHA
ITGA9
Itga9

Appendix Figure S2. A. Expression of TLR2/4 agonists in the NASH preventive model is shown in bar graphs colored by treatment effects (Data are mean +/- SD, N=8 mice/treatment group). (Ordinary One-way ANOVA, with multiple comparisons test with WD+O, *p<0.05, *** p<0.005)

B. The Egfr/Erbbs expression in the NASH preventive model is shown in bar graphs colored by treatment effects (Data are mean +/- SD, N=8 mice/treatment group). (Ordinary One-way ANOVA, with multiple comparisons test with WD+O, ns (not significant), ** p<0.001, *** p<0.005)
C. The mouse and human liver (with or without NASH/Cirrhosis) cluster wise average TLR2/4 gene expression from the single cell RNA sequence data. The color scale is indicated from high expression of the genes in red to low in white.
D. The gene expression heatmap from BTC/TLR2/4 ligands treated THP-1 and LX2 cells shows genes involved in EGFR pathway and cell cycle pathway that are induced. These set of genes were revered by DHA in the mouse NASH preventive model. The color scale in heatmap is indicated from high expression of the genes in red to low in white.
E. Normalized TGFB2 expression in THP-1 cells treated with BTC and or TLR2/4 ligands (5 separate experiments, paired, one-sided t-test, ns (not significant), *p<0.05).
F. The dose-response standardization experiments with series of concentrations of BTC, TLR2/4 ligands on THP-1 cells before identifying the lowest concentration for combination of all three together. The well-known cytokines were chosen as markers of gene expression with treatments (IL6 and CCL2; 5-8 experiments, paired, one-sided t-test, ns (not significant), *p<0.05, ** p<0.001, *** p<0.005, ****p<0.0001).
G. A heatmap from gene list derived from the enrichment analysis of mitochondria, in summary metric for BTC/TLR2/4 ligand treatment effects in THP1 cells revered by DHA treatment *in vivo* model. The color scale is indicated from high expression of the genes in red to low in blue.
H. A summary metric bar graph for BTC/TLR2/4 ligand treatment effects in THP1 cells revered by DHA treatment in vivo model from the enrichment analysis.
I. The integrin (ITGA6 and ITGA9) expression in the NASH preventive model is shown in bar graphs colored by treatment effects (Data are mean +/- SD, N=8 mice/treatment group). (Ordinary One-way ANOVA, with multiple comparisons test with WD+O, ns (not significant), *p<0.05, ** p<0.001, *** p<0.005, ****p<0.0001) and in THP-1 cells treated with BTC and or TLR2/4 ligands (N=5 experiments, paired, one-sided t-test, ns (not significant), *p<0.05).

Appendix Table S1

Legend: THP-1 cells response to TLR and BTC stimulation was assessed by qRT-PCR, the primer sequences as follows.

Gene	Forward	Reverse	Organism
IL8	ACTCCAAACCTTTCCACCCCA	CCCTCTGCACCCAGTTTTCCT	Human
TMEM59	GCTTCATAACCTCTTCATGGAC	GGATTTCTGGCTTAGACTGGA	Human
CCL2/MCP-1	CCCCAGTCACCTGCTGTTAT	AGATCTCCTTGGCCACAATG	Human
