# Supplementary material for: Multi‐omic network analysis identified betacellulin as a novel target of omega‐3 fatty acid attenuation of western diet‐induced nonalcoholic steatohepatitis
Source: EMBO Mol Med. 2023 Oct 20;15(11):e18367. doi: 10.15252/emmm.202318367 (PMC10630881; doi:10.15252/emmm.202318367)
Supplement: Supplementary file 2 — Expanded View Figures PDF [file EMMM-15-e18367-s001.pdf]

Expanded View Figures

Figure EV1. Extent of DHA reversal effects are significantly higher than those of EPA.

A–C The Log_2FC of EPA & DHA gene expression (A) and lipids and metabolites (B, C) show the extent of DHA reversal effects as significantly higher than EPA though similar in profile. The up and down regulation are shown in separate plots for clarity (A, B) (paired, two-sided *t*-test, ns [not significant], ****$P < 0.0001$).

C Scatterplot of fold change differences between WD + O and EPA (*x*-axis) or DHA (*y*-axis) treated mice with number of lipids and metabolites regulated similarly by DHA & EPA displayed (Pearson's Chi-squared test, **$P < 0.004$).

D Heatmap of differentially expressed genes in individual mice (WD + O vs. RD fed mice and organized by treatment effect category: DHA, EPA, or EPA & DHA). Data shown are the log transformed quantile normalized expression for each gene per prevention group. Row max is displayed as red, row min is displayed as blue.

 © 2023 The Authors

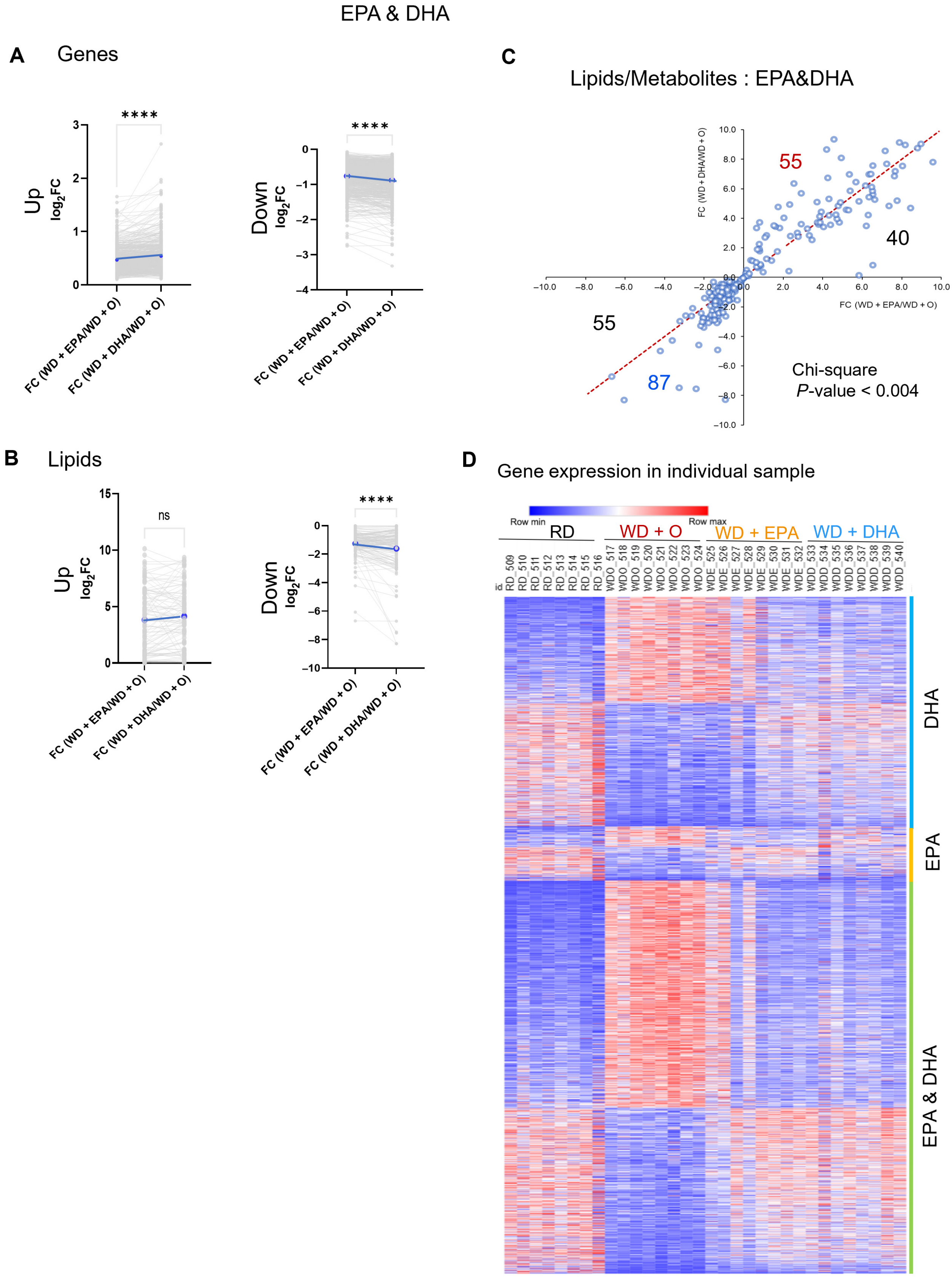
EPA & DHA
A
Genes
Up
log_2FC
Down
****
FC (WD + EPA/WD + O)
FC (WD + DHA/WD + O)
B
Lipids
ns
C
Lipids/Metabolites : EPA&DHA
FC (WD + DHA/WD + O)
FC (WD + EPA/WD + O)
55
40
55
87
Chi-square
P-value < 0.004
D
Gene expression in individual sample
Row min
Row max
RD
WD + O
WD + EPA
WD + DHA
DHA
EPA
EPA & DHA

Figure EV1.

Figure EV2. Interrogation of network model from the multi-omic NASH data.

A An outline with the steps involved in deriving, analyzing and interrogation of network model from the multi-omic NASH data (see Materials and Methods; GE: gene expression; P: Phenotype/Anthropometric/Biochemical data; LM: Lipids/Metabolites; PUC: Proportion of unexpected correlation). The distribution of treatment effects from the NASH preventive model among the network parameters is shown in the right panel.

B Outline of cell–cell interactions used to calculate the interaction BiBCs (see Materials and Methods). The nodes represent all genes part of cell types as a cluster in the network and edges are the interaction strength among the nodes. The node size indicates the number of genes represented by the cell type; color chart is proportional to the treatment effects in each cell type.

C Box plot of maximum cell–cell interaction BiBC for the genes belonging to each treatment effect category (DHA [blue], EPA&DHA [green], EPA [orange] and no category [gray]). From the network cell–cell BiBC analysis, genes regulated by DHA, DHA&EPA have higher BiBC that indicates a higher contribution to cell–cell communication than genes regulated by EPA.

D Bar plots for abundance of top BiBC lipids, shown are the PGs (cardiolipin precursors) and SM in NASH preventive study (Data are mean ± SD, $N = 8$ mice/treatment group; Ordinary One-way ANOVA, multiple comparisons test with WD + O, ***$P < 0.005$, ****$P < 0.0001$).

 © 2023 The Authors

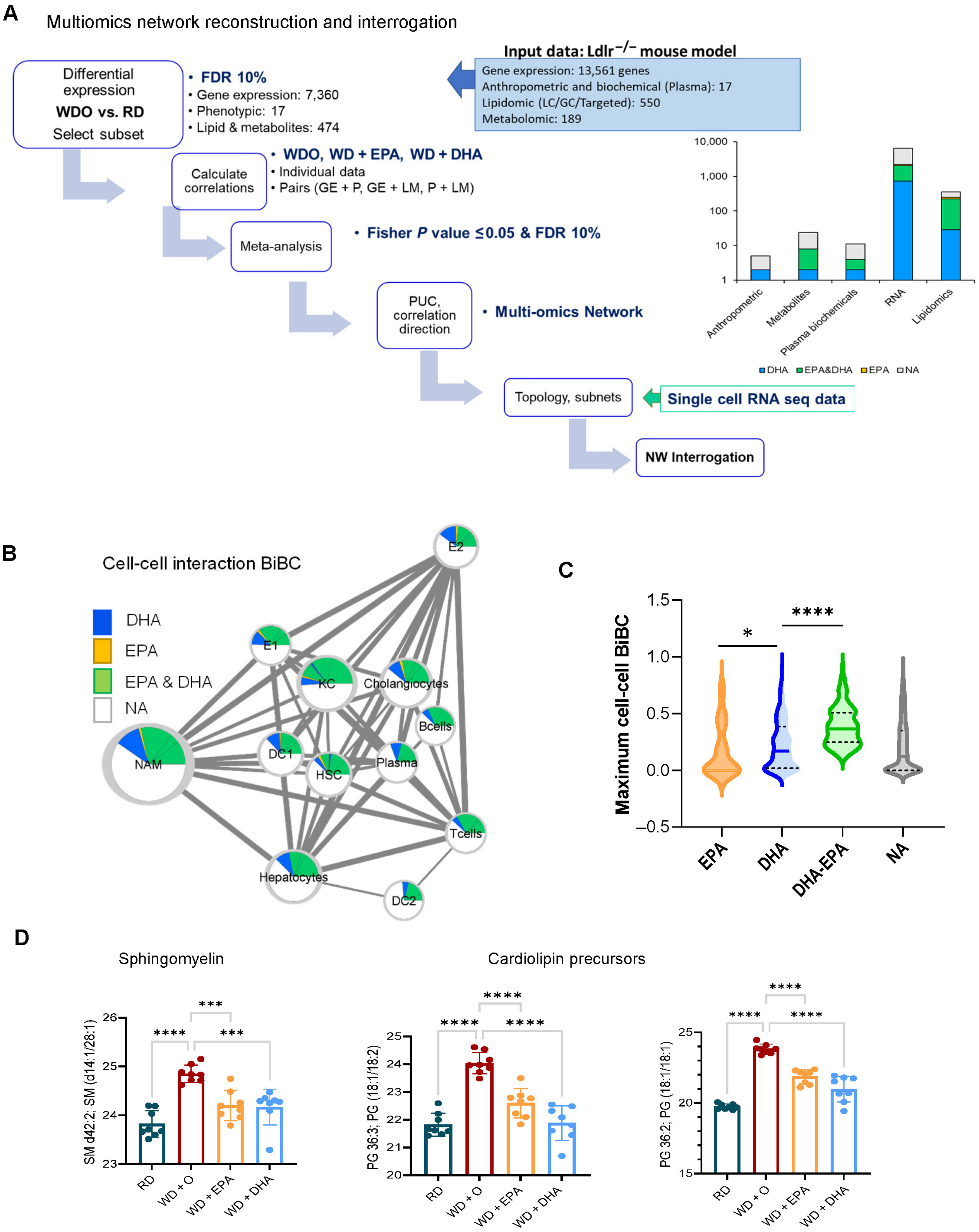
A
Multiomics network reconstruction and interrogation
Differential expression WDO vs. RD Select subset
• FDR 10%
• Gene expression: 7,360
• Phenotypic: 17
• Lipid & metabolites: 474
Input data: Ldlr−/− mouse model
Gene expression: 13,561 genes
Anthropometric and biochemical (Plasma): 17
Lipidomic (LC/GC/Targeted): 550
Metabolomic: 189
Calculate correlations
• WDO, WD + EPA, WD + DHA
• Individual data
• Pairs (GE + P, GE + LM, P + LM)
Meta-analysis
• Fisher P value ≤0.05 & FDR 10%
PUC, correlation direction
• Multi-omics Network
Topology, subnets
Single cell RNA seq data
NW Interrogation
Anthropometric
Metabolites
Plasma biochemicals
RNA
Lipidomics
DHA EPA&DHA EPA NA
B
Cell-cell interaction BiBC
DHA
EPA
EPA & DHA
NA
E2
E1
KC
Cholangiocytes
Bcells
NAM
DC1
HSC
Plasma
Tcells
Hepatocytes
DC2
C
Maximum cell-cell BiBC
EPA
DHA
DHA-EPA
NA
D
Sphingomyelin
Cardiolipin precursors
SM d42:2; SM (d14:1/28:1)
PG 36:3; PG (18:1/18:2)
PG 36:2; PG (18:1/18:1)
RD
WD + O
WD + EPA
WD + DHA

Figure EV2.

Figure EV3. Liver cancer meta-analysis and network interrogation points to significance of BTC.

A Outline for human liver cancer meta-analysis for the orthologous genes representing the NASH mouse model. Right panel shows the heatmap of individual human datasets for selected genes in the meta-analysis (with treatment effects in NASH model).

B The bar graph of gene set enrichment analysis using GSEA (KEGG pathways) for the human cancer meta-analysis genes with DHA effects in mouse models. Data are displayed as $-\log_{10}$(*P*-value).

C The gene expression for BTC-EGFR-ERBB pathway and cell cycle related genes in the NASH mouse preventive and treatment models. The color scale is indicated from high expression of the genes in red to low in blue.

D Distribution of Btc BiBC values calculated between DHA treatment reversed genes and metabolomic/lipidomic data (*x*-axis) and DHA controlled genes and anthropometric data (*y*-axis) in 5,000 random networks. Dark regions represent areas where a calculated Btc BiBC value is more likely to be found due to random chance. The probability of finding an actual Btc BiBC value equal to or higher than those seen in the random networks (43/5,000) is 0.009.

E Violin plot of the BiBC of Btc between DHA reversed genes and anthropometric data in 5,000 random networks compared to its actual BiBC value in the reconstructed network (one sample Wilcoxon test $P < 1 * 10^{-15}$).

© 2023 The Authors

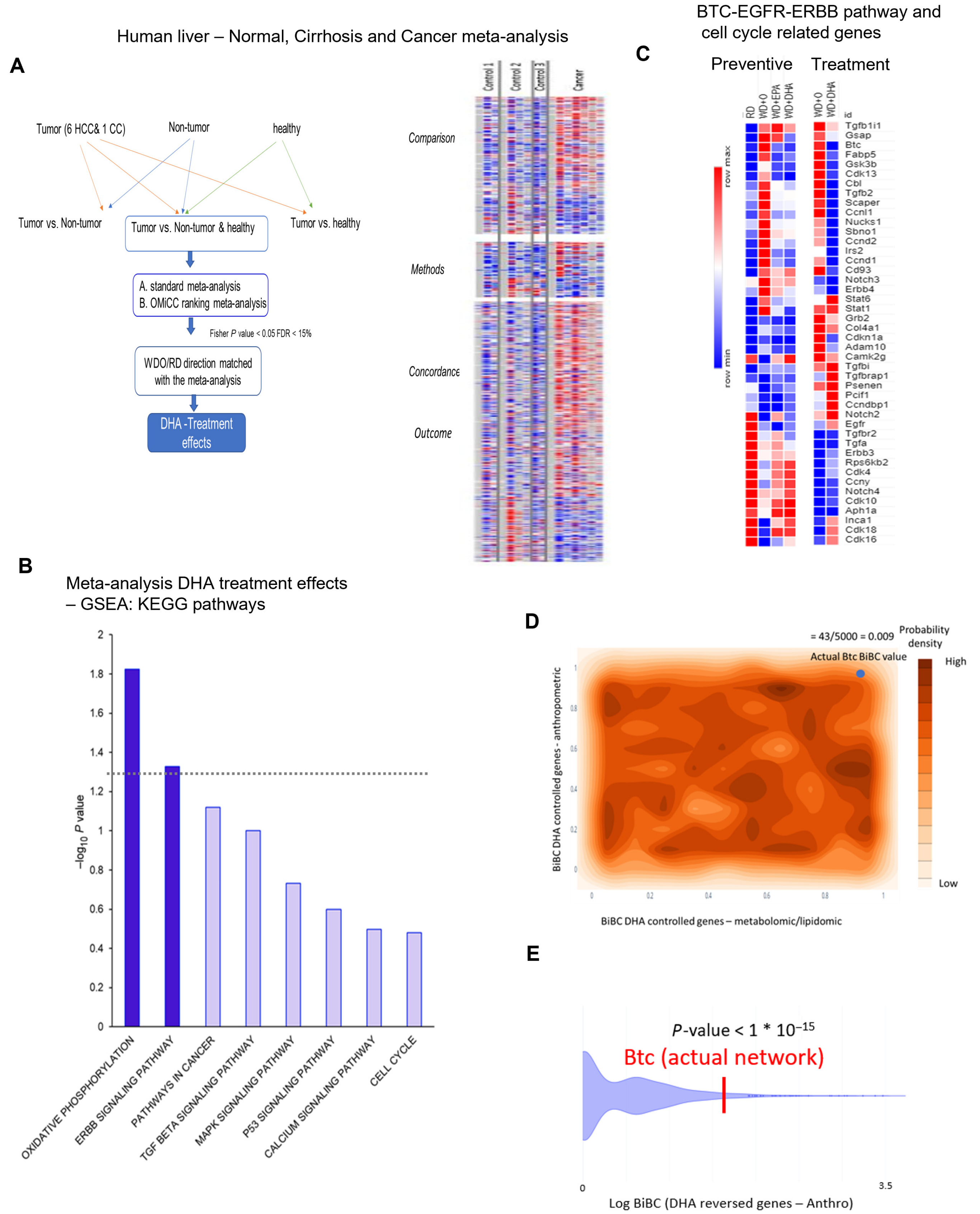
Human liver – Normal, Cirrhosis and Cancer meta-analysis
A
Tumor (6 HCC& 1 CC)
Non-tumor
healthy
Tumor vs. Non-tumor
Tumor vs. Non-tumor & healthy
Tumor vs. healthy
A. standard meta-analysis
B. OMiCC ranking meta-analysis
Fisher P value < 0.05 FDR < 15%
WDO/RD direction matched with the meta-analysis
DHA -Treatment effects
Control 1
Control 2
Control 3
Cancer
Comparison
Methods
Concordance
Outcome
C
BTC-EGFR-ERBB pathway and cell cycle related genes
Preventive
Treatment
RD
WD+O
WD+EPA
WD+DHA
WD+O
WD+DHA
row max
row min
id
Tgfb1i1
Gsap
Btc
Fabp5
Gsk3b
Cdk13
Cbl
Tgfb2
Scaper
Ccnl1
Nucks1
Sbno1
Ccnd2
Irs2
Ccnd1
Cd93
Notch3
Erbb4
Stat6
Stat1
Grb2
Col4a1
Cdkn1a
Adam10
Camk2g
Tgfbi
Tgfbrap1
Psenen
Pcif1
Ccndbp1
Notch2
Egfr
Tgfbr2
Tgfa
Erbb3
Rps6kb2
Cdk4
Ccny
Notch4
Cdk10
Aph1a
Inca1
Cdk18
Cdk16
B
Meta-analysis DHA treatment effects – GSEA: KEGG pathways
−log10 P value
OXIDATIVE PHOSPHORYLATION
ERBB SIGNALING PATHWAY
PATHWAYS IN CANCER
TGF BETA SIGNALING PATHWAY
MAPK SIGNALING PATHWAY
P53 SIGNALING PATHWAY
CALCIUM SIGNALING PATHWAY
CELL CYCLE
D
= 43/5000 = 0.009
Actual Btc BiBC value
Probability density
High
Low
BiBC DHA controlled genes - anthropometric
BiBC DHA controlled genes – metabolomic/lipidomic
E
P-value < 1 * 10−15
Btc (actual network)
0
3.5
Log BiBC (DHA reversed genes – Anthro)

Figure EV3.

Figure EV4. BTC and other Ligand-Receptor interaction network with DHA/EPA effects in NASH preventive mouse model.

A NASH Ligand–Receptor network with DHA/EPA effects in NASH preventive mouse model. The individual cells expressing the genes inferred from single cell RNA sequence reanalysis are overlayed and cell specific ligand–receptor interactions are shown. Nodes are shown as diamonds (ligands), or hexagon (receptors) and edges are the correlation between the nodes. Node color is according to the treatment effect. The thickness of the node is the cell–cell interaction BiBC, higher the better and is shown thicker. Each cell type and their respective genes are colored differently as labeled. The genes belonging to other cells in addition to specific cells are shown as part of 'Not assigned' group.

B The normalized Btc expression form others experiments (Left panel; Control and Nash fed with high fat high sugar mouse model; GSE197884; Mann–Whitney test, One-tailed, $N = 3$). (Right panel) The normalized Btc expression form other datasets (GSE222576; Control and CCl4 treated liver fibrosis mouse model; Ordinary one-way ANOVA, $*P < 0.05$, $**P < 0.005$).

 © 2023 The Authors

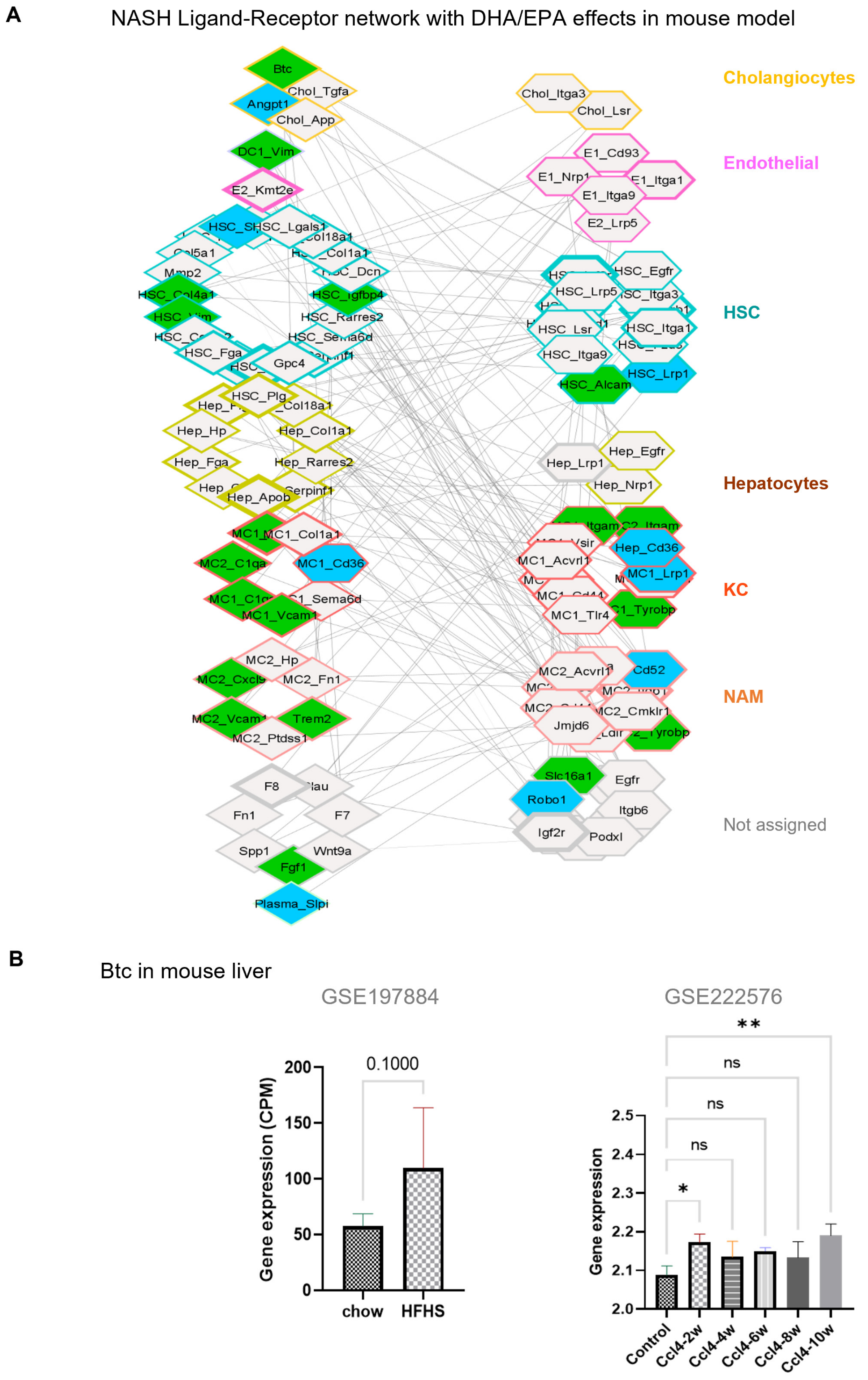
A
NASH Ligand-Receptor network with DHA/EPA effects in mouse model
Cholangiocytes
Endothelial
HSC
Hepatocytes
KC
NAM
Not assigned
Btc
Chol_Tgfa
Angpt1
Chol_App
DC1_Vim
E2_Kmt2e
HSC_Lgals1
Mmp2
HSC_Col4a1
HSC_Fga
Gpc4
HSC_Rarres2
HSC_Igfbp4
HSC_Plg
Hep_Hp
Hep_Fga
Hep_Col1a1
Hep_Rarres2
Hep_Apob
MC1_Col1a1
MC2_C1qa
MC1_Cd36
MC1_Vcam1
MC2_Hp
MC2_Cxcl9
MC2_Fn1
MC2_Vcam1
Trem2
MC2_Ptdss1
F8
Fn1
F7
Spp1
Wnt9a
Fgf1
Plasma_Slpi
Chol_Itga3
Chol_Lsr
E1_Cd93
E1_Nrp1
E1_Itga1
E1_Itga9
E2_Lrp5
HSC_Egfr
HSC_Lrp5
HSC_Itga3
HSC_Lsr
HSC_Itga1
HSC_Itga9
HSC_Lrp1
HSC_Alcam
Hep_Egfr
Hep_Lrp1
Hep_Nrp1
Hep_Cd36
MC1_Acvrl1
MC1_Lrp1
MC1_Tlr4
MC2_Acvrl1
Cd52
MC2_Cmklr1
Jmjd6
Slc16a1
Egfr
Robo1
Itgb6
Igf2r
Podxl
B
Btc in mouse liver
GSE197884
Gene expression (CPM)
0.1000
chow
HFHS
GSE222576
Gene expression
**
ns
ns
ns
*
Control
Ccl4-2w
Ccl4-4w
Ccl4-6w
Ccl4-8w
Ccl4-10w

Figure EV4.

Figure EV5. Potential mechanism of BTC regulation by the ω3 PUFA.

A The representation of network model and network interrogation to identify genes (nodes) top ranked according to Bipartite betweenness centrality (BiBC) and degree which potentially mediate effects of ω3 PUFA lipids on BTC-dependent gene expression profile. The legend for nodes is shown.

B The scheme of analysis to identify upstream regulator of Btc. The three modules of analysis with experimental data, network interrogation and identification of transcription regulator for Btc in Cholangiocytes. Identified transcription factors are labeled. Predicted binding site and motif for Foxo3 mediated transcription regulation in mice and human for Btc gene expression in the liver cholangiocytes is shown.

C Foxo3 gene expression *in vivo*. DHA reversed the gene expression significantly in the *in vivo* experimental model both in Preventive & Treatment models (Data are displayed as mean ± SD, $N = 8$ mice/group (preventative study) or $N = 5$ or 6 mice per group (treatment study); Ordinary One-way ANOVA, multiple comparisons test of reference diet (chow, RD), western diet + DHA (WD + DHA), western diet + EPA (WD + EPA) each with western diet + olive oil (WD + O), ns (not significant), $^{*}P < 0.05$, $^{***}P < 0.005$).

© 2023 The Authors

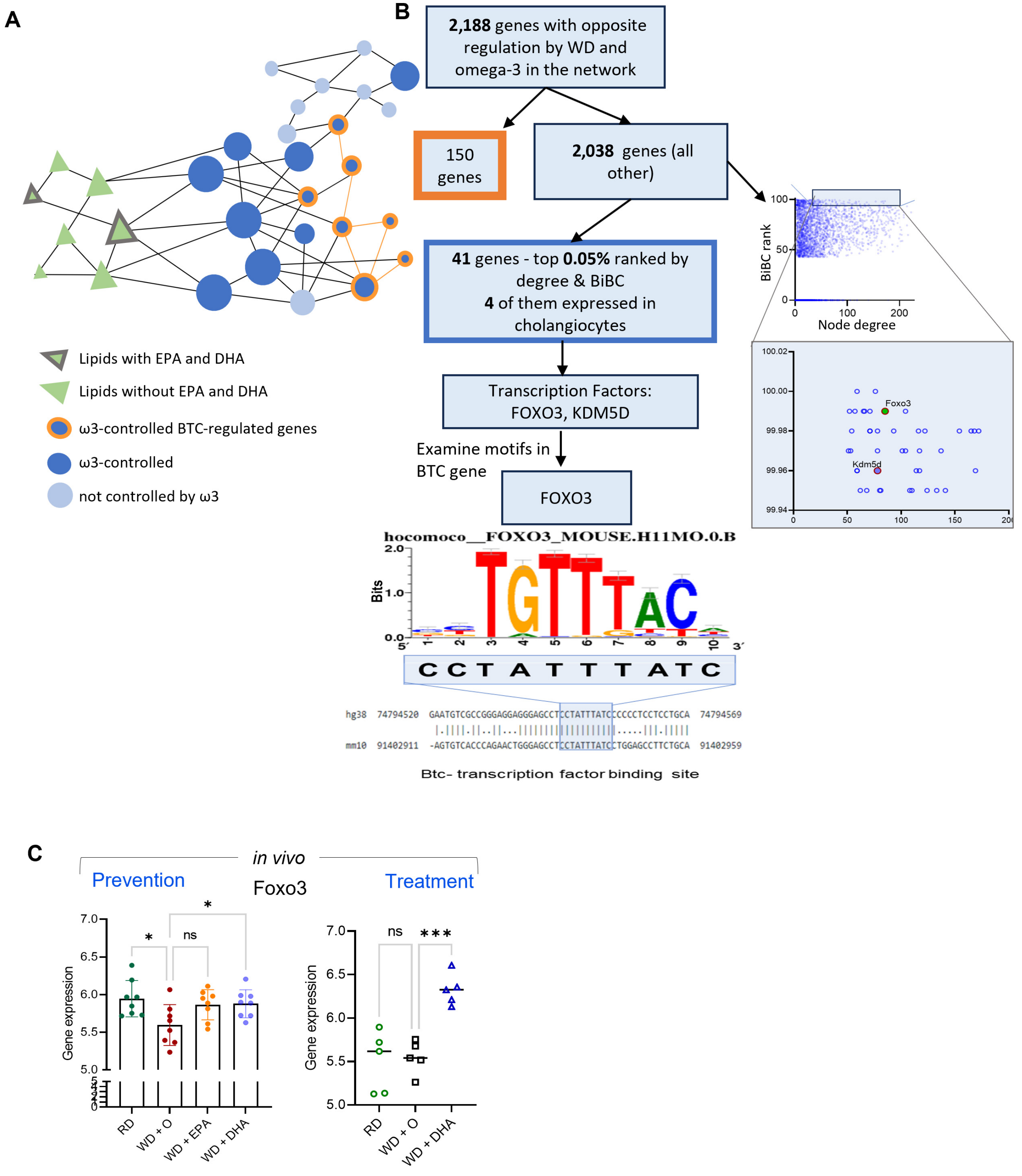
A
Lipids with EPA and DHA
Lipids without EPA and DHA
ω3-controlled BTC-regulated genes
ω3-controlled
not controlled by ω3
B
2,188 genes with opposite regulation by WD and omega-3 in the network
150 genes
2,038 genes (all other)
41 genes - top 0.05% ranked by degree & BiBC
4 of them expressed in cholangiocytes
Transcription Factors: FOXO3, KDM5D
Examine motifs in BTC gene
FOXO3
BiBC rank
Node degree
Foxo3
Kdm5d
hocomoco__FOXO3_MOUSE.H11MO.0.B
Bits
CCTATTTATC
hg38 74794520 GAATGTCGCCGGGAGGAGGGAGCCTCCTATTTATCCCCCTCCTCCTGCA 74794569
mm10 91402911 -AGTGTCACCCAGAACTGGGAGCCTCCTATTTATCCTGGAGCCTTCTGCA 91402959
Btc- transcription factor binding site
C
in vivo
Prevention
Treatment
Foxo3
Gene expression
RD
WD + O
WD + EPA
WD + DHA
ns
*
***

Figure EV5.
