# Supplementary material for: Multi‐omic network analysis identified betacellulin as a novel target of omega‐3 fatty acid attenuation of western diet‐induced nonalcoholic steatohepatitis
Source: EMBO Mol Med. 2023 Oct 20;15(11):e18367. doi: 10.15252/emmm.202318367 (PMC10630881; doi:10.15252/emmm.202318367)
Supplement: Supplementary file 11 — PDF+ [file EMMM-15-e18367-s008.pdf]

Article

TRANSPARENT PROCESS
OPEN ACCESS

EMBO Molecular Medicine

Multi-omic network analysis identified betacellulin as a novel target of omega-3 fatty acid attenuation of western diet-induced nonalcoholic steatohepatitis

Jyothi Padiadpu[1], Manuel Garcia-Jaramillo[2,†], Nolan K Newman[1,†], Jacob W Pederson[3,†], Richard Rodrigues[1,4], Zhipeng Li[3], Sehajvir Singh[1], Philip Monnier[1], Giorgio Trinchieri[4], Kevin Brown[1,5], Amiran K Dzutsev[4], Natalia Shulzhenko[3,*], Donald B Jump[6,**] & Andrey Morgun[1,***]

Abstract

Clinical and preclinical studies established that supplementing diets with ω3 polyunsaturated fatty acids (PUFA) can reduce hepatic dysfunction in nonalcoholic steatohepatitis (NASH) but molecular underpinnings of this action were elusive. Herein, we used multi-omic network analysis that unveiled critical molecular pathways involved in ω3 PUFA effects in a preclinical mouse model of western diet induced NASH. Since NASH is a precursor of liver cancer, we also performed meta-analysis of human liver cancer transcriptomes that uncovered betacellulin as a key EGFR-binding protein upregulated in liver cancer and downregulated by ω3 PUFAs in animals and humans with NASH. We then confirmed that betacellulin acts by promoting proliferation of quiescent hepatic stellate cells, inducing transforming growth factor–β2 and increasing collagen production. When used in combination with TLR2/4 agonists, betacellulin upregulated integrins in macrophages thereby potentiating inflammation and fibrosis. Taken together, our results suggest that suppression of betacellulin is one of the key mechanisms associated with anti-inflammatory and anti-fibrotic effects of ω3 PUFA on NASH.

Keywords betacellulin; docosahexaenoic acid; multi-omic network; nonalcoholic steatohepatitis; ω3 PUFA
Subject Categories Digestive System; Metabolism
DOI 10.15252/emmm.202318367 | Received 19 July 2023 | Revised 19 September 2023 | Accepted 21 September 2023 | Published online xx xxxx 2023
EMBO Mol Med (2023) 15: e18367

Introduction

Metabolic diseases associated with obesity have increased to epidemic proportions in recent years and are one of the leading causes of morbidity and mortality (Konerman *et al*, 2018; Dufour *et al*, 2022). Metabolic-associated fatty liver disease (MAFLD) or nonalcoholic steatohepatitis (NASH), type 2 diabetes and cardiovascular diseases are all associated with obesity and a sedentary lifestyle (Marjot *et al*, 2020; Lazarus *et al*, 2022). About 80 million adults and 13 million children are obese in the US alone. Among these, 60 percent of patients with body mass index > 30 have evidence of liver steatosis (Jump *et al*, 2015; Dufour *et al*, 2022). NASH is a progressive form of nonalcoholic fatty liver disease (NAFLD) and is a major risk factor for cirrhosis, hepatocellular carcinoma (HCC) and liver failure. While treatments to manage the co-morbidities associated with NASH, i.e., obesity and type 2 diabetes are available, NASH has no specific FDA-approved treatment. Thus, lifestyle modifications and dietary interventions are the current options available to NASH patients. Most if not all drugs which targeted individual molecules or specific pathways have failed to significantly improve the NASH patient (Neuschwander-Tetri, 2020; Pfister *et al*, 2021; Ampuero *et al*, 2022; Dufour *et al*, 2022). This strategy that has been effective in the treatment of other diseases might not be adequate for NAFLD/NASH therapy because it does not address entirely the complexity of this disease and may miss the master regulators involved in disease onset and progression.

1 College of Pharmacy, Oregon State University, Corvallis, OR, USA
2 Department of Environmental and Molecular Toxicology, Oregon State University, Corvallis, OR, USA
3 Carlson College of Veterinary Medicine, Oregon State University, Corvallis, OR, USA
4 Cancer and Inflammation Program, Center for Cancer Research, National Cancer Institute, National Institutes of Health, Bethesda, MD, USA
5 School of Chemical, Biological, and Environmental Engineering, Oregon State University, Corvallis, OR, USA
6 Nutrition Program, School of Biological and Population Health Sciences, Linus Pauling Institute, Oregon State University, Corvallis, OR, USA
*Corresponding author. Tel: +1 541 737 1051; E-mail: shulzhen@oregonstate.edu
**Corresponding author. Tel: +1 541 737 4007; E-mail: donald.jump@oregonstate.edu
***Corresponding author. Tel: +1 541 737 8047; E-mail: morguna@oregonstate.edu
[†]These authors contributed equally to this work

© 2023 The Authors. Published under the terms of the CC BY 4.0 license

Omega-3 polyunsaturated fatty acids (ω3 PUFA) are known to be consistently lower in livers of NASH patients when compared to healthy patients or patients with benign steatosis (Burke *et al*, 1999; Fridén *et al*, 2021). This prompted us to hypothesize that dietary supplementation with ω3 PUFA would restore liver functions. Indeed, this strategy was very successful in a preclinical mouse model, not only reducing liver steatosis but also in attenuating western diet-induced hepatic fibrosis (Depner *et al*, 2013a,b; Jump *et al*, 2015). Moreover, ω3 fatty acid treatment of children and adults with NAFLD have demonstrated these dietary lipids reduce hepatic steatosis and hepatic injury (Iannelli *et al*, 2013; Spooner & Jump, 2019). While it is well established that ω3 PUFA have the capacity to regulate hepatic mechanisms controlling fatty acid synthesis and oxidation, as well as inflammation, it is less clear if these pathways form the extent of ω3 fatty acid regulation of hepatic function (Jump *et al*, 2018). Despite several studies demonstrating the therapeutic effects of ω3 PUFA in NAFLD/NASH models the mechanism of action has been elusive. Nevertheless, it is important to note that many studies demonstrated diverse effects of ω3 in the liver ranging from immune-modulatory activities (Gutiérrez *et al*, 2019) and improvement of oxidative stress (Yang *et al*, 2019) to structural effects related to incorporation of phospholipids into the mitochondrial membrane, altogether with potentially positive impact on liver function. In addition to the effects of ω3 PUFA on hepatic cells, recent studies suggest that ω3 PUFA can alter gut microbiota, a known player in the pathogenesis of NAFLD/NASH (Watson *et al*, 2018). Furthermore, preclinical and clinical studies demonstrated that docosahexaenoic acid (DHA, 22:6, ω3) might be a more efficient agent than eicosapentaenoic acid (EPA, 20:5, ω3) in preventing and treating NAFLD (Depner *et al*, 2013a,b; Spooner & Jump, 2019).

Thus, while there are many studies describing different molecular and cellular effects of ω3 fatty acids (Hodson *et al*, 2017; Zöhrer *et al*, 2017; Musa-Veloso *et al*, 2018; Okada *et al*, 2018; Tobin *et al*, 2018; Šmíd *et al*, 2022) and some evidence that they may be an effective therapy for NAFLD/NASH, the key mechanisms of how they improve liver health is unknown. As such, we used a comprehensive unbiased systems approach to answer these questions. For this, we evaluated the liver transcriptome, metabolome and lipidome and assessed causal inferences via multi-omic network analysis to identify prospective mechanism operating in the diseased liver that were restored by EPA and/or DHA. Since NASH is one of the precursors of liver cancer, we also performed a meta-analysis of human liver cancer to evaluate which aspects of NASH pathogenesis leading to cancer are reversed by ω3 fatty acids. Together, our studies pointed to betacellulin (BTC), one of several epidermal growth factor receptor (EGFR) agonists, as a master regulatory molecule that was downregulated by ω3 PUFA in the NASH liver. We further validated the impact of BTC in cell culture experiments that established TGFβ-2 and integrins as the main downstream molecular targets of BTC in human hepatic stellate cells and macrophages, respectively. Suppression of these pathways specifically by DHA leads to attenuated fibrosis in NASH. Thus, our study disclosed an entirely novel mechanism for ω3 fatty acid control of hepatic function and its beneficial action against detrimental molecular events in the liver leading to NASH.

Results

DHA reverses the effects of WD more effectively than EPA

We first performed a comprehensive analysis of molecular changes contributing to prevention of NAFLD/NASH by two ω3 PUFA, namely docosahexaenoic (DHA, 22:6, ω3) and eicosapentaenoic acid (EPA, 20:5, ω3). For this, we evaluated histological markers of NASH (Dataset EV1a), transcriptomic, metabolomic, and lipidomic changes caused by DHA and EPA in the whole tissue liver samples from *Ldlr*$^{-/-}$ mice fed a western diet (WD) with the addition (or not) of DHA or EPA (Depner *et al*, 2013a; Fig 1A–C, Dataset EV1b). To focus our analysis on disease features corrected by ω3 PUFA, we first established which changes induced by WD were reversed by DHA or EPA treatment (see Materials and Methods for details). We then established four categories of parameters: (i) regulated by DHA only (e.g., Cd36, Fig 1D top left); (ii) regulated by EPA only (e.g., Notch2, Fig 1D top right); (iii) regulated similarly by DHA and EPA (e.g., Cx3Cr1, Fig 1D bottom left); (iv) not regulated by either DHA or EPA (e.g., Egfr, Fig 1D bottom right). Although there was a large overlap between the effects of each ω3 PUFA, overall, DHA showed stronger effects than EPA in restoring alterations caused by WD. Specifically, while both EPA and DHA showed similar effects on 19% of the genes affected by disease, DHA alone reversed more genes (11%) than EPA alone (3%), (Fig 1B and C). In line with this result, focusing on genes regulated by both fatty acids (DHA and

Figure 1. NASH mouse model outlines all expression/omics data, DHA/EPA treatment effects on outcome.

A All omics data collected and shown in Fig 1A bar graph and its associated table was acquired using samples (N = 8/treatment group) from a preclinical NASH prevention study previously described (Depner *et al*, 2013a,b; Lytle *et al*, 2015). The comparison of the western diet + olive oil (WD + O) group vs. reference diet (chow, RD) group showing the differentially expressed genes or parameters with a P-value < 0.05 and FDR < 10% and those that have treatment effect reversal uniquely by DHA (blue), or EPA (orange) or similar with significance in both EPA and DHA is EPA&DHA (green).

B Heatmap of differentially expressed genes in WD + O vs. RD fed mice and organized by treatment effect category: DHA, EPA, or EPA & DHA. Data shown are the geometric mean expression for each gene per each treatment group. Row max is displayed as red, row min is displayed as blue.

C Heatmap of parameters (lipids and metabolites) in WD + O vs. RD fed mice and organized by treatment effect category (DHA, EPA, or DHA &EPA) that are significant at P-value < 0.05 and FDR < 0.1. Data shown are a geometric mean of each parameter measurement for each treatment group. Row max: red, row min: blue.

D Shown are selected representatives (Cd36, Notch2, Cx3cr and Egfr) from each category according to treatment effect (top left: DHA, top right: EPA, bottom left: EPA & DHA, bottom right: no treatment effect). Shown here is an example of one gene from each category (Data are mean ± SD, N = 8 mice/treatment group; One-way ANOVA, with multiple comparisons test with WD + O, ns (not significant), *P < 0.05, **P < 0.001, ***P < 0.005, ****P < 0.0001).

E Scatterplot of fold change differences between WD + O and DHA or EPA treated mice with number of genes regulated by DHA (Blue) or EPA (Orange) displayed (Pearson's Chi-squared test, ****P < 0.0001).

F Top enriched biological process changed by DHA treatment. Top + Blue bars: Induction by DHA, Bottom + Green bars; Repression by DHA. Bars show $-\log_{10}$(P-value) transformed values for visualization.

© 2023 The Authors

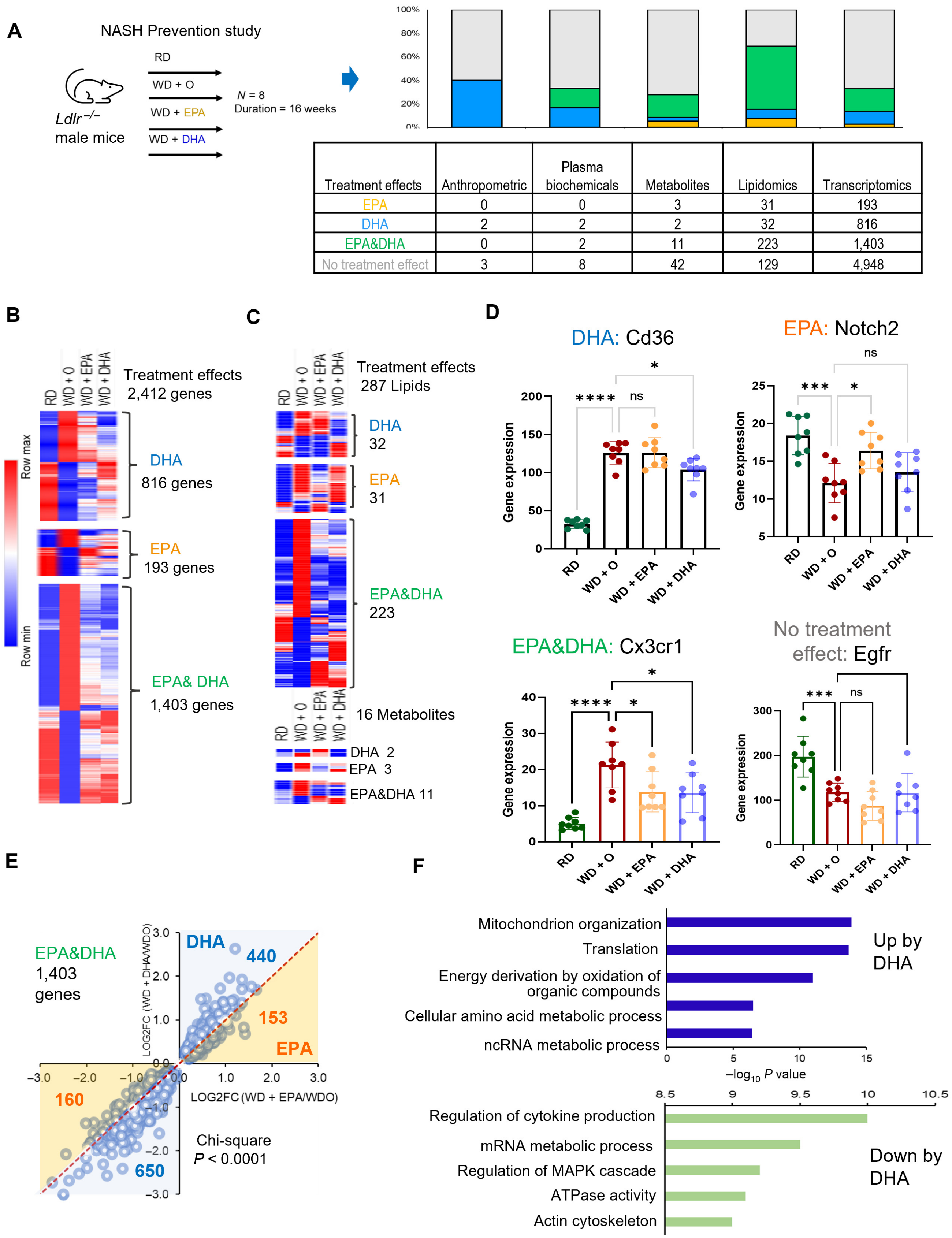

Treatment effects	Anthropometric	Plasma biochemicals	Metabolites	Lipidomics	Transcriptomics
EPA	0	0	3	31	193
DHA	2	2	2	32	816
EPA&DHA	0	2	11	223	1,403
No treatment effect	3	8	42	129	4,948

Figure 1.

EPA) we observed more pronounced changes by DHA than EPA ($P < 0.0001$; Figs 1E and EV1A and B). The genes upregulated by DHA were enriched for several functional categories: mitochondrial organization, translation, and energy derivation by oxidation of organic compounds were among the most prominent pathways affected. Regulation of cytokine production was the top enriched pathway among the downregulated genes (Fig 1F). Thus, the first step of transcriptome analysis indicated that DHA had a stronger effect than EPA in reversing damage inflicted by WD on the liver potentially by restoring mitochondrial function and inhibiting inflammation.

In the second largest omics data set, represented by lipidomes, we did not see differences in the number of lipids regulated by DHA or EPA and ω3 PUFA restored most lipids impaired by WD (Fig 1A and C). However, we detected a significantly stronger effect on lipids regulated by DHA vs. by EPA (Fig EV1C). Analysis of anthropometric features and plasma biochemicals also showed a more pronounced effect by DHA than EPA, as we previously reported (Depner *et al*, 2013a; Fig 1A). Overall, DHA demonstrated stronger effects than EPA in reversing WD-induced changes in both gene expression (Fig EV1D) and lipid concentrations.

Mapping effects of ω3 PUFA onto multi-omic network model of NASH and its cellular components

To investigate how changes in different omics data relate to each other and which of the effects are contributing to the effects of ω3 PUFA, we have reconstructed a multi-omic network model of NAFLD/NASH and mapped ω3 PUFA effects into this model (Fig EV2A). After filtering out data features that did not pass statistical (Dong *et al*, 2015) and causality (Yambartsev *et al*, 2016) criteria thresholds (see Materials and Methods), the multi-omic network consisted of 6,743 nodes connected by 80,811 edges. Specifically, the network included 6,346 gene transcripts, 5 anthropometric nodes, 11 plasma biochemicals, 357 lipids, and 24 metabolites (Fig 2A). We next identified clusters (sub-networks) and using functional enrichment analyses found that different subnetworks were enriched in different pathways, including mitochondrial organization, myeloid leukocyte activation, cell and mitochondrial membrane fluidity, remodeling, signaling, and energy metabolism. It also included processes such as macromolecule catabolic and fatty acid metabolic processes (Fig 2A, Dataset EV1b).

Multiple cell types in the liver contribute to NASH pathogenesis (Ramachandran *et al*, 2019; Xiong *et al*, 2019; Seidman *et al*, 2020) but which cells are responding to DHA treatment has not been comprehensively studied. Therefore, we mapped genes regulated by ω3 PUFA to cell type information using a previously published single cell RNA-seq dataset generated from diet-induced NASH mouse livers (Xiong *et al*, 2019). Among different cell types, most of the ω3 PUFA-regulated genes were assigned to one of the two major hepatic macrophage subpopulations including NASH-associated macrophages (NAM, 1,455 genes) and Kupffer-like cells (KC, 585 genes), named according to the previous study (Xiong *et al*, 2019). These were followed by hepatocytes, cholangiocytes, and hepatic stellate cells (522, 365, and 192 genes respectively; Fig 2B). In line with our initial observation (Fig 1A), we found that DHA reversed expression of a larger number of genes than EPA, irrespectively of the cell type (Fig 2B).

As a next step, we wanted to ensure that our model (i.e., multi-omic network enhanced with information about cell type assignment) is consistent with known effects of ω3 PUFA on NASH. For this, we analyzed connectivity related topological network properties, known as bipartite betweenness centrality (BiBC) that was shown by us (Morgun *et al*, 2015; Li *et al*, 2022) and others (Lam *et al*, 2021) as a metric reflective of causal relationships between nodes in a co-variation network. High ranked BiBC nodes represent parameters mediating impact of one part (e.g., module/cluster) of a network on another (Morgun *et al*, 2015; Li *et al*, 2022). Thus, we calculated cell–cell interaction BiBC based on the expression of genes assigned to different cells and affected or not by ω3 PUFA in NASH multi-omic network (Fig EV2B). The results showed that DHA regulated genes had higher BiBCs than those regulated only by EPA or unaffected by either fatty acid (Fig 2C; Fig EV2C). This result is in line with our previous observations of DHA being more potent than EPA in improving NASH (Lytle *et al*, 2017).

This result supported the general validity of the transcriptomic part of our network, thus, we asked which of the lipids/metabolites may have major contribution to cell–cell interactions in NASH. For this analysis, in addition to BiBC we accounted for node degree as this topological property has been the most prevalent network parameter in computational biology (Sorrells & Johnson, 2015; Choobdar *et al*, 2019) reflecting importance of the node in controlling direct and indirect neighbors and therefore corresponding biological function. Specifically, nodes of high degree (also called

Figure 2. Multi-omic network (NW) reconstructed to model NASH *in vivo* using the data from preventive model (see Fig EV2; Materials and Methods).

A The cytoscape visualization of the network has nodes (circles, rectangles) representing genes, lipids, metabolites, plasma biochemical, and anthropometric data (Depner *et al*, 2013a), and edges representing correlation in a color ranging light red to light blue depending on correlation (1 to −1). The nodes are colored based on their treatment effect category membership, with DHA (blue), EPA&DHA (green), EPA (orange) and no category (gray). Network clusters are based on infomap modules additionally characterized by gene and lipids functional enrichment.

B Bar plot of number of NW genes from each treatment category (DHA [blue], EPA [orange], or EPA&DHA [green]) with assignment to a given cell type. Subplot: figure shows t-SNE plot with all cell type clusters from a reanalyzed NASH mouse single cell RNA-seq dataset (Xiong *et al*, 2019) used in our study to assign cell type information. NAM- Nash associated macrophages, KC-Kupffer like cells, DC- dendritic cells, HSC- hepatic stellate cells and E- endothelial cells.

C Violin plot of average cell–cell interaction BiBC for the genes belonging to each treatment effect category (DHA [blue], EPA&DHA [green], EPA [orange] and no category [gray]) shown with $n = 141, 718, 1,299, 4,007$ genes respectively; solid lines indicate median, dashed line—quartiles; P values ** < 0.01; **** < 0.0001.

D The scatterplot shows the network cell–cell BiBC verses node degree for the top hepatic lipids in the NASH network. The figure insert is the structural representation of the lipid TG58:11 (TG 16:0/20:5/22:6) with DHA and EPA as two of its acyl chains from the NASH prevention study. These top hepatic lipids are shown with treatment effect category EPA&DHA (green).

E Bar plots for abundance of top BiBC lipids, shown are the triglycerides with DHA and EPA as acyl chains in NASH preventive study (Data are mean ± SD, $N = 8$ mice/treatment group; Ordinary One-way ANOVA, multiple comparisons test with WD + O, ns [not significant], *$P < 0.05$, **$P < 0.001$, ***$P < 0.005$, ****$P < 0.0001$).

 © 2023 The Authors

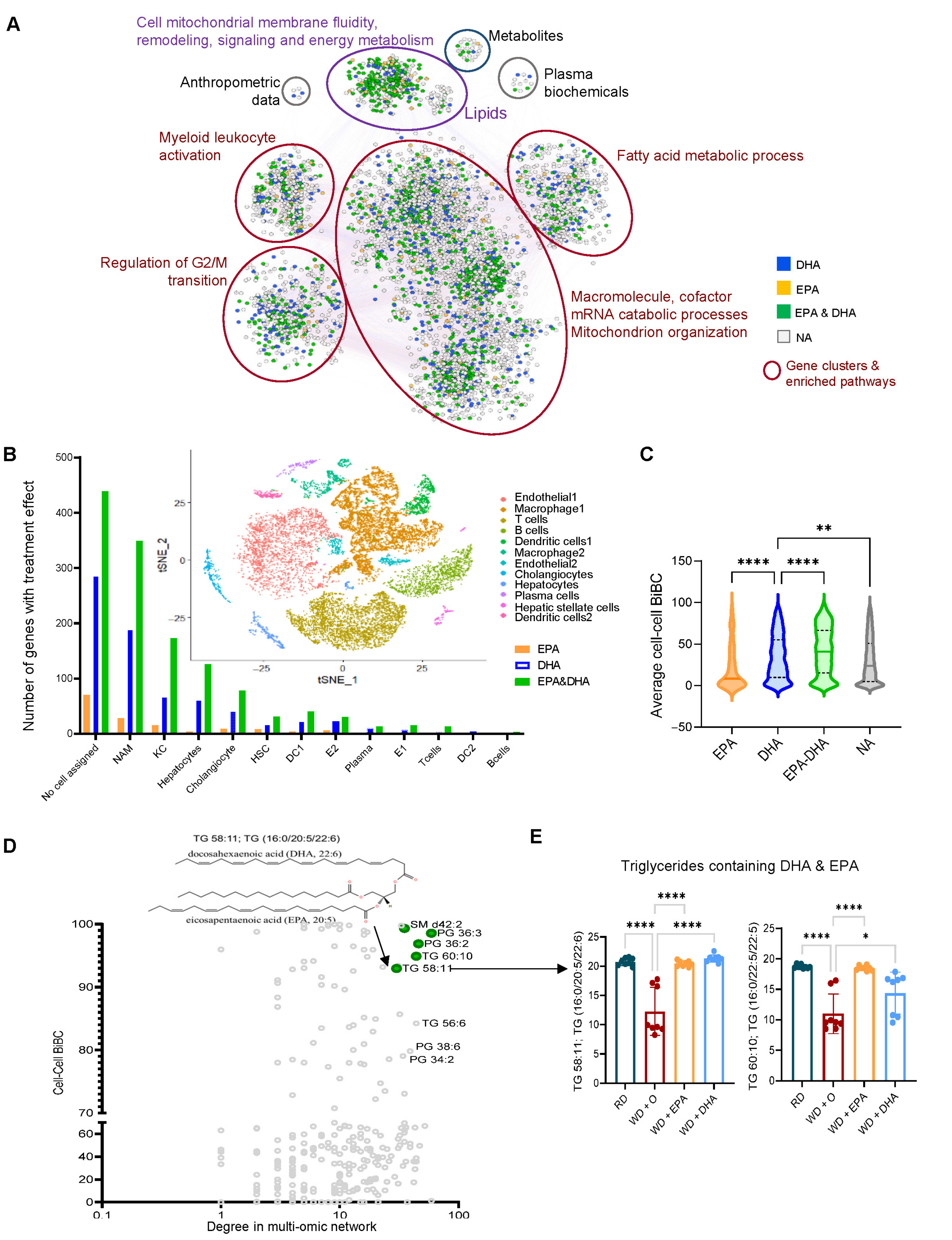
A
Cell mitochondrial membrane fluidity, remodeling, signaling and energy metabolism
Metabolites
Plasma biochemicals
Anthropometric data
Lipids
Myeloid leukocyte activation
Fatty acid metabolic process
Regulation of G2/M transition
Macromolecule, cofactor mRNA catabolic processes Mitochondrion organization
DHA
EPA
EPA & DHA
NA
Gene clusters & enriched pathways
B
Number of genes with treatment effect
No cell assigned
NAM
KC
Hepatocytes
Cholangiocyte
HSC
DC1
E2
Plasma
E1
Tcells
DC2
Bcells
tSNE_1
tSNE_2
Endothelial1
Macrophage1
T cells
B cells
Dendritic cells1
Macrophage2
Endothelial2
Cholangiocytes
Hepatocytes
Plasma cells
Hepatic stellate cells
Dendritic cells2
EPA
DHA
EPA&DHA
C
Average cell-cell BiBC
EPA
DHA
EPA-DHA
NA
D
TG 58:11; TG (16:0/20:5/22:6)
docosahexaenoic acid (DHA, 22:6)
eicosapentaenoic acid (EPA, 20:5)
SM d42:2
PG 36:3
PG 36:2
TG 60:10
TG 58:11
TG 56:6
PG 38:6
PG 34:2
Cell-Cell BiBC
Degree in multi-omic network
E
Triglycerides containing DHA & EPA
TG 58:11; TG (16:0/20:5/22:6)
TG 60:10; TG (16:0/22:5/22:5)
RD
WD + O
WD + EPA
WD + DHA

Figure 2.

hubs) generally represent master regulators of a part of the large network (clusters or subnetworks) in which these hubs are situated (Sorrells & Johnson, 2015; Choobdar *et al*, 2019).

We selected nodes from lipids/metabolite part of the network that were top ranked by BiBC and had high degree (top 10% for both parameters) and were also regulated by ω3 PUFA. We identified five lipids which represented < 2% of total lipids detected in our data. Importantly, two out of the five lipids (Figs 2D and EV2D) were triglycerides containing EPA and DHA (TG 58:11 and TG 60:10) as acyl chains (Fig 2E). Only 7 other lipids out of 357 had similar chemical composition (i.e., TG containing EPA/DHA) were detected by our lipidomic assay, thus making this finding being random virtually impossible ($P < 0.0001$). Thus, this analysis identified two lipids, which are drastically depleted in the liver during and have protective effect reversing NASH (Burke *et al*, 1999; Jump *et al*, 2015; Fridén *et al*, 2021). Although this result might be expected, it is nevertheless important as a "positive control" providing additional confidence in our network.

The other three lipids were phosphatidyl glycerol (PG) 36:3, PG 36:2 (cardiolipin precursors) and sphingomyelin (SM) d42:2, which are both main membrane lipids (Fig EV2D). Since top inferences from this network validated key previously known molecular aspects of ω3 PUFA action in NASH liver we can rely on this model for inference of new yet undiscovered aspects of this process.

Combining the NASH network with the meta-analysis of liver cancer identifies betacellulin as a key pathogenic regulator of NASH inhibited by DHA

NASH can progress to liver cirrhosis and cancer in humans (Samuel & Shulman, 2018; Anstee *et al*, 2019; Pfister *et al*, 2021). Furthermore, this was also shown in the mouse model used in this study (Chen *et al*, 2021). Although ω3 PUFA have been studied as a potential prevention strategy for colorectal cancer progression and other cancers (Van Blarigan *et al*, 2018; Dierge *et al*, 2021), there is still little understanding which cancer pathways can be inhibited by these fatty acids. Thus, as a next step, we mapped the molecular model of transcriptome alterations by DHA in liver tissue (Fig 2A) to transcriptomic alterations in human liver cancer. For this, we first performed a meta-analysis of human liver cancers (7 transcriptomic datasets with a total of 544 tumor [Hepatocellular carcinoma and Cholangiocarcinoma], 260 non-tumor, and 32 healthy patient liver samples) and established which genes were expressed concordantly by cancer and WD-induced alterations in our preclinical mouse model (Fig EV3A, Dataset EV2). Among 2,080 concordantly expressed genes between NASH and cancer, 22% (456 genes), 10% (221 genes) and 3% (56 genes) were reversed by DHA and EPA, DHA alone and EPA alone, respectively (Fig 3A left panel, Dataset EV3). While the current mouse study was designed to assess the effects of ω3 PUFA on preventing NASH (Prevention study), in another study (Lytle *et al*, 2017) we evaluated liver transcriptomes of DHA-treated mice with already established NASH (Treatment study). In this case, we observed that treatment effects of DHA cover ~ 54% (361 out of 677genes) of its preventive effects relevant for human liver cancer (Fig 3A right panel). These results suggest that DHA can potentially be used as cancer preventive strategy in patients with already established NAFLD/NASH.

Next, we asked which of the molecular pathways regulated in cancer and reversed by DHA in mice may mediate beneficial effects of DHA. For this, we combined gene enrichment analysis with BiBC to focus on genes with the largest impact on cell–cell interactions (see details in Materials and Methods). The top enriched pathway was Oxidative Phosphorylation with the well-known pathways such as TGFβ and p53 signaling being enriched to a lesser extent (Fig EV3B). One pathway, however, that stood out as highly enriched was the ERBB signaling pathway; it was also top ranked in mediating DHA-driven cell–cell interactions (i.e., BiBC; Figs 3B and EV3C). ERBBs are known homologs of EGFR, which are activated through binding to EGF and related members of the EGF family of growth factors. These include EGF-like ligands or cytokines that are comprised of at least 10 proteins including betacellulin, transforming growth factor-alpha (TGF-α), amphiregulin, HB-EGF, epiregulin, and neuregulins and the various other heregulins (Olayioye *et al*, 2000; Wieduwilt & Moasser, 2008; Chen *et al*, 2016).

Using our multi-omic network (including DHA affected and not affected genes in NASH), we ranked genes from the ERBB pathway based on their potential capacity to mediate effects of DHA and identified betacellulin (BTC), an alternative ligand of EGFR (Fig EV4A), as a top gene among the important genes (Grb2, Gsk3 Gsk3β/α, and Cbl) in the pathway (Fig 3C). Interestingly, EGFR itself, although not regulated by DHA was the second best potential regulatory gene for this pathway in NASH. To ensure statistical

Figure 3. Combining NASH network with meta-analysis of liver cancer identifies betacellulin as a key pathogenic regulator downregulated by DHA.

A The heatmap of genes from human liver cancer meta-analysis ordered by corresponding genes from the mouse NASH on the effect of EPA and DHA on prevention and treatment of NASH in mice (Depner *et al*, 2013a; Lytle *et al*, 2017).

B 3D scatter plot showing gene set enrichment analysis (GSEA) for the DHA effects with Rank score on the *x*-axis, GSEA $-\text{Log}_{10}$(*P*-value) on the *y*-axis, and cell–cell interaction BiBC on the *z*-axis. Relevant pathways are labeled in the figure with the (BTC)-ERBB pathway (red) ranked highly by all metrics.

C Scatterplot of network BiBCs between gene expression and anthropometric parameters plotted against cell–cell BiBCs. Members of the Btc-Erbb pathway genes are overlayed and indicate the importance of Btc-Erbb signaling in the NASH multi-omic network model with preventive effects. Each circle is a node in the network, filled circles (preventive effects) with red outer circle are part of the Btc-Erbb pathway.

D Bar plot of Btc gene expression in mouse livers from the prevention and treatment studies ($N = 5$ or 6/group) experiment. Data are mean ± SD, Ordinary One-way ANOVA, multiple comparisons test of reference diet (chow, RD), western diet + DHA (WD + DHA) with western diet + olive oil (WD + O), *$P < 0.05$, **$P < 0.001$, ***$P < 0.005$, ****$P < 0.0001$).

E BTC gene expression from meta-analysis of human normal and liver cancer datasets indicates a significant increase in liver cancer samples (Effect size FDR = 0.0011). The size of each individual dot represents the number of patients associated with the dataset with higher the number of patients darker the dot color, Normal (green) and Cancer (red; 7 transcriptomic datasets with a total of 544 tumor, 260 non-tumor, and 32 healthy patient liver samples).

F BTC gene expression in human datasets of NASH. *Left*: Control and NASH liver (GSE193080) with normal 3 patients and Nash with 32 patient samples (Welch's *t*-test, One-tailed, **$P < 0.001$). *Middle*: GSE48452, Control, $N = 14$, Obese Healthy, $N = 27$, Steatosis, $N = 14$, Nash, $N = 18$ (Mann–Whitney test, Two-tailed, *$P < 0.05$). *Right*: normalized Btc gene expression (GSE96971), NASH patients before and after 1 year of treatment with EPA and DHA, $N = 9$ (Paired *t*-test, One-tailed, *$P < 0.05$).

© 2023 The Authors

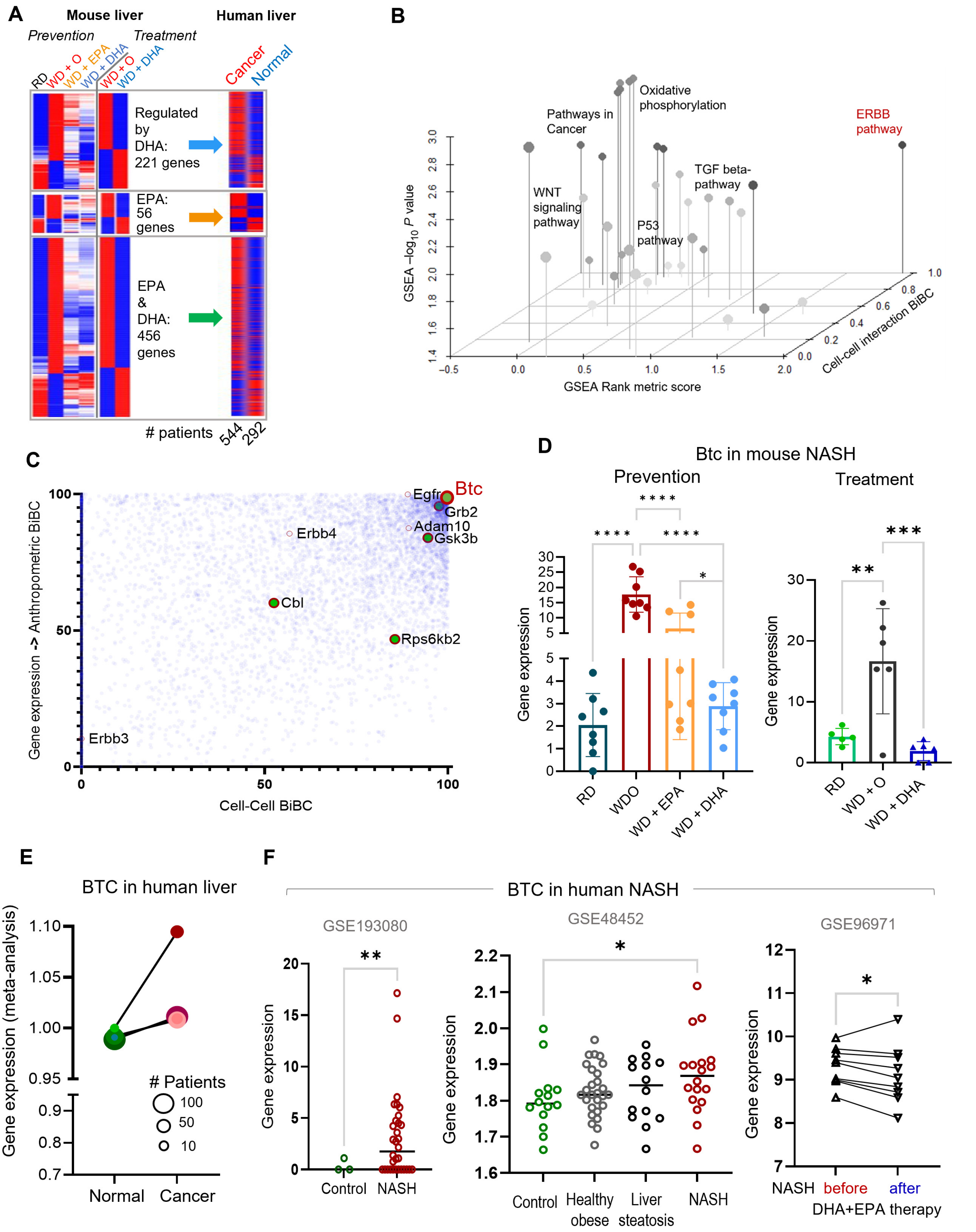
A
Mouse liver
Human liver
Prevention
Treatment
RD
WD + O
WD + EPA
WD + DHA
WD + O
WD + DHA
Cancer
Normal
Regulated by DHA: 221 genes
EPA: 56 genes
EPA & DHA: 456 genes
patients 544 292
B
GSEA −log10 P value
GSEA Rank metric score
Cell-cell interaction BiBC
Oxidative phosphorylation
Pathways in Cancer
ERBB pathway
TGF beta-pathway
WNT signaling pathway
P53 pathway
C
Gene expression -> Anthropometric BiBC
Cell-Cell BiBC
Egfr
Btc
Grb2
Erbb4
Adam10
Gsk3b
Cbl
Rps6kb2
Erbb3
D
Btc in mouse NASH
Prevention
Treatment
Gene expression
RD
WDO
WD + EPA
WD + DHA
RD
WD + O
WD + DHA
E
BTC in human liver
Gene expression (meta-analysis)
Patients
100
50
10
Normal
Cancer
F
BTC in human NASH
GSE193080
GSE48452
GSE96971
Gene expression
Control
NASH
Control
Healthy obese
Liver steatosis
NASH
NASH before after
DHA+EPA therapy

Figure 3.

robustness of BTC potential causal role reflected by its high BiBC rankings, we used two additional approaches (see Materials and Methods) that demonstrated very low probability of Btc being randomly highly ranked (with $P < 10^{-15}$ and probability density $P = 0.009$, respectively; Fig EV3D and E).

We next confirmed that Btc gene expression was increased in other models of NASH, liver cancer and fibrosis including a chemically induced disease (Green *et al*, 2022; Hammad *et al*, 2023; Fig EV4B), and it was downregulated by both EPA and DHA in our prevention model, albeit DHA had a stronger effect than EPA (Fig 3D, left panel).

In agreement with mouse models, Btc gene expression was increased in human hepatic carcinoma meta-analysis (Fig 3E) and in human NASH in two studies (Ahrens *et al*, 2013; Fujiwara *et al*, 2022; Fig 3F, Dataset EV4). Importantly, EPA + DHA treatment of patients with NASH for one year resulted in a significant decrease of Btc gene expression (Fig 3F).

BTC promotes NASH fibrosis via activating hepatic stellate cells to produce TGFβ-2

Given that BTC was predicted as a new target, central for effects of DHA on NASH, we next verified which cell types express BTC and its receptor (EGFR). For this, we integrated the network with available interaction information from ligand–receptor database (Abugessaisa *et al*, 2021) and single cell RNA-seq data (Xiong *et al*, 2019; Fig EV4A). We observed that cholangiocytes were the primary population of cells expressing BTC (Fig EV4A). Although its source is restricted to cholangiocytes, BTC's role as a secreted growth factor indicates it can act on many different types of neighboring cells (e.g., hepatocytes, macrophages, hepatic stellate cells and others) that express EGFR/ERBBs (Fig EV4A and B).

Among different liver cell types which can respond to BTC and proliferate during NASH progression, hepatic stellate cells (HSCs), frequently called mesenchymal cells in humans (Ramachandran *et al*, 2019; Carter & Friedman, 2022) produce collagens and represent a major contributor to hepatic fibrosis. Indeed, the number of mesenchymal cells counted in humans with cirrhosis is markedly higher than those of healthy livers (Appendix Fig S1C). Importantly, DHA prevents and reverses fibrosis in a NASH mouse model (Depner *et al*, 2013a; Lytle *et al*, 2015) and decreases expression of two out of three collagen encoding genes (COL1A1, COL1A2, COL4A1) that are upregulated in liver cancer and NASH (Appendix Fig S1D). Therefore, we next tested effects of BTC on human hepatic stellate cells using the LX2 cell line (Xu *et al*, 2005). LX2 cells were grown and pretreated with and without BTC using EGF as a growth factor positive control. LX2 growth was significantly increased by BTC (Fig 4A) and to a similar extent as was observed for EGF (Appendix Fig S1E). Moreover, we observed increased collagen staining (Sirius red) in cells treated with BTC (Fig 4B).

We next performed a RNASeq transcriptomic analysis of LX2 cells treated with BTC and compared it to genes regulated by DHA *in vivo*. We found 63 genes upregulated by BTC and downregulated by DHA and 16 genes downregulated by BTC and upregulated by DHA. Among the enriched pathways for the set of genes induced by BTC and repressed by DHA were transcripts involved in cell growth including ERBB signaling (Fig 4C, Appendix Fig S1F–H, Dataset EV5). Strikingly, TGFB2 was the only gene found in common across several enriched categories. Moreover, expression of TGFB2, but not TGFB1 was increased by BTC *in vitro* (Fig 4D, Appendix Fig S1G), repressed by DHA in both prevention and treatment mouse studies (Fig 4E), and increased in human liver cancer meta-analysis (Fig 4F). A recent study demonstrated that TGFβ-2, but not TGFβ-1 has a critical non-redundant role in promoting lung and liver fibrosis (Sun *et al*, 2021). Therefore, we hypothesized that downstream effects of reduction of BTC by DHA can be explained by reduction of TGFβ-2. For this, using publicly available *in vitro* data on TGFβ-2 effects and our *in vivo* data, we evaluated which genes regulated by DHA were regulated in the opposite direction by TGFβ-2 (Dataset EV6). We found 62 genes upregulated by TGFβ-2 and downregulated by DHA and another 62 genes downregulated by TGFβ-2 and upregulated by DHA. DHA downregulated/TGFβ-2-upregulated genes were highly enriched for production of collagen trimers and extracellular matrix organization (Fig 4G)

Figure 4. Hepatic stellate cell (LX2) proliferation could promote fibrosis via BTC-TGFβ-2, reversed by DHA.

A LX2 cell proliferation assay after treatment with BTC, 0–25 ng/ml (left panel, $N = 6$ individual experiments). Fold change in DNA concentration after treatment with BTC 0–10 ng/ml (right panel, $N = 3$ experiments). Green bar in each plot is untreated control. Data are displayed as mean ± SD with each point being an individual experiment; Ordinary One-way ANOVA, multiple comparisons test with control vehicle, ns (not significant), $*P < 0.05$, $**P < 0.001$, $***P < 0.005$, $****P < 0.0001$).

B Collagen staining (Pico Sirius Red, PSR) in LX2 cells indicating increased fibrosis (collagen production) when stimulated with BTC (20 ng/ml; $N = 5$ experiments) or a vehicle control. A representative image of stained cells is shown (left panel). A bar plot of staining intensity normalized by total protein (μg) per well (Data are displayed as mean ± SEM, $N = 5$ experiments; unpaired, two-sided t-test $**P = 0.0058$).

C Gene enrichment (biological process) of LX2 cells (see Materials and Methods) while induced by BTC (BTC vs. Vehicle; one-sided *t*-test) identifies pathways reversed by DHA treatment *in vivo*. Data are displayed as $-\log_{10}$(*P*-value).

D Bar plot of TGFB2 gene expression in LX2 cells treated with vehicle or BTC (20 ng/ml; Data are displayed as mean ± SD, $N = 5$ experiments) *in vitro* (paired, two-sided *t*-test $*P < 0.05$, FDR < 0.1).

E Tgfb2 gene expression *in vivo*. DHA reversed the gene expression significantly in the *in vivo* experimental model both in Preventive & Treatment models (Data are displayed as mean ± SD, $N = 8$ mice/group [preventative study] or $N = 5$ or 6 mice per group [treatment study]; Ordinary One-way ANOVA, multiple comparisons test of reference diet [chow, RD], western diet + DHA [WD + DHA], western diet + EPA [WD + EPA] each with western diet + olive oil [WD + O], ns [not significant], $*P < 0.05$, $**P < 0.001$, $***P < 0.005$, $****P < 0.0001$).

F TGFB2 gene expression from meta-analysis of human normal and liver cancer data indicates a highly significant increase in expression in the liver cancer samples (Effect size FDR = 0.006). The size of each individual dot represents the number of patients associated with the dataset with higher the number of patients darker the dot color, Normal (green) and Cancer (red; 7 transcriptomic datasets with a total of 544 tumor, 260 non-tumor, and 32 healthy patient liver samples).

G Gene enrichment analysis of genes upregulated in TGFβ-2 treated cells (GSE45382) that were reversed by DHA *in vivo*, identifies top enriched pathways. Data are displayed as $-\log_{10}$(*P*-value).

H Gene enrichment analysis of downregulated genes in TGFβ-2 treated cells (GSE45382) that were reversed by DHA *in vivo*, identifies highly enriched gene ontologies. Data are displayed as $-\log_{10}$(*P*-value).

 © 2023 The Authors

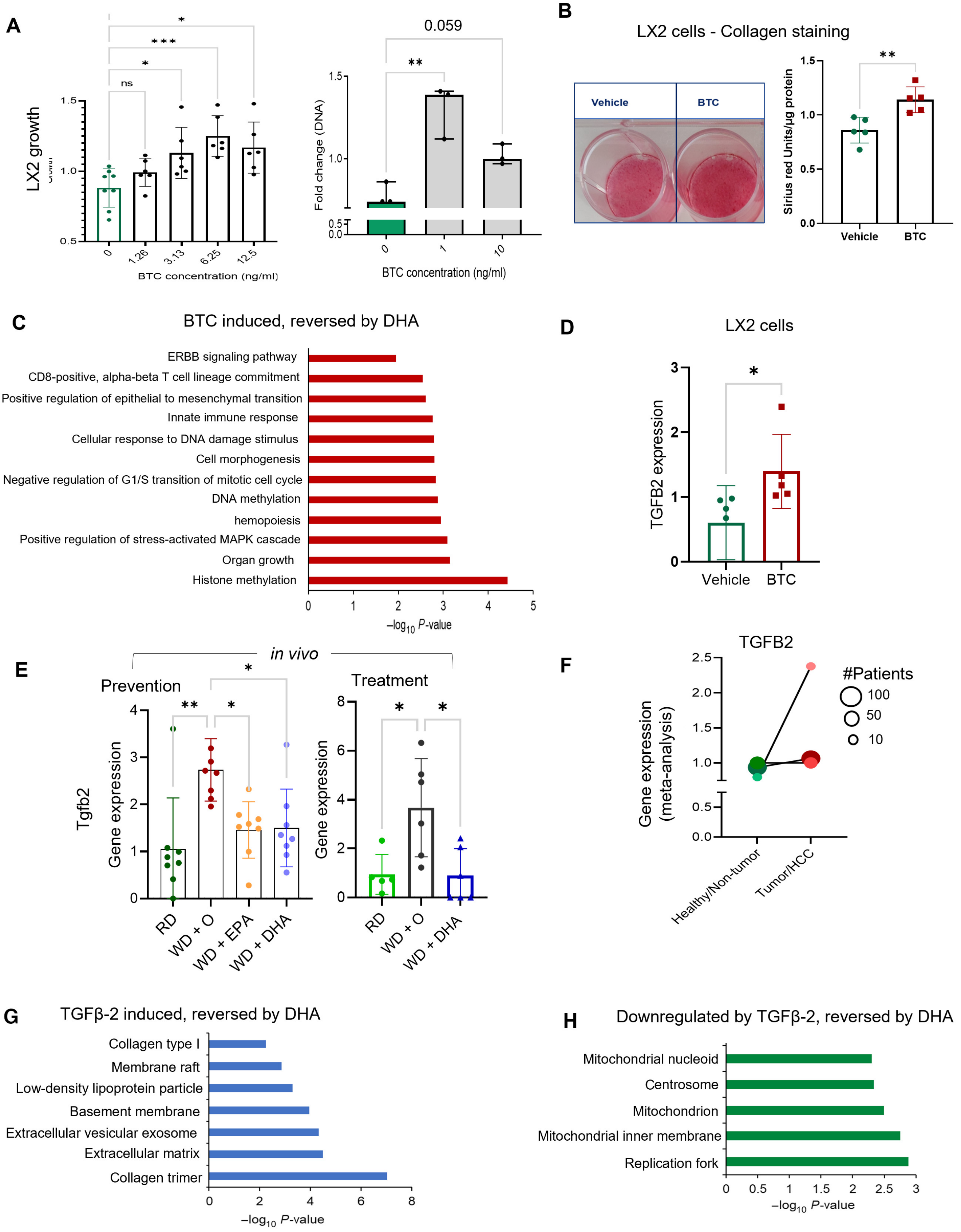
A
LX2 growth
BTC concentration (ng/ml)
Fold change (DNA)
0.059
BTC concentration (ng/ml)
B
LX2 cells - Collagen staining
Vehicle
BTC
Sirius red Units/µg protein
C
BTC induced, reversed by DHA
ERBB signaling pathway
CD8-positive, alpha-beta T cell lineage commitment
Positive regulation of epithelial to mesenchymal transition
Innate immune response
Cellular response to DNA damage stimulus
Cell morphogenesis
Negative regulation of G1/S transition of mitotic cell cycle
DNA methylation
hemopoiesis
Positive regulation of stress-activated MAPK cascade
Organ growth
Histone methylation
–log10 P-value
D
LX2 cells
TGFB2 expression
Vehicle
BTC
E
in vivo
Prevention
Treatment
Tgfb2
Gene expression
RD
WD + O
WD + EPA
WD + DHA
F
TGFB2
#Patients
Gene expression (meta-analysis)
Healthy/Non-tumor
Tumor/HCC
G
TGFβ-2 induced, reversed by DHA
Collagen type I
Membrane raft
Low-density lipoprotein particle
Basement membrane
Extracellular vesicular exosome
Extracellular matrix
Collagen trimer
–log10 P-value
H
Downregulated by TGFβ-2, reversed by DHA
Mitochondrial nucleoid
Centrosome
Mitochondrion
Mitochondrial inner membrane
Replication fork
–log10 P-value

Figure 4.

while genes downregulated by TGFβ-2 and reversed by DHA were enriched for mitochondrial inner membrane and other mitochondrially related functions (Fig 4H). Altogether these results suggest that one of a key mechanism of fibrosis reduction by DHA is achieved through an inhibition of BTC and a consequent reduction of HSCs proliferation and TGFβ-2-induced collagen production (Sun *et al*, 2021).

TLR-dependent inflammatory processes in NASH are exacerbated by BTC

Our results from stellate cells support that inhibition of BTC by DHA would explain reduction of fibrosis and improvement of mitochondrial function. However, the reduction in inflammatory pathways and macrophage gene expression, another major effect of DHA we observed (Figs 1 and 2), could not be explained by effects of BTC on stellate cells. Furthermore, EGFR deficiency specifically in macrophages has been shown to attenuate liver cancer in a mouse model (Lanaya *et al*, 2014). Finally, we have previously reported that systemic levels of TLR2 ligands are decreased by DHA (Lytle *et al*, 2015; Appendix Fig S2A). These results, along with a well-known fact that a genetic deficiency of microbiota sensors (TLR2 and TLR4) attenuates NASH (Spruss *et al*, 2009; Miura *et al*, 2013; Wu *et al*, 2020), indicate that a reduction in TLR2 and/or TLR4 ligand levels by DHA might interact with the reduction of BTC in preventing the disease. While different receptors for BTC are widely distributed across different liver cell types (Appendix Fig S1B and S2B), TLR2 and TLR4 are predominantly expressed by macrophages in the liver (Appendix Fig S2C).

Taken together, our next question was: which processes regulated by DHA in the liver can be explained by the effects of BTC and TLR2/4 agonists on macrophages? We also asked whether BTC modulates TLR2/4-dependent immune stimulation in macrophages. To answer these questions, we differentiated the human monocyte cell line (THP-1) to a macrophage-like phenotype and stimulated with BTC, LPS (TLR4 ligand) and PGN (TLR2 ligand) and compared global gene expression in these cells to cells stimulated with TLR2/4 ligands only or unstimulated control cells (Appendix Fig S2D, Dataset EV7).

To investigate a potential interaction effect, we tested a range of concentrations of BTC and TLR-agonists evaluating expression of IL-8, and CCL2 (MCP-1) expression, well-known targets of BTC (Lanaya *et al*, 2014; Shi *et al*, 2014) and TLR-agonists (Seki *et al*, 2007; Spruss *et al*, 2009; Miura *et al*, 2013; Wu *et al*, 2020) and chose the lowest doses that induces their expression (Appendix Fig S2E and F). We next performed transcriptomic analysis of THP-1 cells treated with BTC-LPS-PGN and compared it to genes regulated by DHA *in vivo*. We found 179 genes upregulated by BTC-LPS-PGN and downregulated by DHA and 285 genes downregulated by BTC-LPS-PGN and upregulated by DHA (Fig 5A). Genes upregulated by BTC-LPS-PGN were enriched for several pathways related to monocyte/macrophage related immune functions, the cell cycle, and collagen binding. Among the most enriched categories for the downregulated genes were mitochondrion, NAD metabolic process, and endoplasmic reticulum membrane (Fig 5B–G).

To assess the relative contribution of BTC and of TLR2/4 agonists to the observed combined functional effect, we calculated a summary metric for each pathway (see Materials and Methods) and compared their values between each treatment group (Fig 5D).

To check if the expected effects of BTC are present in macrophages, we first verified expression of EGFR/cell cycle and epithelial-mesenchymal transition (EMT) pathways and observed their increase among the categories upregulated by BTC alone and in combination with TLR agonists (Fig 5E). Analysis of the downregulated pathways showed that in combination with TLR ligands BTC inhibited expression of genes involved in mitochondrial functions (TCA cycle, oxidative phosphorylation, Appendix Fig S2G) and NAD metabolic functions (critical pathways operating in mitochondria; Samuel & Shulman, 2018; Simões *et al*, 2018; Xie *et al*, 2020; Fig 5F).

As for the upregulated pathways, we observed diverse immune functions such as ‘innate immune response’, ‘regulation of interferon-gamma signaling’ with a similar or slightly enhanced expression of genes when BTC was added with TLR2/4 agonists (Fig 5G, Appendix Fig S2H). However, some genes showed clear interactions between BTC and TLR-agonists. For example, IL1B was upregulated by TLR2/4 agonists and slightly increased by BTC, but CSF1, a classical factor of macrophage growth and proliferation

Figure 5. BTC promotes TLR-dependent inflammation and integrin production by macrophages.

A Scatterplot of differential expression with BTC-LPS-PGN (BTC and TLR2/4 ligands) treated cells to control THP-1 cells *in vitro* (BTC-LPS-PGN/control in THP-1 cells; P-value < 0.05; Details about concentrations and duration of the TLR agonists treatment are detailed in Materials and Methods) against *in vivo* differential expression with WDO to WD + DHA in NASH preventive model. Filled circles are the genes in DHA treatment category.

B Gene enrichment is shown for the genes upregulated from BTC-LPS-PGN treatment in THP-1 cells that were reversed by DHA treatment *in vivo* (Gene ontology-biological process). Data are displayed as $-\log_{10}$(P-value).

C Gene enrichment is shown for the genes downregulated from BTC-LPS-PGN treatment in THP-1 cells that were reversed by DHA treatment *in vivo* (Gene ontology-biological process and cellular components). Data are displayed as $-\log_{10}$(P-value).

D The heatmap for summary metric of all major molecular pathways affected by BTC treatment in THP-1 cells shown to be prevented by DHA in both *in vivo* NASH preventive and treatment models (see Materials and Methods).

E–G The individual pathways enriched in BTC-LPS-PGN treated THP-1 cells but reversed by DHA *in vivo* in the NASH preventive and treatment models displayed as summary metric (Data are displayed as mean ± SD, $N = 5$ experiments).

H Individual gene expression for IL-1b and CSF1 from THP-1 cells *in vitro* treated with BTC and or TLR2/4 ligands ($N = 5$ experiments, paired, one-sided t-test, ns (not significant), *$P < 0.05$, **$P < 0.001$; see Materials and Methods) and *in vivo* NASH preventive model (Data are mean ± SD, $N = 8$ mice/treatment group; Ordinary One-way ANOVA, with Dunnett's multiple comparisons test with WD + O, ns (not significant), *$P < 0.05$, **$P < 0.001$, ***$P < 0.005$, ****$P < 0.0001$).

I The bar plots for summary metric are shown for integrin signaling and collagen pathway in THP1 cells (Data are displayed as mean ± SD, $N = 5$ experiments).

J The bar plot for ITGA1 gene expression is shown in THP-1 cells *in vitro* treated with BTC and or TLR2/4 ligands ($N = 5$ experiments, paired, one-sided t-test, ns (not significant), *$P < 0.05$, **$P < 0.001$; see Materials and Methods) and *in vivo* NASH preventive model (Data are mean ± SD, $N = 8$ mice/treatment group; Ordinary One-way ANOVA, with Dunnett's multiple comparisons test with WD + O, *$P < 0.05$, ****$P < 0.0001$).

 © 2023 The Authors

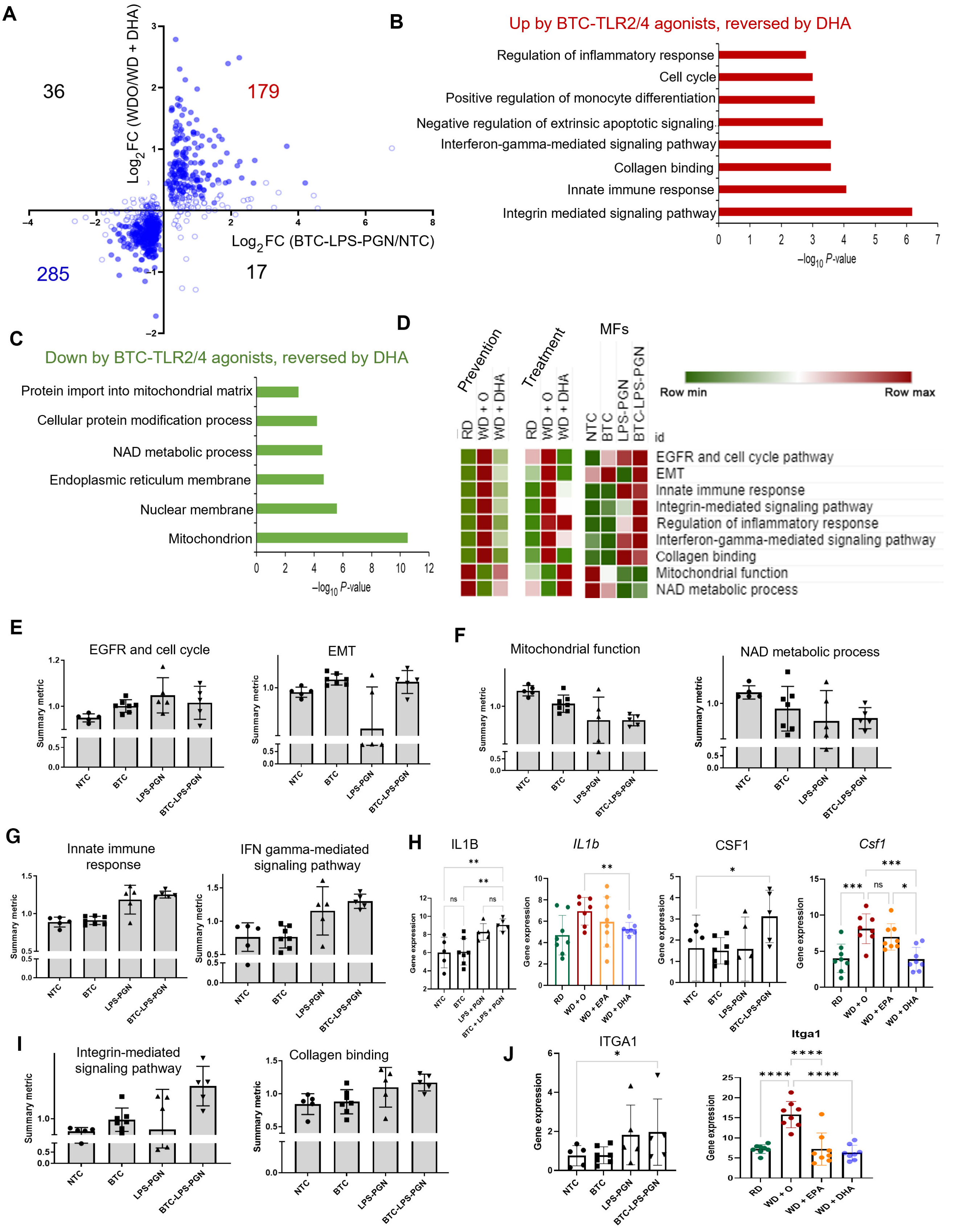
A
36
179
285
17
Log2FC (WDO/WD + DHA)
Log2FC (BTC-LPS-PGN/NTC)
B
Up by BTC-TLR2/4 agonists, reversed by DHA
Regulation of inflammatory response
Cell cycle
Positive regulation of monocyte differentiation
Negative regulation of extrinsic apoptotic signaling
Interferon-gamma-mediated signaling pathway
Collagen binding
Innate immune response
Integrin mediated signaling pathway
−log10 P-value
C
Down by BTC-TLR2/4 agonists, reversed by DHA
Protein import into mitochondrial matrix
Cellular protein modification process
NAD metabolic process
Endoplasmic reticulum membrane
Nuclear membrane
Mitochondrion
−log10 P-value
D
Prevention
Treatment
MFs
RD
WD + O
WD + DHA
NTC
BTC
LPS-PGN
BTC-LPS-PGN
Row min
Row max
id
EGFR and cell cycle pathway
EMT
Innate immune response
Integrin-mediated signaling pathway
Regulation of inflammatory response
Interferon-gamma-mediated signaling pathway
Collagen binding
Mitochondrial function
NAD metabolic process
E
EGFR and cell cycle
EMT
F
Mitochondrial function
NAD metabolic process
G
Innate immune response
IFN gamma-mediated signaling pathway
H
IL1B
IL1b
CSF1
Csf1
I
Integrin-mediated signaling pathway
Collagen binding
J
ITGA1
Itga1
Summary metric
Gene expression

Figure 5.

(Hume & MacDonald, 2012), was upregulated only when both BTC and TLR2/4- agonists were present (Fig 5H).

Among immune-related pathways, the strongest BTC effect either alone or in combination with TLR2/4 agonists was on the integrin-mediated signaling pathway, which partially overlapped with collagen binding (Fig 5I). Interestingly, while there was a trend for BTC increasing transcript levels of ITGα6 and ITGα9 and TLR2/4 agonists of ITGα1, only the combination of BTC with TLR-agonists significantly induced expression of all three integrins (Fig 5J, Appendix Fig S2I). Notably, the integrin pathway was not regulated by TLR2/4 agonists alone suggesting a possible unique role of the crosstalk between EGFR and TLR pathways in controlling fibrosis-related molecular function in macrophages.

Potential mechanism of BTC regulation by ω3 fatty acids

As a last step of this study, we sought to identify potential upstream regulators (transcription factors, TF) that could be targets of ω3 PUFA in control of betacellulin and its downstream processes. For this, we first established BTC-downstream profile by searching in the network genes that were regulated by BTC *in vitro* (Figs 4C and 5B–D) and by ω3 PUFA in the NASH mouse model (Fig 2A) in the opposite directions. We found 150 genes (62 up and 88 down regulated by BTC) and defined them as ω3-controlled BTC-regulated genes. Next, we investigated which genes (and which transcription factors among them) might mediate effects of ω3 PUFA on BTC-regulated genes. First, we ranked genes using the network degree and BiBC between ω3 PUFA and BTC-regulated genes (Fig EV5A). Among the 41 top-ranked genes, we found three transcription factors with two of them (Foxo3 and Kdm5d) expressed in cholangiocytes (the only expressing BTC in the liver). Further investigation of binding motifs identified that only Foxo3 had a motif in the Btc promoter (Fig EV5B), suggesting that regulation of this TF by ω3 PUFA can be implicated in the regulation of BTC. Interestingly, Foxo3 is a well-known suppressor of oncogenesis (Liu *et al*, 2018; Tsuji *et al*, 2021), and we found that this TF is downregulated by WD and upregulated by ω3 PUFA in mice (Fig EV5C).

Discussion

For many metabolic diseases such as diabetes (Jermendy *et al*, 2018; Dahlén *et al*, 2021), atherosclerosis (Bhatt *et al*, 2019), and obesity (Jastreboff *et al*, 2022) there are highly efficient drugs that treat and/or prevent development of these diseases. The only frequent representative of metabolic diseases that still lack pharmacological agents that would pass clinical trials is NAFLD/NASH. As this disease is often called "fatty liver", most of attempted treatments target reduction of liver fat (Rinella, 2015). However, these treatments cannot resolve liver fibrosis, which is a more resistant aspect of the pathogenesis of this disease (Wattacheril *et al*, 2018). Fibrosis is the main cause of liver failure in patients with NASH and also precedes and leads to development of liver cancer (Anstee *et al*, 2019; Pfister *et al*, 2021). Therefore, revealing the mechanisms of action of DHA that reduce fibrosis in a preclinical mouse model may aid in the rational use of ω3 PUFA or help in developing new drugs that would act upon the same cellular/molecular targets (Jump *et al*, 2015).

In this study, we identified betacellulin (BTC), one of the less studied ligands of EGFR, as a master regulator whose reduction by DHA potentially leads to prevention/treatment of fibrosis. Indeed, we revealed that BTC induces the TGFβ-2, a critical contributor to liver fibrosis via collagen production by hepatic stellate cells (Dropmann *et al*, 2016). Moreover, in combination with TLR 2/4-agonists (also reduced by DHA), BTC induces integrin pathway in macrophages, the cell type in the liver most affected by DHA treatment and well-known to be involved in pathogenesis of fibrosis in different organs (Wynn & Vannella, 2016). Thus, BTC represents a candidate master regulator inducing two most important factors (collagens and integrins) contributing to liver fibrosis and consequently promoting liver cancer.

In addition to its effect on fibrosis, reduction of BTC seems to be also mediating another important effect of DHA, which is improvement of mitochondrial function-related pathways.

Indeed, mitochondrial damage is widely reported in NAFLD/NASH and its improvement by DHA is clearly seen even at the initial stage of our analysis (Fig 1E) indicating a strong impact of DHA on this pathway. Accordingly, BTC in combination with microbiota derived stimulation (represented by TLR agonists) has a negative effect on the expression of genes involved in mitochondrial functions.

Another robust effect of DHA observed at the initial step of our analysis was the inhibition of inflammation. Not surprisingly, a deeper investigation into this phenomenon led us to potential effects of DHA on microbiota-related molecules and on hepatic macrophages involved in sensing microbes. In fact, network analysis combined with scRNAseq data pointed to macrophages as main cellular targets of DHA in the liver (Fig 2B). Macrophages are also the primary cells in the liver that express both TLR 2/4 (Appendix Fig S2C) whose microbiota-derived agonists are decreased by DHA (Appendix Fig S2A; Lytle *et al*, 2015). This was an important observation considering that deficiency of either TLR2 or TLR4 attenuates severity of NADFL/NASH in mouse models (Spruss *et al*, 2009; Miura *et al*, 2013; Wu *et al*, 2020).

In contrast to TLRs, EGFR and other receptors that BTC binds are widely expressed across most cells in liver including macrophages. Moreover, EGFR deficiency in macrophages, but not in hepatocytes was shown to attenuate NASH in mice (Lanaya *et al*, 2014).

Thus, it is plausible that the impact of DHA on macrophage related to NAFLD/NASH pathogenesis can be explained by the fact that DHA simultaneously limits cell access to BTC and TLR2/4 agonists. Moreover, despite the limitations of our cell culture system and the difficulty of transition from mice to humans, we observed that BTC combined with TLR 2/4 agonists induce the integrin signaling pathway which was inhibited by DHA in the liver. Inspecting individual genes revealed that all detected integrins (ITGα1,6,9—Fig 5J, Appendix Fig S2I) required all three compounds for induction except for ITGβ1 that increased only with only BTC stimulation in THP-1 cells. Interestingly, ITGβ1 was also increased by BTC in hepatic stellate cells (Appendix Fig S1H). Of note, proteins coded to ITGαs and ITGβs form a complex that binds collagens (Bourgot *et al*, 2020). Accordingly, blocking integrin signals has been shown to attenuate fibrosis (Agarwal, 2014; Rahman *et al*, 2022). Hence, our results taken together with already existing literature about other pathologies (Bourgot *et al*, 2020) suggest that DHA inhibits the macrophage contribution to fibrosis by simultaneously

© 2023 The Authors

inhibiting microbiota-derived signals and BTC. Furthermore, while BTC is a new player in this arena, the role of microbiota-derived signals in activating a profibrotic program in macrophages has been reported for different diseases in a few organs (He *et al*, 2021; Costa *et al*, 2022).

Leveraging complex multi-omic and single cell data (Xiong *et al*, 2019) and using a systems approach, our study constructed a statistical network model of cell–cell interactions affected by DHA (Fig EV4A). More importantly, network cause–effect related information flow (degree and BiBC) combined with a ligand–receptor database (Abugessaisa *et al*, 2021; Fig EV4A) allowed us to infer that a candidate master regulator molecule (BTC) produced by one cell (cholangiocytes) acts upon several other cells. DHA may affect several cell types involved in liver fibrosis. Our study, however, reveals that BTC inhibition by DHA simultaneously disrupts the integrin pathway in macrophages and TGFβ-2-driven collagen production by hepatic stellate cells, two processes that synergize in the development of liver fibrosis. Thus, we propose that removal of BTC and Tlr2/4 agonists prevents binding of integrins to collagen that is required for the scar development.

The main outcomes of our study, however, might be missing additional beneficial effects of DHA, especially those that are not related to BTC reduction and fibrosis. This is because we focused our investigations on cellular/molecular events relevant to NASH and its progression to liver cancer in humans. Specifically, while our network analysis modeled NASH in mice, we used it as a first step in identification of the most critical causal pathways upregulated in hepatic cancer meta-analysis and reversed by DHA. In this analysis we found ERBB as a top pathway with BTC being the top-ranked molecule in this pathway altered by DHA. Accordingly, our *in vitro* investigations of human hepatic stellate cells demonstrated that BTC promotes cell growth, which was an expected finding considering that BTC belongs to a family of growth factors (Olayioye *et al*, 2000; Wieduwilt & Moasser, 2008; Chen *et al*, 2016). Thus, in addition to promoting fibrosis by upregulation of TGFB2 in stellate cells, it may also increase numbers of these collagen-producing cells in the liver. Furthermore, our gene expression analyses of effects promoted by BTC *in vitro* and downregulated by DHA in the liver also support this growth function for BTC (Fig 4A), pointing to an activation of ERBB pathways, cell growth and even the epithelial mesenchymal transition. Dysregulation of these pathways are hallmarks of cancer (Dahlhoff *et al*, 2014; Lanaya *et al*, 2014; Chava *et al*, 2022). The growth-promoting actions of BTC are known to be mediated by epidermal growth factor receptors (ERBBs), namely ERBB1 (EGFR; Chava *et al*, 2022), ERBB2, ERBB3, and ERBB4. In liver however, the mechanism for BTC dependent cell proliferation has not been elucidated. The BTC-EGFR-ERBB4 pathway in pancreatic ductal adenocarcinoma has been well established by several groups and a BTC knock out can partially rescue the cancer progression (Hedegger *et al*, 2020). In the *in vivo* NASH model, we have a significant reversal of ERBB4 expression by DHA (Appendix Fig S2B). Furthermore, DHA also inhibits GRB2 and GSK3β, genes which are downstream in the ERBB signaling pathway found in our liver cancer meta-analysis (Fig 3A and C) and known to promote multiple types of cancers (He *et al*, 2021). These results suggest that DHA, besides its main effect as BTC reduction, might also inhibit residual activation of ERBB pathway via BTC-unrelated mechanisms. In addition, 49 EMT related genes, including the TGFβ-2 mediated (Dropmann *et al*, 2016) SMAD-EMT-cancer pathway were reversed by DHA treatments (Fig 3A). Therefore, our results suggest that the DHA attenuation of BTC-TGFβ-2-dependent molecular events might not be limited to reversal of fibrosis in NASH (Lytle *et al*, 2015) but also has a promise in preventing NASH's progression into liver cancer.

While investigating regulatory events upstream of BTC, we found FOXO3, a transcription factor with well-established function of tumor suppression (Liu *et al*, 2018; Tsuji *et al*, 2021), as a probable mediator of inhibitory effects of omega 3 fatty acids on BTC. These results provide additional details for understanding the molecular mechanism omega 3 action in the liver and contribute to rationale to use them for liver cancer prevention.

Clinical trials using EPA and/or DHA in NAFLD/NASH therapy have shown mixed, but promising results with significant improvement with disease severity, including hepatosteatosis and liver injury (Hodson *et al*, 2017; Zöhrer *et al*, 2017; Musa-Veloso *et al*, 2018; Okada *et al*, 2018; Tobin *et al*, 2018; Šmíd *et al*, 2022). Unfortunately, these studies did not address whether patients that did not respond to therapy represent a cellular/molecular subtype of NAFLD that is not treatable only by ω3 PUFA or perhaps have such severe disease that they are unlikely to respond to any therapeutic agent.

Despite the novelty and importance of our findings several questions remain to be investigated. For example, to evaluate the relative contribution of down regulation of BTC by DHA for treatment of NASH and prevention of cancer, experiments using mouse models are warranted.

In conclusion, with the discovery of BTC as a candidate to be one of the key mediators of ω3 PUFA therapeutic effects, our study opens a new avenue for investigation of NAFLD/NASH. In addition to finding new mechanisms of action of DHA, this study is the first to demonstrate that BTC can induce TGFβ-2 and synergize with microbial signals in the induction of integrins. Thus, while few earlier studies (Moon *et al*, 2006) showed increase of BTC in the liver tumors, our robust meta-analysis coupled with evidence for causal contributions shed a new light to this molecule in the pathogenesis of this cancer. Moreover, BTC's role in human NAFLD/NASH is entirely uncharted territory. Therefore, future studies should investigate if BTC-triggered gene expression signatures can serve as biomarkers guiding personalized ω3 PUFA therapy, as targets of new NAFLD/NASH drugs, and finally as a predictors of hepatic cancer risk in humans.

Materials and Methods

Animals and diets

Study design for DHA mediated NASH prevention and remission in Male *Ldlr*$^{-/-}$ mice.

This study was carried out in strict accordance with the recommendations in the Guide for Care and Use of Laboratory animals of the National Institutes of Health. All procedures for the use and care of animals for laboratory research were approved by the Institutional Animal Care and Use Committee at Oregon state University (Permit number: A3229-01). Anthropometric, plasma and liver samples used in this study were obtained from our previously published

NASH prevention and treatment studies (Depner *et al*, 2013a; Lytle *et al*, 2015). Briefly, male mice (B6:129S7-*Ldlr*$^{tmHer/j}$, stock# 002207) were purchased from Jackson Labs and were group housed (4 mice/cage; $n = 8$ mice per group) and maintained on a 12-h light/dark cycle. Mice were acclimatized to the Oregon State University Linus Pauling science center vivarium for 2 weeks before proceeding with the experiments.

NASH prevention study

This study was designed to determine if EPA and DHA differed in their capacity to prevent western diet-induced NASH (Depner *et al*, 2013a). Mice consumed the one of the following 5 diets, *ad libitum* for 16 weeks; each group consisted of 8 male mice. Purina chow 5001 consisting of 13.5% energy as fat and 58.0% energy as carbohydrates was used as the Reference diet (RD). The western diet (WD) was obtained from Research Diets (12709B) and used to induce NASH. The WD consisted of 17% energy as protein, 43% energy as carbohydrate, and 41% energy as fat; cholesterol was at 0.2% wt:wt and does not contain either EPA or DHA (Depner *et al*, 2013a). The WD was supplemented with olive oil (WDO), eicosapentaenoic acid (WD + EPA), docosahexaenoic acid (WD + DHA), or both EPA and EHA (WD + EPA + DHA). EPA and DHA were added to the diets to yield 2% of total calories; for the EPA + DHA, each was added to yield 1% of total calories, i.e. 2% total calories as $C_{20\text{-}22}$ ω3 PUFA. Olive oil was added to the WD to have a uniform level of fat energy in all the WDs. Preliminary studies established that the addition of Olive oil had no effect on diet-induced fatty liver disease in *Ldlr*$^{-/-}$ mice. EPA was purchased from Futurebiotics as Newharvest EPA, a DuPont product, while DHA was obtained as a generous gift from DSM, formally Martek Bioscience). The amount of EPA or DHA added to the diets is equivalent to the amount prescribed for treating patients for hypertriglyceridemia (Harris *et al*, 1997; Davidson *et al*, 2007). At the end of the 16-week feeding trial, mice were euthanized with CO_2, blood (RBC and plasma) and liver were collected and stored at −80°C for later extractions of RNA, lipids, proteins.

NASH treatment study

This study was designed to assess the capacity of DHA to reverse the effects of WD-induced NASH (Lytle *et al*, 2017). As such, male *Ldlr*$^{-/-}$ mice were fed the WD for 22 weeks. These mice were separated into two groups: one group was maintained on a WD + olive oil, with the other group was maintained on the WD + DHA. The diet composition was as described above for the prevention study. Both groups were euthanized after 8 weeks of these diets. A control group was maintained on the RD for 30 weeks. At the end of the study, mice were euthanized, and samples collected and processed as above.

Liver histology

Approximately 100 mg of fresh mouse liver from each animal was fixed in buffered formalin, paraffin embedded, sliced and stained with hematoxylin–eosin (H & E), trichrome or Picro Sirius red (PCR; Nationwide Histology, Veradale, WA). Each slide contained 2–4 liver slices. Histological analysis and scoring for microsteatosis and macrosteatosis, inflammation (leukocytes) and fibrosis were provided by two investigators using the modified Kleiner scoring system established for mouse models of NAFLD as described previously (Garcia-Jaramillo *et al*, 2019). Histological samples were blinded as to diet, timepoint and diet. Digital images were taken with a Nikon Eclipse 6 microscope and digital camera (Mpixel) and NIS-BR Elements imaging software (v21.1; www.nikonmetrology.com). Digital images taken at 400× magnification were used for steatosis scoring. An effort was made to place the central vein of a lobule at one of the corners of the image so that each image covered at least one-quarter of that lobule, including all three zones of the hepatic lobule. Steatosis was objectively analyzed as the average percent surface area occupied by vacuoles using the image analysis software, ImageJ (https://imagej.nih.gov/ij/). Two images were taken of H&E-stained sections at 400× and the percent of affected surface area was calculated for each. The two values were then averaged. Steatosis was subjectively analyzed as the percent of affected surface area observed on H&E-stained slides at 100× (10× objective) and 400× magnifications. Vacuolization was characterized as macro vesicular, in which vacuoles displace hepatocyte nuclei, or micro vesicular. Macro vesicular and micro vesicular steatosis were scored separately. Severity was scored using the scale: 0 (0%), 1 (> 5% but < 33%), 2 (> 33% but < 66%), 3 (> 66%). When possible, the distribution of vacuoles was described as pericentral, midzonal, or periportal. Inflammation was defined as intralobular inflammatory foci of at least 5 leukocytes associated with disruption of hepatic plates or increased hepatocellular eosinophilia. Inflammation scores were calculated as the total number of clusters averaged over 5 fields in H&E-stained tissues examined at 100× (total number of clusters in 3.1 mm^2). The following scale was used: normal 0 (< 0.5 foci), slight 1 (< 0.5–1 foci), moderate 2 (1–2 foci), severe 3 (> 2 foci). Fibrosis was also objectively quantified as percent surface area occupied by Sirius Red-stained collagen by image analysis using ImageJ. Two images were taken at 100× from the liver section of each mouse and the calculated percentage areas were averaged. Fibrosis was subjectively analyzed to determine severity and distribution patterns, perisinusoidal, periportal, pericentral, or bridging. The following scale was used: absent (0), mild (1), moderate (2), or severe (3).

Cell culture

LX2 cells were obtained from SL Friedman (Mount Sinai Medical School; Xu *et al*, 2005). LX2 cells are activated human hepatic stellate cells; they were maintained in DMEM with 10% FCS containing penicillin and streptomycin. Cells were plated onto 100 mm plastic petri dishes ~ 100,000 cells/plate and treated with fatty acids (at 50 μM) in endotoxin-free BSA (at 20 μM) during the growth phase. Fatty acids [oleic acid (18:1 ω9) and DHA (22:6, ω3); Nu-Chek Prep] were used at 50 μM for 2–48-h cycle treatments. After this pre-treatment, cells were trypsinized, washed in PBS, counted and plated at 3,000 cells/well in a 96-well plate. Cells were fed DMEM with 1% FCS without or with betacellulin (human recombinant betacellulin [BTC], R&D Systems) for 72 h; the concentration ranged from 1.25 to 25 ng/ml. At the completion of BTC treatment, media was removed, washed with PBS and DNA/well was quantified using the CyQuant cell proliferation assay (Thermofisher); DNA fluoresces was quantified (excitation at 485 nm & emission at 530 nm). This experiment was repeated 3 times.

© 2023 The Authors

Collagen production

Pico Sirius red quantitation of collagen production: Cells were pretreated with fatty acids as described above, trypsinized, counted and plated onto 12-well cell culture plates at 80,000 cells/well in DMEM +1% FCS and treated without and with 20 μg/ml BTC for 72 h. At the end of treatment, media was removed, cells were washed with PBS and stained with pico Sirius Red (Abcam) for 2 h at room temperature. After staining cells were washed with an acetic acid solution, photographed and the stain was removed using 50 mM NaOH. The level of staining per well was quantified at 540 nm. The level of protein/well was quantified after solubilizing the protein in 50 mM NaOH and using the Pierce BSA kit. This experiment was repeated 4 times.

Cell viability

Alamar Blue (Creative Labs) assessment of cell vitality (NADH conversion to NAD^+): Using the protocol described above, we assessed the vitality of cells after treatment with fatty acids and BTC. Alamar blue (50 μl/1.0 ml media) was added to the cells and fluorescence was measured at 590 nm 4 h after Alamar Blue addition.

RNASeq

RNA was extracted from LX2 cells using Trizol (Invitrogen) as previously described (Lytle *et al*, 2015) from cells treated with fatty acids (oleic acid and DHA at 50 μM for 72 h) as described above. Cells were seeded onto 6-well plates at 40,000 cells/well; and cells were treated with fatty acids for 96 h as described above. Afterward, media was removed, and cells were treated without and with BTC at 20 ng/ml for 72 h. Cells were harvested for RNA extraction (Trizol). cDNA was prepared for RNASeq analysis as described.

THP-1 cells

BTC and TLR co-stimulation

THP-1 monocytes were cultured in RPMI 1640 medium adjusted to contain 4.5 g/l glucose and supplemented with 1% Penicillin/Streptomycin, 10% FBS, 1 mM sodium pyruvate, 10 mM HEPES. For experiments, monocytes were first seeded in 24 well plates at 400,000 cells/well in 1 ml of complete medium with 50 ng/ml PMA (phorbol 12-myristate 13-acetate) to induce polarization, for 24 h. Then, attached cells were washed with sterile PBS to remove residual serum and PMA containing media. Cells were stimulated with a TLR2 agonist at 40 ng/ml (PGN-BS, Invivogen, San Diego, CA), TLR4 agonist at 4 ng/ml (LPS-B5, Invivogen, San Diego, CA), BTC at 40 ng/ml (human recombinant Betacellulin protein, R&D Systems, MN), or combinations of all for 6 h. Treatments were prepared in serum-free semi-complete RPMI 1640 media (see above for other components) supplemented with 20 μM BSA and 50 μM BHT (Butylated hydroxytoluene). Cells were lysed with 300 μl RLT buffer (Qiagen) and cell lysates were stored at −80°C.

Primers

qRT–PCR data analysis

THP-1 cells' response to TLR and BTC stimulation was assessed by qRT–PCR (Appendix Table S1). Briefly, raw Cycle Threshold (CT) values from the StepOnePlus Real Time PCR instrument for genes of interest were normalized to C_T values of a housekeeping gene, TMEM59, by delta C_T method and relativized by $2^{-\Delta C_T}$. Data were then median normalized and Log_2 transformed before being plotted in GraphPad Prism 9.

RNA extraction and RNA sequencing library preparation

RNA was extracted from cell lysates using the RNeasy Mini Kit and subjected to a DNase treatment according to manufacturer's protocols (Qiagen) then stored at −80°C until further use. mRNA libraries were prepared for sequencing with the Lexogen QuantSeq 3′ mRNA-Seq Library Prep Kit (FWD) HT for Illumina Sequencing platforms (Kit#k15.384) and sequenced on the Illumina NextSeq at Oregon State University.

cDNA and qRT–PCR

cDNA was prepared from 0.5 to 1 μg of RNA via reverse transcription using the qScript XLT cDNA SuperMix kit (Quantabio). qRT–PCR was performed for gene expression using the AzuraView GreenFast qPCR Blue Mix HR (Azura Genomics). 96-well plates were prepared with 10 ng of cDNA in triplicate reactions for each gene and sample and run on an Applied Biosystems StepOnePlus Real Time PCR instrument.

Sequencing of RNA (RNAseq)

RNA libraries were prepared with the QuantSeq 3′mRNA-Seq Library Prep Kit (Lexogen) for the Apollo 324 NGS Library Prep System and sequenced using Illumina NextSeq. The sequences were processed to remove the adapter, polyA and low-quality bases by BBTools (https://jgi.doe.gov/data-and-tools/bbtools/) using bbduk parameters of k = 13, ktrim = r, forcetrimleft = 12, useshortkmers = t, mink = 5, qtrim = r, trimq = 15, minlength = 20. Then the reads were aligned to mouse genome and transcriptome (ENSEMBL NCBIM37) using Tophat (v2.1.1) with default parameters. The number of reads per million for mouse liver genes were counted using HTSeq (v 0.6.0) and quantile normalized. Cell lines sequencing/analysis was performed similarly, but with the bbduk parameters of k = 13, ktrim = r, forcetrimleft = 11, useshortkmers = t, mink = 5, qtrim = r, trimq = 10, minlength = 20. The reads were aligned to the human genome and transcriptome (Gencode v40) using STAR v2.5.3a. BRB-ArrayTools was used to identify differentially expressed genes between treatments.

Metabolomes and lipidomes

Data were prepared, and analysis was carried out from the NASH Prevention study and Treatment study. Hepatic lipids and non-lipid metabolites were extracted and subject to UPLC/MS/MS analysis as previously described (Garcia-Jaramillo *et al*, 2019; Rodrigues *et al*, 2021) with minor modifications. Processed normalized data are available in Dataset EV8.

Treatment categorization of omics data

The genes and other parameters whose expressions or values are significantly changed by WD (FDR < 10%) were considered for treatment effects. Out of these parameters and genes, those that have treatment effect reversal with a P-value < 0.05 were categorized as DHA if uniquely DHA (opposite to WD + O/ND & WD + DHA/WD + O P-value < 0.05), uniquely EPA (opposite to WD + O/ND & WD + EPA/WD + O P-value < 0.05), similar in both EPA&DHA (opposite to WD + O/ND & both WD + EPA/WD + O and WD + DHA/WD + O, P-value < 0.05) and with no

© 2023 The Authors

treatment effect (NA; with both WD + EPA/WD + O and WD + DHA/WD + O, P-value > 0.05). To be consistent with many previous studies, the treatment effects were also tested in the combination of preventive treatment though not used further in the analysis (EPA + DHA; WD + EPA + DHA/WD + O, P-value < 0.05).

Reconstructing the NASH liver multi-omic network

The network reconstruction was carried out as described in the previous papers from our group with minor dataset specific modifications (Dong *et al*, 2015, Li *et al*, 2022). The genes and other parameters whose expressions or values are significantly changed by WD (FDR < 10%) were chosen for constructing the NASH network. First, from liver tissue between all pairs of genes (GE) and metabolic parameters (phenotypes, P) spearman rank correlations were calculated by pooling the samples per diet (WD + O, WD + EPA and WD + DHA). Meta-analysis was performed to retain edges with same sign of correlation coefficient in all three diets. Edges were further filtered by the following criteria: individual P-value of correlation within each diet from pooled experiments < 20%, combined Fisher's P-value over diets from pooled experiments < 5% and FDR cutoff of 10% for edges within tissues and for phenotypes and between lipidomic, metabolomic and plasma biochemicals and edges needed to satisfy principles of causality (i.e., satisfied fold change relationship between the two partners in the WD + O vs. ND comparison). Next, correlations were calculated per diet for the experiment pairwise between parameters (Gene Expression + Lipidomic and Metabolomic data [LM]) and (P + LM). Finally, edges obtained from pooling were retained if they had the same sign of correlation coefficient as in 3 groups (3 diets, WD + O, WD + EPA and WD + DHA). False positive edges were removed (Yambartsev *et al*, 2016) while pooling the different diets in the creation of the network. The proportion of genes, metabolites and lipids that made it to the final network (following statistical cutoffs) was determined after applying selection for significance in differentially expressed parameters in liver prior to applying correlation cutoffs.

Single cell RNA (scRNA) sequencing data analysis

Datasets

Single cell dataset (GSE129516) for mouse NASH model was obtained from single cell RNA-sequence on non-parenchymal cells of healthy vs. NASH mouse liver. These are then reanalyzed and used in our multi-omic network analysis to infer liver cell types. Human liver single cell RNA sequencing data (GSE136103) from normal and Cirrhosis patients were reanalyzed (Ramachandran *et al*, 2019; Xiong *et al*, 2019).

scRNA sequencing analysis

The raw gene expression matrix (UMI counts per gene per cell in the liver tissue) was filtered, normalized, and clustered using a standard Seurat version 3.1.0 in R [https://www.R-project.org/] (Stuart *et al*, 2019). Cell and gene filtering were performed as mentioned in previous publications. During quality filtering, cells with a very small library size (< 5,000) and a very high (> 12%) mitochondrial genome transcripts were removed. The genes detected (UMI count > 0) in less than three cells were also removed from further processes. Then log normalization and further clustering is performed using standard Seurat package procedures. Principal component analysis was used to reduce dimensions that represent cell clusters. The number of components from this analysis used for the elbow of a scree plot, which further aid in selecting the significant clusters. The different cell type clusters in a sample were visualized using t-distributed Stochastic Neighbor Embedding of the principal components as implemented in Seurat. The liver tissue specific cell-type identities for each cluster were determined manually using a compiled panel of available immune and other cell specific marker expression as per the previously published papers (Ramachandran *et al*, 2019; Xiong *et al*, 2019).

Single cell RNA sequence for assignment of gene in the NASH network to a specific cell type

The normalized UMI > 1.0, with a fold change significantly and uniquely expressed genes in a cell specific cluster were then assigned to that specific set of genes in the network to indicate they belong to that specific cell type. It is the primary rule to assign a gene to a cell type. Next, higher expression in the cell cluster (and optional fold change ($\log_2$FC > 0.25, P value < 0.05) for a gene gets assigned with that specific cell type of the tissue. Here, additionally, ranking by average expression for each gene in the clusters helps to determine its cluster specificity by the higher expression in that cell type than another in the whole tissue. This is implemented for evaluating the highest cell cluster average expression of a gene, among all other cell clusters in network.

Detecting subnetworks and functional enrichment

Infomap (mapequation.org) was used to identify subnetworks using the default commands. Functional enrichment of clusters was then performed using metascape (http://metascape.org; Zhou *et al*, 2019).

Additionally single cell data overlay on NASH network as mentioned above allowed the cell type specific gene sub clusters.

Identifying of key nodes between subnetworks using BiBC analysis

Analysis of networks was performed using the python package NetworkX v2.2. Bipartite betweenness centrality (BiBC) was calculated between all cell cluster pairs (66 in total) of the 12 clusters previously identified based on single cell data overlay on NASH network. The nodes were then ranked by their resulting BiBC and scaled to range of 0–100. BiBC was also calculated and scaled similarly pairwise between all genes and anthropometric data, between all genes and Lipidomic/Metabolomic data.

Creation and analysis of random networks

Random networks were created similar to as was described in Kahalehili *et al* (2020). Briefly, 5,000 Erdos-Renyi random networks were created, utilizing the same number of nodes and edges that were present in the real, reconstructed network. BiBC was calculated both (i) between DHA controlled genes and anthropometric nodes and (ii) between DHA controlled genes and DHA controlled metabolomic/lipidomic data. Plotly (https://chart-studio.plotly.com/create/#/) was used to create the 2D contour histograms for visualization of the random network results.

Intrahepatic ligand–receptor interaction network

The knowledgebase of ligand–receptor interaction information available for mouse genes (Abugessaisa *et al*, 2021), was overlayed on to

 © 2023 The Authors

reconstructed NASH Multiomics network genes. Additionally, this allowed the interrogation of ligand–receptor network with respect to each cell in the network that a gene can be represented as ligand or receptor.

Human liver cancer meta-analysis

Human cancer data from hepatic cellular cancer (HCC) and cholangiocytes cancer (CC) were selected from GEO data sets (GSE14520, GSE26566, GSE102079, GSE56140, GSE98617, GSE76427, GSE84005). Meta-analysis using RankProd method described in the publicly available tool OMiCC (Shah *et al*, 2016) was carried out for these 7 sets of data with 32 healthy, 260 non-tumor samples and 544 tumor samples (Appendix Fig S3A). At first sample sets of both tumor types were compared against their respective paired non-tumor or healthy samples. Additionally, a standard (Fisher) approach of meta-analysis was also carried out and an overlapping set of genes were selected.

Then a signature set of genes were identified by matching (among orthologous) genes between mouse and human using Biomart (Cunningham *et al*, 2022) for the concordant fold change direction as WD + O/RD in the NASH network model and genes that are significant with FDR $< 15\%$ and Fisher P value $< 5\%$. The human liver cancer meta-analysis signature genes overlapped with the NASH mouse model network genes with treatment effects to identify subset of genes.

Gene ontology analyses

The gene ontology and functional enrichment were carried out using Metascape and innateDB (Breuer *et al*, 2013; Zhou *et al*, 2019) with mouse or human reference databases. The reference database is determined depending on the species of the specific data in question.

Pathway summary metric

Using published approach (Levine *et al*, 2006) with minor adaptions described in our previous papers (Shulzhenko *et al*, 2011; Koscso *et al*, 2020), each top gene ontology pathway enriched in the THP-1 experiment (with BTC and TLR ligands and significantly revered by DHA in NASH preventive model) was recognized and the genes belonging to the pathways were identified. Using median normalization, a value for each gene in each treatment replicates and then additionally using their median, summary metric was calculated for the individual pathway. A paired t-test between normal and treatment condition was performed to evaluate significance.

Upstream regulation and transcription factor analysis of BTC

The overall analysis strategy consisted of: (i) establishing functional effects of BTC represented by BTC-dependent gene expression profile. (ii) Using network analysis among genes regulated by omega-3 we infer candidate regulators of BTC-dependent profile expressed in cholangiocytes. (iii) searching for transcription factors among group of genes found is step 2; (iv) identifying which TF has a binding motif in BTC gene.

Specifically, identification of top BiBC involved in the regulation of Betacellulin in the cholangiocytes were carried out by interrogating the network nodes with the Omega 3 reversal effect in NASH network derived from *in vivo* data and assigned to cholangiocytes while opposite in BTC treatment *in vitro* in both LX2 and THP1 experiments to the lipids with EPA/DHA acyl chain in them (Appendix Fig S6A and B). Then from this list of BiBC nodes, the genes with higher than 50 edges/degrees and top 1% < BiBC were further analyzed for transcription factor (TF) activity. Using the TF binding prediction analysis tools (SCENIC, *TFBSPred*; Aibar *et al*, 2017; Zogopoulos *et al*, 2021), the TF motif and binding site in the promoter of Btc were predicted and verified.

The paper explained

Problem

Metabolic-associated fatty liver disease (MAFLD, also known as non-alcoholic fatty liver disease, NAFLD) and non-alcoholic steatohepatitis (NASH) are major risks factor for cirrhosis, hepatocellular carcinoma, and liver failure. Lack of specific FDA-approved treatments makes this a prominent health issue affecting roughly one third of the population, particularly in the Americas and South-East Asia. Although omega-3 fatty acids are known for their positive effects on the liver in NASH, a comprehensive analysis to identify the key molecular factors involved is still necessary.

Results

In this work, we used top-down system biology approach with causal inference analysis to reconstruct a multi-omic network (transcriptome, metabolome, lipidome, single cell RNA sequencing) and to reveal main molecular players responsible for beneficial effects of ω3 fatty acids on NASH and potentially on liver cancer. In agreement with our previous findings, out of two omega-3 fatty acids tested, docosahexaenoic acid (DHA) was more potent than eicosapentaenoic acid (EPA) in its effects on multi-omic readouts. In our search for NASH mechanisms that also play a role in liver cancer, we found that a key aspect of omega-3 fatty acid action involves inhibiting the expression and function of betacellulin (BTC), a less studied member of the epidermal growth factor family. We verified our network predictions *in vitro* and found that BTC stimulates TGFβ-2-driven collagen production in hepatic stellate cells and enhances microbial signals in the induction of integrins by macrophages. These processes work together to promote liver fibrosis and inflammation. Conversely, omega-3 fatty acids, especially DHA, can interrupt and even reverse these processes. Additionally, we identified the transcription factor FOXO3 as the most likely upstream regulator of the effects of omega-3 fatty acids on BTC.

Impact

Our research has revealed a novel mechanism through which omega-3 fatty acids regulate liver health, mitigating harmful processes during NASH. These newfound mechanistic insights have the potential to facilitate the development of innovative therapies that target the BTC pathway for NASH treatment and liver cancer prevention. Additionally, the gene expression signature triggered by BTC holds promise as a potential biomarker for guiding clinical trials involving ω3 PUFA, potentially advancing personalized medicine for liver disease management.

Statistical analysis

In mouse studies, data are expressed as geometric means of replicates. Data are shown as the mean ± standard deviation in animal studies and *in vitro* experiments. Group comparisons were performed using an unpaired t test and ordinary one-way analysis of variance (ANOVA), followed by Tukey's *post hoc* or Dunnett's multiple comparisons tests, where $P < 0.05$ indicates statistical significance. In cell line and RNA sequence analysis, comparisons

© 2023 The Authors

between groups were performed using Student's *t* test or the Mann–Whitney *U* test and Kruskal-Wallis test when appropriate. Categorical variables are shown as counts and percentages. Differences between categorical variables were assessed with the chi-squared test or Fisher's exact test. Spearman's rank correlation rho coefficients were calculated for network edges between all parameters using R statistical packages. Details of statistical analyses are described additionally in the corresponding figure legends. GraphPad Prism 9 was used for all analyses.

Data availability

RNA-Seq data: Gene Expression Omnibus GSE215223 (https://www.ncbi.nlm.nih.gov/geo/query/acc.cgi?acc=GSE215223).

RNA-Seq data: Gene Expression Omnibus GSE215224 (https://www.ncbi.nlm.nih.gov/geo/query/acc.cgi?acc=GSE215224).

RNA-Seq data: Gene Expression Omnibus GSE215225 (https://www.ncbi.nlm.nih.gov/geo/query/acc.cgi?acc=GSE215225).

RNA-Seq data: Gene Expression Omnibus GSE215227 (https://www.ncbi.nlm.nih.gov/geo/query/acc.cgi?acc=GSE215227).

Network Model: NASH_DHA_Betacellulin_Multi-omics_Network (https://www.ndexbio.org/#/network/60fc7ad9-3d93-11ee-aa50-005056ae23aa?accesskey=764ecdb2a2d2a6178586a7afd0755c9c8fab9e9a531b4336cf26b46432cac4a1).

Expanded View for this article is available online.

Acknowledgements

We would like to thank Dr. Scott Friedman from the Division of Liver Diseases at the Icahn School of Medicine at Mount Sinai for providing the LX2 cell line and Christiane V. Löhr, MedVet, Dr. med. vet., PhD for the evaluation of liver histology. And the personnel of CQLS at the Oregon State University for IT support. Funding: US Department of Agriculture, National Institute of food and agriculture grant 2009-65200-05846 (DBJ), National Institutes of Health grants, R01 DK094600 (DBJ), R01 DK112360 (DBJ) and R01 DK103761 (NS).

Author contributions

Jyothi Padiadpu: Data curation; software; formal analysis; supervision; validation; investigation; visualization; methodology; writing – original draft; writing – review and editing. **Manuel Garcia-Jaramillo:** Data curation; formal analysis; investigation; methodology. **Nolan K Newman:** Data curation; software; formal analysis; validation; investigation; visualization; methodology; writing – original draft; writing – review and editing. **Jacob W Pederson:** Validation; investigation; visualization; methodology. **Richard Rodrigues:** Software; formal analysis; investigation; methodology. **Zhipeng Li:** Formal analysis; investigation; methodology. **Sehajvir Singh:** Formal analysis; investigation. **Philip Monnier:** Formal analysis; investigation; methodology. **Giorgio Trinchieri:** Resources; investigation; methodology. **Kevin Brown:** Resources; software; methodology. **Amiran K Dzutsev:** Resources; investigation; methodology. **Natalia Shulzhenko:** Resources; supervision; funding acquisition; investigation; visualization; methodology; project administration; writing – review and editing. **Donald B Jump:** Conceptualization; resources; formal analysis; supervision; funding acquisition; investigation; methodology; writing – original draft; project administration; writing – review and editing. **Andrey Morgun:** Conceptualization; resources; software; formal analysis; supervision; visualization; methodology; writing – original draft; project administration; writing – review and editing.

Disclosure and competing interests statement

The authors declare that they have no conflict of interest.

References

Abugessaisa I, Ramilowski JA, Lizio M, Severin J, Hasegawa A, Harshbarger J, Kondo A, Noguchi S, Yip CW, Ooi JLC *et al* (2021) FANTOM enters 20th year: expansion of transcriptomic atlases and functional annotation of non-coding RNAs. *Nucleic Acids Res* 49: D892–d898

Agarwal SK (2014) Integrins and cadherins as therapeutic targets in fibrosis. *Front Pharmacol* 5: 131

Ahrens M, Ammerpohl O, von Schonfels W, Kolarova J, Bens S, Itzel T, Teufel A, Herrmann A, Brosch M, Hinrichsen H *et al* (2013) DNA methylation analysis in nonalcoholic fatty liver disease suggests distinct disease-specific and remodeling signatures after bariatric surgery. *Cell Metab* 18: 296–302

Aibar S, Gonzalez-Blas CB, Moerman T, Huynh-Thu VA, Imrichova H, Hulselmans G, Rambow F, Marine JC, Geurts P, Aerts J *et al* (2017) SCENIC: single-cell regulatory network inference and clustering. *Nat Methods* 14: 1083–1086

Ampuero J, Gallego-Durán R, Maya-Miles D, Montero R, Gato S, Rojas Á, Gil A, Muñoz R, Romero-Gómez M (2022) Systematic review and meta-analysis: analysis of variables influencing the interpretation of clinical trial results in NAFLD. *J Gastroenterol* 57: 357–371

Anstee QM, Reeves HL, Kotsiliti E, Govaere O, Heikenwalder M (2019) From NASH to HCC: current concepts and future challenges. *Nat Rev Gastroenterol Hepatol* 16: 411–428

Bhatt DL, Steg PG, Miller M, Brinton EA, Jacobson TA, Ketchum SB, Doyle RT Jr, Juliano RA, Jiao L, Granowitz C *et al* (2019) Cardiovascular risk reduction with icosapent ethyl for hypertriglyceridemia. *N Engl J Med* 380: 11–22

Bourgot I, Primac I, Louis T, Noël A, Maquoi E (2020) Reciprocal interplay between fibrillar collagens and collagen-binding integrins: implications in cancer progression and metastasis. *Front Oncol* 10: 1488

Breuer K, Foroushani AK, Laird MR, Chen C, Sribnaia A, Lo R, Winsor GL, Hancock RE, Brinkman FS, Lynn DJ (2013) InnateDB: systems biology of innate immunity and beyond–recent updates and continuing curation. *Nucleic Acids Res* 41: D1228–D1233

Burke PA, Ling PR, Forse RA, Bistrian BR (1999) Conditionally essential fatty acid deficiencies in end-stage liver disease. *Nutrition* 15: 302–304

Carter JK, Friedman SL (2022) Hepatic stellate cell-immune interactions in NASH. *Front Endocrinol (Lausanne)* 13: 867940

Chava S, Bugide S, Zhang X, Gupta R, Wajapeyee N (2022) Betacellulin promotes tumor development and EGFR mutant lung cancer growth by stimulating the EGFR pathway and suppressing apoptosis. *iScience* 25: 104211

Chen J, Zeng F, Forrester SJ, Eguchi S, Zhang MZ, Harris RC (2016) Expression and function of the epidermal growth factor receptor in physiology and disease. *Physiol Rev* 96: 1025–1069

Chen Z, Chen L, Sun B, Liu D, He Y, Qi L, Li G, Han Z, Zhan L, Zhang S *et al* (2021) LDLR inhibition promotes hepatocellular carcinoma proliferation and metastasis by elevating intracellular cholesterol synthesis through the MEK/ERK signaling pathway. *Mol Metab* 51: 101230

Choobdar S, Ahsen ME, Crawford J, Tomasoni M, Fang T, Lamparter D, Lin J, Hescott B, Hu X, Mercer J *et al* (2019) Assessment of network module identification across complex diseases. *Nat Methods* 16: 843–852

Costa C, Sampaio-Maia B, Araujo R, Nascimento DS, Ferreira-Gomes J, Pestana M, Azevedo MJ, Alencastre IS (2022) Gut microbiome and organ fibrosis. *Nutrients* 14: 352

© 2023 The Authors

Cunningham F, Allen JE, Allen J, Alvarez-Jarreta J, Amode MR, Armean IM, Austine-Orimoloye O, Azov AG, Barnes I, Bennett R *et al* (2022) Ensembl 2022. *Nucleic Acids Res* 50: D988–D995

Dahlén AD, Dashi G, Maslov I, Attwood MM, Jonsson J, Trukhan V, Schiöth HB (2021) Trends in antidiabetic drug discovery: FDA approved drugs, new drugs in clinical trials and global sales. *Front Pharmacol* 12: 807548

Dahlhoff M, Wolf E, Schneider MR (2014) The ABC of BTC: structural properties and biological roles of betacellulin. *Semin Cell Dev Biol* 28: 42–48

Davidson MH, Stein EA, Bays HE, Maki KC, Doyle RT, Shalwitz RA, Ballantyne CM, Ginsberg HN (2007) Efficacy and tolerability of adding prescription omega-3 fatty acids 4 g/d to simvastatin 40 mg/d in hypertriglyceridemic patients: an 8-week, randomized, double-blind, placebo-controlled study. *Clin Ther* 29: 1354–1367

Depner CM, Philbrick KA, Jump DB (2013a) Docosahexaenoic acid attenuates hepatic inflammation, oxidative stress, and fibrosis without decreasing hepatosteatosis in a Ldlr(−/−) mouse model of western diet-induced nonalcoholic steatohepatitis. *J Nutr* 143: 315–323

Depner CM, Traber MG, Bobe G, Kensicki E, Bohren KM, Milne G, Jump DB (2013b) A metabolomic analysis of omega-3 fatty acid-mediated attenuation of western diet-induced nonalcoholic steatohepatitis in LDLR$^{-/-}$ mice. *PLoS One* 8: e83756

Dierge E, Debock E, Guilbaud C, Corbet C, Mignolet E, Mignard L, Bastien E, Dessy C, Larondelle Y, Feron O (2021) Peroxidation of n-3 and n-6 polyunsaturated fatty acids in the acidic tumor environment leads to ferroptosis-mediated anticancer effects. *Cell Metab* 33: 1701–1715.e1705

Dong X, Yambartsev A, Ramsey SA, Thomas LD, Shulzhenko N, Morgun A (2015) Reverse enGENEering of Regulatory Networks from Big Data: a roadmap for biologists. *Bioinform Biol Insights* 9: 61–74

Dropmann A, Dediulia T, Breitkopf-Heinlein K, Korhonen H, Janicot M, Weber SN, Thomas M, Piiper A, Bertran E, Fabregat I *et al* (2016) TGF-β1 and TGF-β2 abundance in liver diseases of mice and men. *Oncotarget* 7: 19499–19518

Dufour JF, Anstee QM, Bugianesi E, Harrison S, Loomba R, Paradis V, Tilg H, Wong VW, Zelber-Sagi S (2022) Current therapies and new developments in NASH. *Gut* 71: 2123–2134

Fridén M, Rosqvist F, Ahlström H, Niessen HG, Schultheis C, Hockings P, Hulthe J, Gummesson A, Wanders A, Rorsman F *et al* (2021) Hepatic unsaturated fatty acids are linked to lower degree of fibrosis in non-alcoholic fatty liver disease. *Front Med (Lausanne)* 8: 814951

Fujiwara N, Kubota N, Crouchet E, Koneru B, Marquez CA, Jajoriya AK, Panda G, Qian T, Zhu S, Goossens N *et al* (2022) Molecular signatures of long-term hepatocellular carcinoma risk in nonalcoholic fatty liver disease. *Sci Transl Med* 14: eabo4474

Garcia-Jaramillo M, Spooner MH, Löhr CV, Wong CP, Zhang W, Jump DB (2019) Lipidomic and transcriptomic analysis of western diet-induced nonalcoholic steatohepatitis (NASH) in female Ldlr$^{-/-}$ mice. *PloS One* 14: e0214387

Green CD, Weigel C, Brown RDR, Bedossa P, Dozmorov M, Sanyal AJ, Spiegel S (2022) A new preclinical model of western diet-induced progression of non-alcoholic steatohepatitis to hepatocellular carcinoma. *FASEB J* 36: e22372

Gutiérrez S, Svahn SL, Johansson ME (2019) Effects of omega-3 fatty acids on immune cells. *Int J Mol Sci* 20: 5028

Hammad S, Ogris C, Othman A, Erdoesi P, Schmidt-Heck W, Biermayer I, Helm B, Gao Y, Pioronska W, Holland CH *et al* (2023) Tolerance of repeated toxic injuries of murine livers is associated with steatosis and inflammation. *Cell Death Dis* 14: 414

Harris WS, Ginsberg HN, Arunakul N, Shachter NS, Windsor SL, Adams M, Berglund L, Osmundsen K (1997) Safety and efficacy of Omacor in severe hypertriglyceridemia. *J Cardiovasc Risk* 4: 385–391

He Y, Sun MM, Zhang GG, Yang J, Chen KS, Xu WW, Li B (2021) Targeting PI3K/Akt signal transduction for cancer therapy. *Signal Transduct Target Ther* 6: 425

Hedegger K, Algül H, Lesina M, Blutke A, Schmid RM, Schneider MR, Dahlhoff M (2020) Unraveling ERBB network dynamics upon betacellulin signaling in pancreatic ductal adenocarcinoma in mice. *Mol Oncol* 14: 1653–1669

Hodson L, Bhatia L, Scorletti E, Smith DE, Jackson NC, Shojaee-Moradie F, Umpleby M, Calder PC, Byrne CD (2017) Docosahexaenoic acid enrichment in NAFLD is associated with improvements in hepatic metabolism and hepatic insulin sensitivity: a pilot study. *Eur J Clin Nutr* 71: 973–979

Hume DA, MacDonald KP (2012) Therapeutic applications of macrophage colony-stimulating factor-1 (CSF-1) and antagonists of CSF-1 receptor (CSF-1R) signaling. *Blood* 119: 1810–1820

Iannelli A, Martini F, Schneck AS, Ghavami B, Baudin G, Anty R, Gugenheim J (2013) Preoperative 4-week supplementation with omega-3 polyunsaturated fatty acids reduces liver volume and facilitates bariatric surgery in morbidly obese patients. *Obes Surg* 23: 1761–1765

Jastreboff AM, Aronne LJ, Ahmad NN, Wharton S, Connery L, Alves B, Kiyosue A, Zhang S, Liu B, Bunck MC *et al* (2022) Tirzepatide once weekly for the treatment of obesity. *N Engl J Med* 387: 205–216

Jermendy G, Kiss Z, Rokszin G, Abonyi-Tóth Z, Wittmann I, Kempler P (2018) Persistence to treatment with novel antidiabetic drugs (dipeptidyl peptidase-4 inhibitors, sodium-glucose co-transporter-2 inhibitors, and glucagon-like peptide-1 receptor agonists) in people with type 2 diabetes: a nationwide cohort study. *Diabetes Ther* 9: 2133–2141

Jump DB, Depner CM, Tripathy S, Lytle KA (2015) Potential for dietary ω-3 fatty acids to prevent nonalcoholic fatty liver disease and reduce the risk of primary liver cancer. *Adv Nutr* 6: 694–702

Jump DB, Lytle KA, Depner CM, Tripathy S (2018) Omega-3 polyunsaturated fatty acids as a treatment strategy for nonalcoholic fatty liver disease. *Pharmacol Ther* 181: 108–125

Kahalehili HM, Newman NK, Pennington JM, Kolluri SK, Kerkvliet NI, Shulzhenko N, Morgun A, Ehrlich AK (2020) Dietary indole-3-carbinol activates AhR in the gut, alters Th17-microbe interactions, and exacerbates insulitis in NOD mice. *Front Immunol* 11: 606441

Konerman MA, Jones JC, Harrison SA (2018) Pharmacotherapy for NASH: Current and emerging. *J Hepatol* 68: 362–375

Koscso B, Kurapati S, Rodrigues RR, Nedjic J, Gowda K, Shin C, Soni C, Ashraf AZ, Purushothaman I, Palisoc M *et al* (2020) Gut-resident CX3CR1(hi) macrophages induce tertiary lymphoid structures and IgA response *in situ*. *Sci Immunol* 5: eaax0062

Lam KC, Araya RE, Huang A, Chen Q, Di Modica M, Rodrigues RR, Lopes A, Johnson SB, Schwarz B, Bohrnsen E *et al* (2021) Microbiota triggers STING-type I IFN-dependent monocyte reprogramming of the tumor microenvironment. *Cell* 184: 5338–5356.e21

Lanaya H, Natarajan A, Komposch K, Li L, Amberg N, Chen L, Wculek SK, Hammer M, Zenz R, Peck-Radosavljevic M *et al* (2014) EGFR has a tumour-promoting role in liver macrophages during hepatocellular carcinoma formation. *Nat Cell Biol* 16: 972–977

Lazarus JV, Mark HE, Anstee QM, Arab JP, Batterham RL, Castera L, Cortez-Pinto H, Crespo J, Cusi K, Dirac MA *et al* (2022) Advancing the global public health agenda for NAFLD: a consensus statement. *Nat Rev Gastroenterol Hepatol* 19: 60–78

Levine DM, Haynor DR, Castle JC, Stepaniants SB, Pellegrini M, Mao M, Johnson JM (2006) Pathway and gene-set activation measurement from

© 2023 The Authors

mRNA expression data: the tissue distribution of human pathways. *Genome Biol* 7: R93

Li Z, Gurung M, Rodrigues RR, Padiadpu J, Newman NK, Manes NP, Pederson JW, Greer RL, Vasquez-Perez S, You H *et al* (2022) Microbiota and adipocyte mitochondrial damage in type 2 diabetes are linked by Mmp12+ macrophages. *J Exp Med* 219: e20220017

Liu Y, Ao X, Ding W, Ponnusamy M, Wu W, Hao X, Yu W, Wang Y, Li P, Wang J (2018) Critical role of FOXO3a in carcinogenesis. *Mol Cancer* 17: 104

Lytle KA, Depner CM, Wong CP, Jump DB (2015) Docosahexaenoic acid attenuates Western diet-induced hepatic fibrosis in Ldlr$^{-/-}$ mice by targeting the TGFβ-Smad3 pathway. *J Lipid Res* 56: 1936–1946

Lytle KA, Wong CP, Jump DB (2017) Docosahexaenoic acid blocks progression of western diet-induced nonalcoholic steatohepatitis in obese Ldlr$^{-/-}$ mice. *PloS One* 12: e0173376

Marjot T, Moolla A, Cobbold JF, Hodson L, Tomlinson JW (2020) Nonalcoholic fatty liver disease in adults: current concepts in etiology, outcomes, and management. *Endocr Rev* 41: 66–117

Miura K, Yang L, van Rooijen N, Brenner DA, Ohnishi H, Seki E (2013) Toll-like receptor 2 and palmitic acid cooperatively contribute to the development of nonalcoholic steatohepatitis through inflammasome activation in mice. *Hepatology* 57: 577–589

Moon WS, Park HS, Yu KH, Park MY, Kim KR, Jang KY, Kim JS, Cho BH (2006) Expression of betacellulin and epidermal growth factor receptor in hepatocellular carcinoma: implications for angiogenesis. *Hum Pathol* 37: 1324–1332

Morgun A, Dzutsev A, Dong X, Greer RL, Sexton DJ, Ravel J, Schuster M, Hsiao W, Matzinger P, Shulzhenko N (2015) Uncovering effects of antibiotics on the host and microbiota using transkingdom gene networks. *Gut* 64: 1732–1743

Musa-Veloso K, Venditti C, Lee HY, Darch M, Floyd S, West S, Simon R (2018) Systematic review and meta-analysis of controlled intervention studies on the effectiveness of long-chain omega-3 fatty acids in patients with nonalcoholic fatty liver disease. *Nutr Rev* 76: 581–602

Neuschwander-Tetri BA (2020) Therapeutic landscape for NAFLD in 2020. *Gastroenterology* 158: 1984–1998.e1983

Okada L, Oliveira CP, Stefano JT, Nogueira MA, Silva I, Cordeiro FB, Alves VAF, Torrinhas RS, Carrilho FJ, Puri P *et al* (2018) Omega-3 PUFA modulate lipogenesis, ER stress, and mitochondrial dysfunction markers in NASH – proteomic and lipidomic insight. *Clin Nutr* 37: 1474–1484

Olayioye MA, Neve RM, Lane HA, Hynes NE (2000) The ErbB signaling network: receptor heterodimerization in development and cancer. *EMBO J* 19: 3159–3167

Pfister D, Núñez NG, Pinyol R, Govaere O, Pinter M, Szydlowska M, Gupta R, Qiu M, Deczkowska A, Weiner A *et al* (2021) NASH limits anti-tumour surveillance in immunotherapy-treated HCC. *Nature* 592: 450–456

Rahman SR, Roper JA, Grove JI, Aithal GP, Pun KT, Bennett AJ (2022) Integrins as a drug target in liver fibrosis. *Liver Int* 42: 507–521

Ramachandran P, Dobie R, Wilson-Kanamori JR, Dora EF, Henderson BEP, Luu NT, Portman JR, Matchett KP, Brice M, Marwick JA *et al* (2019) Resolving the fibrotic niche of human liver cirrhosis at single-cell level. *Nature* 575: 512–518

Rinella ME (2015) Nonalcoholic fatty liver disease: a systematic review. *JAMA* 313: 2263–2273

Rodrigues RR, Gurung M, Li Z, Garcia-Jaramillo M, Greer R, Gaulke C, Bauchinger F, You H, Pederson JW, Vasquez-Perez S *et al* (2021) Transkingdom interactions between Lactobacilli and hepatic mitochondria attenuate western diet-induced diabetes. *Nat Commun* 12: 101

Samuel VT, Shulman GI (2018) Nonalcoholic fatty liver disease as a nexus of metabolic and hepatic diseases. *Cell Metab* 27: 22–41

Seidman JS, Troutman TD, Sakai M, Gola A, Spann NJ, Bennett H, Bruni CM, Ouyang Z, Li RZ, Sun X *et al* (2020) Niche-specific reprogramming of epigenetic landscapes drives myeloid cell diversity in nonalcoholic steatohepatitis. *Immunity* 52: 1057–1074.e1057

Seki E, De Minicis S, Osterreicher CH, Kluwe J, Osawa Y, Brenner DA, Schwabe RF (2007) TLR4 enhances TGF-beta signaling and hepatic fibrosis. *Nat Med* 13: 1324–1332

Shah N, Guo Y, Wendelsdorf KV, Lu Y, Sparks R, Tsang JS (2016) A crowdsourcing approach for reusing and meta-analyzing gene expression data. *Nat Biotechnol* 34: 803–806

Shi L, Wang L, Wang B, Cretoiu SM, Wang Q, Wang X, Chen C (2014) Regulatory mechanisms of betacellulin in CXCL8 production from lung cancer cells. *J Transl Med* 12: 70

Shulzhenko N, Morgun A, Hsiao W, Battle M, Yao M, Gavrilova O, Orandle M, Mayer L, Macpherson AJ, McCoy KD *et al* (2011) Crosstalk between B lymphocytes, microbiota and the intestinal epithelium governs immunity versus metabolism in the gut. *Nat Med* 17: 1585–1593

Simões ICM, Fontes A, Pinton P, Zischka H, Wieckowski MR (2018) Mitochondria in non-alcoholic fatty liver disease. *Int J Biochem Cell Biol* 95: 93–99

Šmíd V, Dvořák K, Šedivý P, Kosek V, Leníček M, Dezortová M, Hajšlová J, Hájek M, Vítek L, Bechyňská K *et al* (2022) Effect of omega-3 polyunsaturated fatty acids on lipid metabolism in patients with metabolic syndrome and NAFLD. *Hepatol Commun* 6: 1336–1349

Sorrells TR, Johnson AD (2015) Making sense of transcription networks. *Cell* 161: 714–723

Spooner MH, Jump DB (2019) Omega-3 fatty acids and nonalcoholic fatty liver disease in adults and children: where do we stand? *Curr Opin Clin Nutr Metab Care* 22: 103–110

Spruss A, Kanuri G, Wagnerberger S, Haub S, Bischoff SC, Bergheim I (2009) Toll-like receptor 4 is involved in the development of fructose-induced hepatic steatosis in mice. *Hepatology* 50: 1094–1104

Stuart T, Butler A, Hoffman P, Hafemeister C, Papalexi E, Mauck WM 3rd, Hao Y, Stoeckius M, Smibert P, Satija R (2019) Comprehensive integration of single-cell data. *Cell* 177: 1888–1902

Sun T, Huang Z, Liang WC, Yin J, Lin WY, Wu J, Vernes JM, Lutman J, Caplazi P, Jeet S *et al* (2021) TGFβ2 and TGFβ3 isoforms drive fibrotic disease pathogenesis. *Sci Transl Med* 13: eabe0407

Tobin D, Brevik-Andersen M, Qin Y, Innes JK, Calder PC (2018) Evaluation of a high concentrate omega-3 for correcting the omega-3 fatty acid nutritional deficiency in non-alcoholic fatty liver disease (CONDIN). *Nutrients* 10: 1126

Tsuji T, Maeda Y, Kita K, Murakami K, Saya H, Takemura H, Inaki N, Oshima M, Oshima H (2021) FOXO3 is a latent tumor suppressor for FOXO3-positive and cytoplasmic-type gastric cancer cells. *Oncogene* 40: 3072–3086

Van Blarigan EL, Fuchs CS, Niedzwiecki D, Ye X, Zhang S, Song M, Saltz LB, Mayer RJ, Mowat RB, Whittom R *et al* (2018) Marine ω-3 polyunsaturated fatty acid and fish intake after colon cancer diagnosis and survival: CALGB 89803 (alliance). *Cancer Epidemiol Biomarkers Prev* 27: 438–445

Watson H, Mitra S, Croden FC, Taylor M, Wood HM, Perry SL, Spencer JA, Quirke P, Toogood GJ, Lawton CL *et al* (2018) A randomised trial of the effect of omega-3 polyunsaturated fatty acid supplements on the human intestinal microbiota. *Gut* 67: 1974–1983

Wattacheril J, Issa D, Sanyal A (2018) Nonalcoholic steatohepatitis (NASH) and hepatic fibrosis: emerging therapies. *Annu Rev Pharmacol Toxicol* 58: 649–662

Wieduwilt MJ, Moasser MM (2008) The epidermal growth factor receptor family: biology driving targeted therapeutics. *Cell Mol Life Sci* 65: 1566–1584

 © 2023 The Authors

Wu L, Sun J, Liu L, Du X, Liu Y, Yan X, Kombo Osoro E, Zhang F, Feng L, Liang D *et al* (2020) Anti-toll-like receptor 2 antibody ameliorates hepatic injury, inflammation, fibrosis and steatosis in obesity-related metabolic disorder rats via regulating MAPK and NF-κB pathways. *Int Immunopharmacol* 82: 106368

Wynn TA, Vannella KM (2016) Macrophages in tissue repair, regeneration, and fibrosis. *Immunity* 44: 450–462

Xie N, Zhang L, Gao W, Huang C, Huber PE, Zhou X, Li C, Shen G, Zou B (2020) NAD(+) metabolism: pathophysiologic mechanisms and therapeutic potential. *Signal Transduct Target Ther* 5: 227

Xiong X, Kuang H, Ansari S, Liu T, Gong J, Wang S, Zhao XY, Ji Y, Li C, Guo L *et al* (2019) Landscape of intercellular crosstalk in healthy and NASH liver revealed by single-cell secretome gene analysis. *Mol Cell* 75: 644–660.e645

Xu L, Hui AY, Albanis E, Arthur MJ, O'Byrne SM, Blaner WS, Mukherjee P, Friedman SL, Eng FJ (2005) Human hepatic stellate cell lines, LX-1 and LX-2: new tools for analysis of hepatic fibrosis. *Gut* 54: 142–151

Yambartsev A, Perlin MA, Kovchegov Y, Shulzhenko N, Mine KL, Dong X, Morgun A (2016) Unexpected links reflect the noise in networks. *Biol Direct* 11: 52

Yang J, Fernández-Galilea M, Martínez-Fernández L, González-Muniesa P, Pérez-Chávez A, Martínez JA, Moreno-Aliaga MJ (2019) Oxidative stress and non-alcoholic fatty liver disease: effects of omega-3 fatty acid supplementation. *Nutrients* 11: 872

Zhou Y, Zhou B, Pache L, Chang M, Khodabakhshi AH, Tanaseichuk O, Benner C, Chanda SK (2019) Metascape provides a biologist-oriented resource for the analysis of systems-level datasets. *Nat Commun* 10: 1523

Zogopoulos VL, Spaho K, Ntouka C, Lappas GA, Kyranis I, Bagos PG, Spandidos DA, Michalopoulos I (2021) TFBSPred: a functional transcription factor binding site prediction webtool for humans and mice. *Int J Epigenet* 1: 1–11

Zöhrer E, Alisi A, Jahnel J, Mosca A, Della Corte C, Crudele A, Fauler G, Nobili V (2017) Efficacy of docosahexaenoic acid-choline-vitamin E in paediatric NASH: a randomized controlled clinical trial. *Appl Physiol Nutr Metab* 42: 948–954

License: This is an open access article under the terms of the Creative Commons Attribution License, which permits use, distribution and reproduction in any medium, provided the original work is properly cited.

Expanded View Figures

Figure EV1. Extent of DHA reversal effects are significantly higher than those of EPA.

A–C The Log_2FC of EPA & DHA gene expression (A) and lipids and metabolites (B, C) show the extent of DHA reversal effects as significantly higher than EPA though similar in profile. The up and down regulation are shown in separate plots for clarity (A, B) (paired, two-sided *t*-test, ns [not significant], ****$P < 0.0001$).

C Scatterplot of fold change differences between WD + O and EPA (*x*-axis) or DHA (*y*-axis) treated mice with number of lipids and metabolites regulated similarly by DHA & EPA displayed (Pearson's Chi-squared test, **$P < 0.004$).

D Heatmap of differentially expressed genes in individual mice (WD + O vs. RD fed mice and organized by treatment effect category: DHA, EPA, or EPA & DHA). Data shown are the log transformed quantile normalized expression for each gene per prevention group. Row max is displayed as red, row min is displayed as blue.

© 2023 The Authors

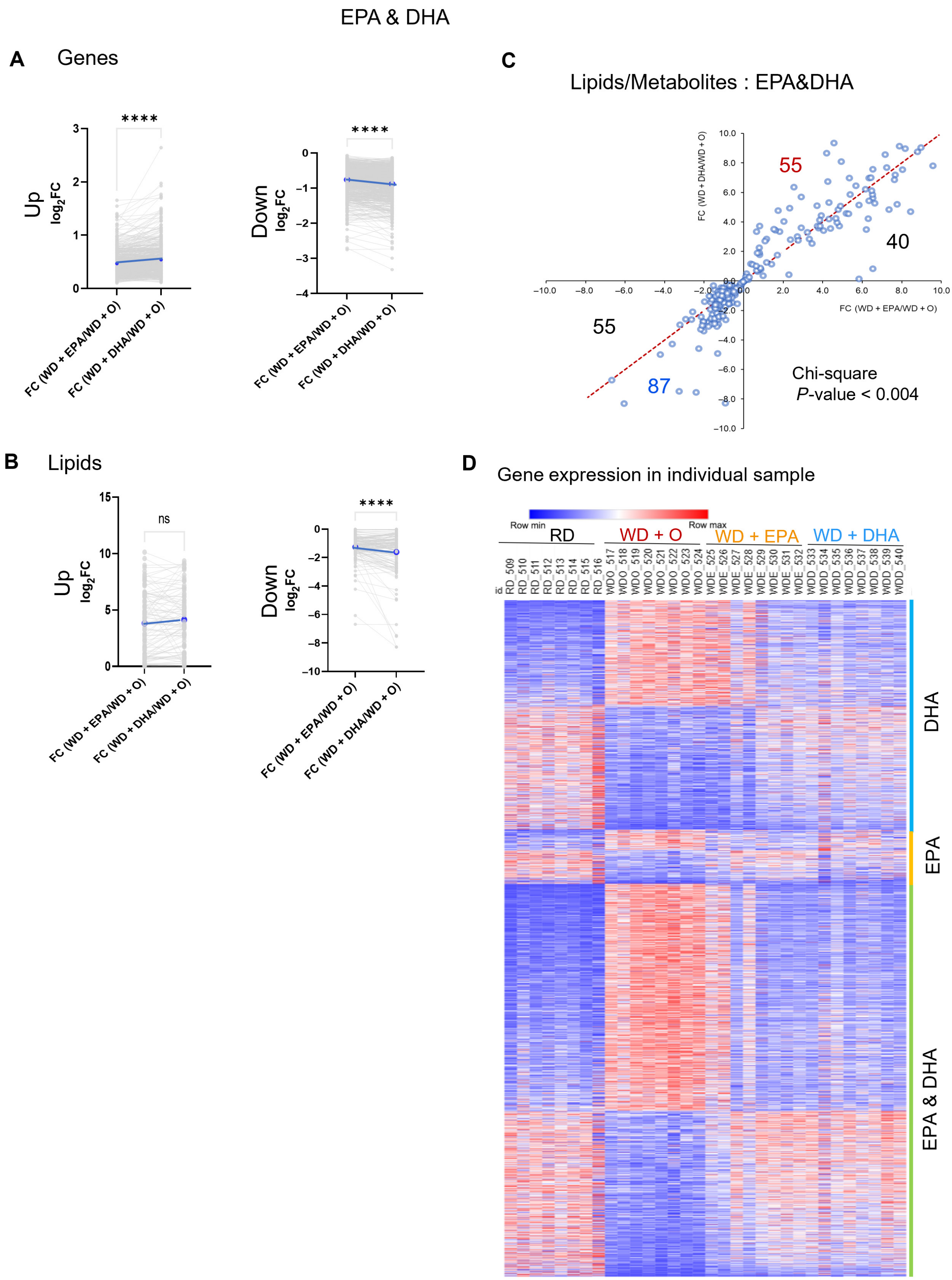
EPA & DHA
A Genes
Up log2FC
Down log2FC
****
FC (WD + EPA/WD + O)
FC (WD + DHA/WD + O)
B Lipids
ns
C
Lipids/Metabolites : EPA&DHA
FC (WD + DHA/WD + O)
FC (WD + EPA/WD + O)
55
40
55
87
Chi-square
P-value < 0.004
D
Gene expression in individual sample
Row min
Row max
RD
WD + O
WD + EPA
WD + DHA
DHA
EPA
EPA & DHA

Figure EV1.

Figure EV2. Interrogation of network model from the multi-omic NASH data.

A An outline with the steps involved in deriving, analyzing and interrogation of network model from the multi-omic NASH data (see Materials and Methods; GE: gene expression; P: Phenotype/Anthropometric/Biochemical data; LM: Lipids/Metabolites; PUC: Proportion of unexpected correlation). The distribution of treatment effects from the NASH preventive model among the network parameters is shown in the right panel.

B Outline of cell–cell interactions used to calculate the interaction BiBCs (see Materials and Methods). The nodes represent all genes part of cell types as a cluster in the network and edges are the interaction strength among the nodes. The node size indicates the number of genes represented by the cell type; color chart is proportional to the treatment effects in each cell type.

C Box plot of maximum cell–cell interaction BiBC for the genes belonging to each treatment effect category (DHA [blue], EPA&DHA [green], EPA [orange] and no category [gray]). From the network cell–cell BiBC analysis, genes regulated by DHA, DHA&EPA have higher BiBC that indicates a higher contribution to cell–cell communication than genes regulated by EPA.

D Bar plots for abundance of top BiBC lipids, shown are the PGs (cardiolipin precursors) and SM in NASH preventive study (Data are mean ± SD, $N = 8$ mice/treatment group; Ordinary One-way ANOVA, multiple comparisons test with WD + O, ***$P < 0.005$, ****$P < 0.0001$).

© 2023 The Authors

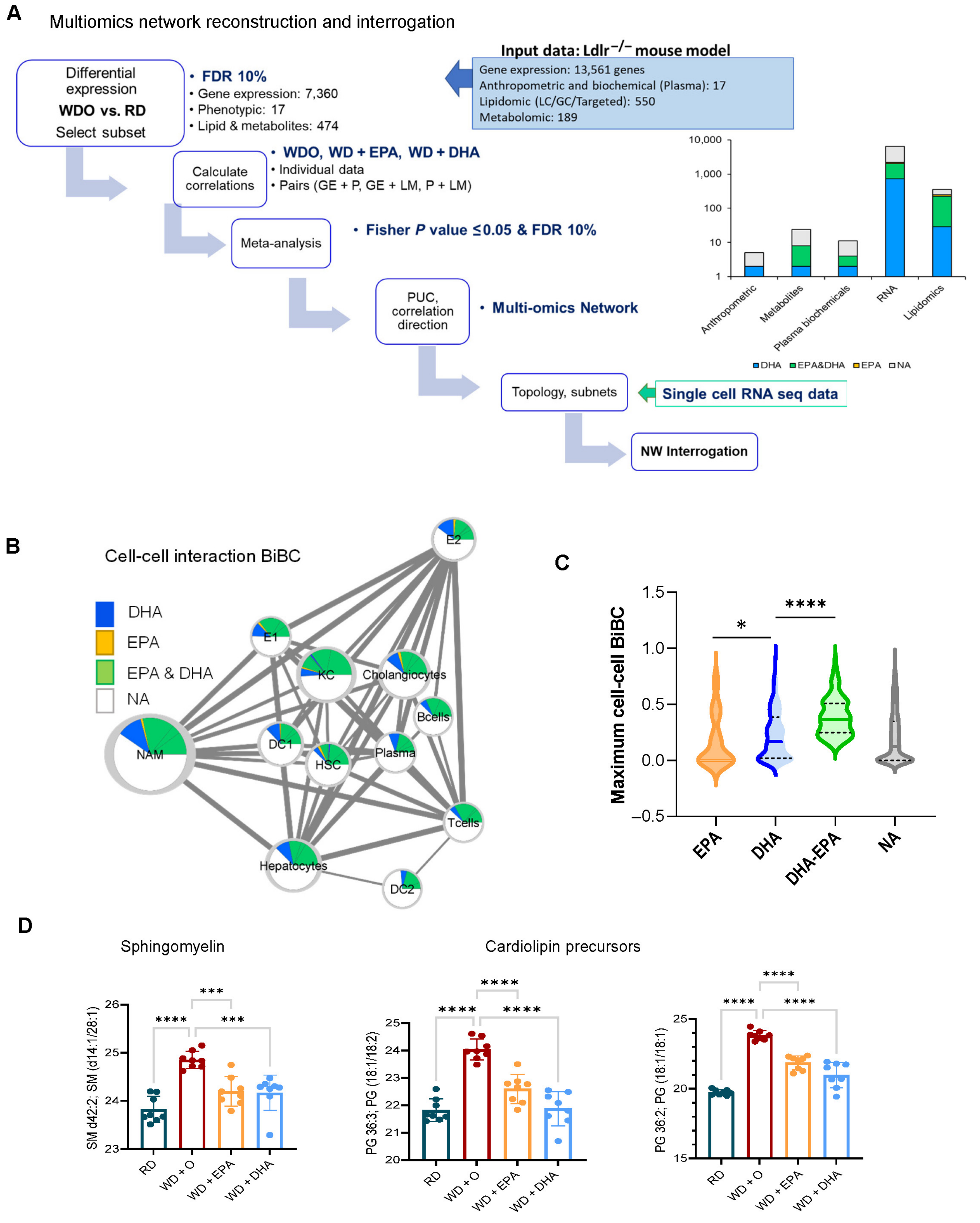
A
Multiomics network reconstruction and interrogation
Input data: Ldlr−/− mouse model
Gene expression: 13,561 genes
Anthropometric and biochemical (Plasma): 17
Lipidomic (LC/GC/Targeted): 550
Metabolomic: 189
Differential expression
WDO vs. RD
Select subset
• FDR 10%
• Gene expression: 7,360
• Phenotypic: 17
• Lipid & metabolites: 474
Calculate correlations
• WDO, WD + EPA, WD + DHA
• Individual data
• Pairs (GE + P, GE + LM, P + LM)
Meta-analysis
• Fisher P value ≤0.05 & FDR 10%
PUC, correlation direction
• Multi-omics Network
Topology, subnets
Single cell RNA seq data
NW Interrogation
Anthropometric
Metabolites
Plasma biochemicals
RNA
Lipidomics
DHA EPA&DHA EPA NA
B
Cell-cell interaction BiBC
DHA
EPA
EPA & DHA
NA
E2
E1
KC
Cholangiocytes
Bcells
NAM
DC1
HSC
Plasma
Tcells
Hepatocytes
DC2
C
Maximum cell-cell BiBC
EPA
DHA
DHA-EPA
NA
D
Sphingomyelin
Cardiolipin precursors
SM d42:2; SM (d14:1/28:1)
PG 36:3; PG (18:1/18:2)
PG 36:2; PG (18:1/18:1)
RD
WD + O
WD + EPA
WD + DHA

Figure EV2.

Figure EV3. Liver cancer meta-analysis and network interrogation points to significance of BTC.

A Outline for human liver cancer meta-analysis for the orthologous genes representing the NASH mouse model. Right panel shows the heatmap of individual human datasets for selected genes in the meta-analysis (with treatment effects in NASH model).

B The bar graph of gene set enrichment analysis using GSEA (KEGG pathways) for the human cancer meta-analysis genes with DHA effects in mouse models. Data are displayed as $-\log_{10}$(P-value).

C The gene expression for BTC-EGFR-ERBB pathway and cell cycle related genes in the NASH mouse preventive and treatment models. The color scale is indicated from high expression of the genes in red to low in blue.

D Distribution of Btc BiBC values calculated between DHA treatment reversed genes and metabolomic/lipidomic data (*x*-axis) and DHA controlled genes and anthropometric data (*y*-axis) in 5,000 random networks. Dark regions represent areas where a calculated Btc BiBC value is more likely to be found due to random chance. The probability of finding an actual Btc BiBC value equal to or higher than those seen in the random networks (43/5,000) is 0.009.

E Violin plot of the BiBC of Btc between DHA reversed genes and anthropometric data in 5,000 random networks compared to its actual BiBC value in the reconstructed network (one sample Wilcoxon test $P < 1 * 10^{-15}$).

© 2023 The Authors

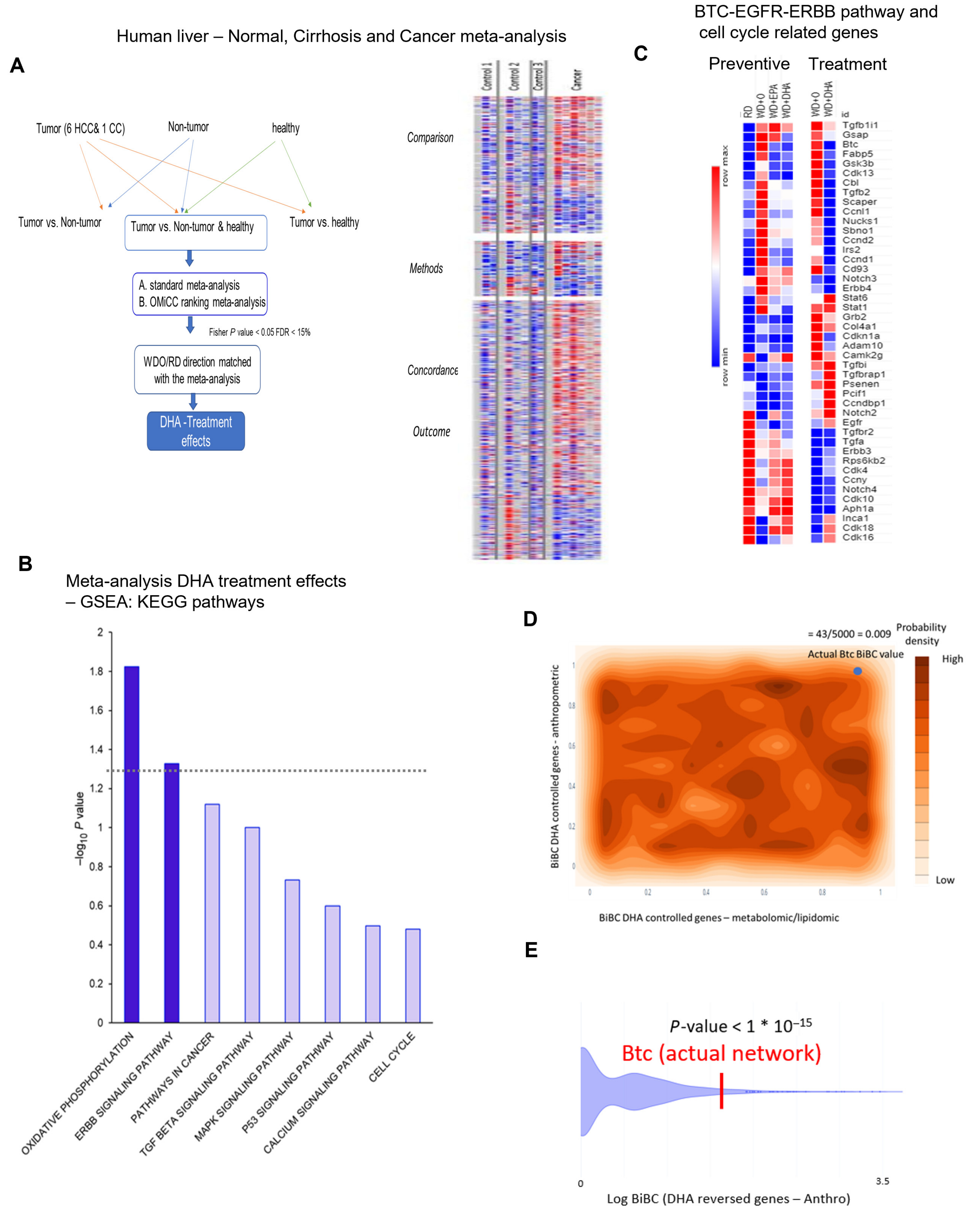
A
Human liver – Normal, Cirrhosis and Cancer meta-analysis
Tumor (6 HCC& 1 CC)
Non-tumor
healthy
Tumor vs. Non-tumor
Tumor vs. Non-tumor & healthy
Tumor vs. healthy
A. standard meta-analysis
B. OMiCC ranking meta-analysis
Fisher P value < 0.05 FDR < 15%
WDO/RD direction matched with the meta-analysis
DHA -Treatment effects
Control 1
Control 2
Control 3
Cancer
Comparison
Methods
Concordance
Outcome
B
Meta-analysis DHA treatment effects – GSEA: KEGG pathways
−log10 P value
OXIDATIVE PHOSPHORYLATION
ERBB SIGNALING PATHWAY
PATHWAYS IN CANCER
TGF BETA SIGNALING PATHWAY
MAPK SIGNALING PATHWAY
P53 SIGNALING PATHWAY
CALCIUM SIGNALING PATHWAY
CELL CYCLE
C
BTC-EGFR-ERBB pathway and cell cycle related genes
Preventive
Treatment
RD
WD+O
WD+EPA
WD+DHA
row max
row min
id
Tgfb1i1
Gsap
Btc
Fabp5
Gsk3b
Cdk13
Cbl
Tgfb2
Scaper
Ccnl1
Nucks1
Sbno1
Ccnd2
Irs2
Ccnd1
Cd93
Notch3
Erbb4
Stat6
Stat1
Grb2
Col4a1
Cdkn1a
Adam10
Camk2g
Tgfbi
Tgfbrap1
Psenen
Pcif1
Ccndbp1
Notch2
Egfr
Tgfbr2
Tgfa
Erbb3
Rps6kb2
Cdk4
Ccny
Notch4
Cdk10
Aph1a
Inca1
Cdk18
Cdk16
D
= 43/5000 = 0.009
Actual Btc BiBC value
Probability density
High
Low
BiBC DHA controlled genes - anthropometric
BiBC DHA controlled genes – metabolomic/lipidomic
E
P-value < 1 * 10−15
Btc (actual network)
Log BiBC (DHA reversed genes – Anthro)
0
3.5

Figure EV3.

Figure EV4. BTC and other Ligand-Receptor interaction network with DHA/EPA effects in NASH preventive mouse model.

A NASH Ligand–Receptor network with DHA/EPA effects in NASH preventive mouse model. The individual cells expressing the genes inferred from single cell RNA sequence reanalysis are overlayed and cell specific ligand–receptor interactions are shown. Nodes are shown as diamonds (ligands), or hexagon (receptors) and edges are the correlation between the nodes. Node color is according to the treatment effect. The thickness of the node is the cell–cell interaction BiBC, higher the better and is shown thicker. Each cell type and their respective genes are colored differently as labeled. The genes belonging to other cells in addition to specific cells are shown as part of 'Not assigned' group.

B The normalized Btc expression form others experiments (Left panel; Control and Nash fed with high fat high sugar mouse model; GSE197884; Mann–Whitney test, One-tailed, $N = 3$). (Right panel) The normalized Btc expression form other datasets (GSE222576; Control and CCl_4 treated liver fibrosis mouse model; Ordinary one-way ANOVA, $^{*}P < 0.05$, $^{**}P < 0.005$).

 © 2023 The Authors

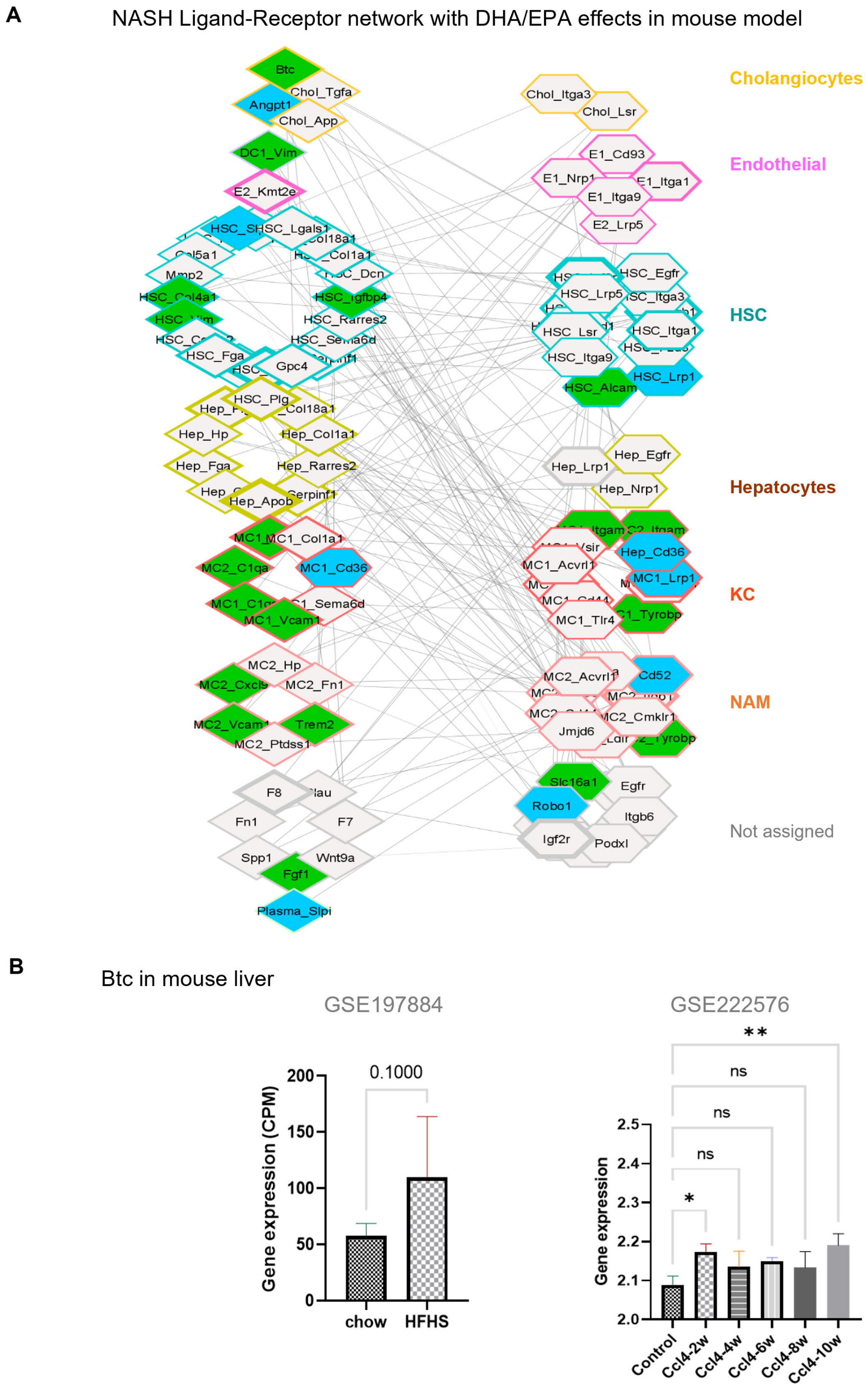
A
NASH Ligand-Receptor network with DHA/EPA effects in mouse model
Cholangiocytes
Endothelial
HSC
Hepatocytes
KC
NAM
Not assigned
Btc
Chol_Tgfa
Angpt1
Chol_App
DC1_Vim
E2_Kmt2e
HSC_Lgals1
HSC_Rarres2
HSC_Fga
Gpc4
HSC_Plg
Hep_Hp
Hep_Col1a1
Hep_Fga
Hep_Rarres2
Hep_Apob
MC1_Col1a1
MC2_C1qa
MC1_Cd36
MC1_Vcam1
MC2_Hp
MC2_Fn1
Trem2
MC2_Ptdss1
F8
Fn1
F7
Spp1
Wnt9a
Fgf1
Plasma_Slpi
Chol_Itga3
Chol_Lsr
E1_Cd93
E1_Nrp1
E1_Itga9
E2_Lrp5
HSC_Egfr
HSC_Lrp5
HSC_Itga3
HSC_Lsr
HSC_Itga1
HSC_Itga9
HSC_Lrp1
HSC_Alcam
Hep_Egfr
Hep_Lrp1
Hep_Nrp1
Hep_Cd36
MC1_Acvrl1
MC1_Lrp1
MC1_Tlr4
MC2_Acvrl1
Cd52
MC2_Cmklr1
Jmjd6
Slc16a1
Egfr
Robo1
Itgb6
Igf2r
Podxl
B
Btc in mouse liver
GSE197884
GSE222576
Gene expression (CPM)
0.1000
chow
HFHS
Gene expression
**
ns
*
Control
Ccl4-2w
Ccl4-4w
Ccl4-6w
Ccl4-8w
Ccl4-10w

Figure EV4.

Figure EV5. Potential mechanism of BTC regulation by the ω3 PUFA.

A The representation of network model and network interrogation to identify genes (nodes) top ranked according to Bipartite betweenness centrality (BiBC) and degree which potentially mediate effects of ω3 PUFA lipids on BTC-dependent gene expression profile. The legend for nodes is shown.

B The scheme of analysis to identify upstream regulator of Btc. The three modules of analysis with experimental data, network interrogation and identification of transcription regulator for Btc in Cholangiocytes. Identified transcription factors are labeled. Predicted binding site and motif for Foxo3 mediated transcription regulation in mice and human for Btc gene expression in the liver cholangiocytes is shown.

C Foxo3 gene expression *in vivo*. DHA reversed the gene expression significantly in the *in vivo* experimental model both in Preventive & Treatment models (Data are displayed as mean ± SD, $N = 8$ mice/group (preventative study) or $N = 5$ or 6 mice per group (treatment study); Ordinary One-way ANOVA, multiple comparisons test of reference diet (chow, RD), western diet + DHA (WD + DHA), western diet + EPA (WD + EPA) each with western diet + olive oil (WD + O), ns (not significant), $^*P < 0.05$, $^{***}P < 0.005$).

 © 2023 The Authors

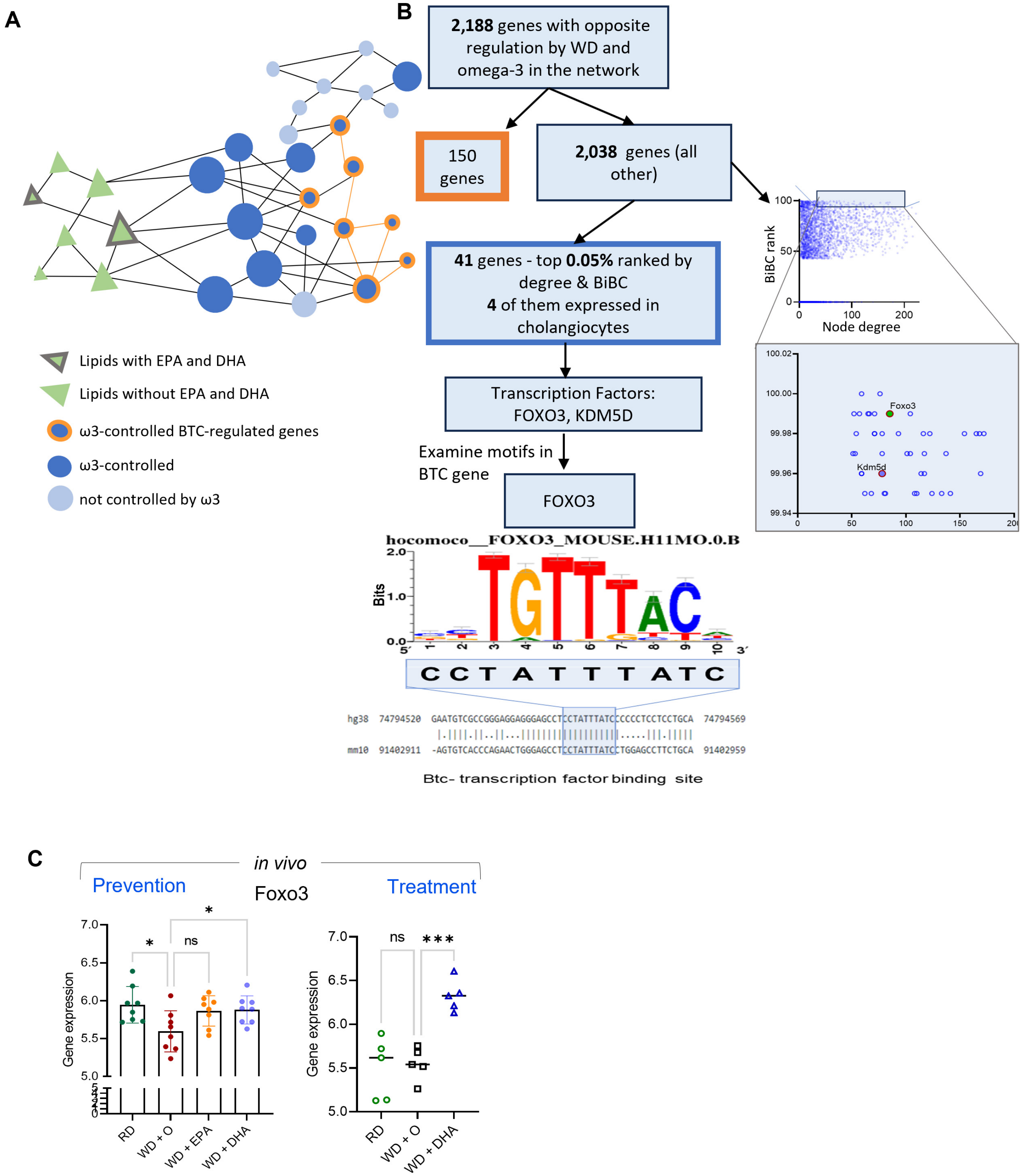
A
Lipids with EPA and DHA
Lipids without EPA and DHA
ω3-controlled BTC-regulated genes
ω3-controlled
not controlled by ω3
B
2,188 genes with opposite regulation by WD and omega-3 in the network
150 genes
2,038 genes (all other)
41 genes - top 0.05% ranked by degree & BiBC
4 of them expressed in cholangiocytes
Transcription Factors: FOXO3, KDM5D
Examine motifs in BTC gene
FOXO3
hocomoco__FOXO3_MOUSE.H11MO.0.B
Bits
C C T A T T T A T C
Btc- transcription factor binding site
BiBC rank
Node degree
Foxo3
Kdm5d
C
in vivo
Prevention
Treatment
Foxo3
Gene expression
RD
WD + O
WD + EPA
WD + DHA
ns
*
***

Figure EV5.

Table of Contents **Page number**

Appendix Figure S1	2
Appendix Figure S2	4
Appendix Table S1	7

Appendix Figure S1

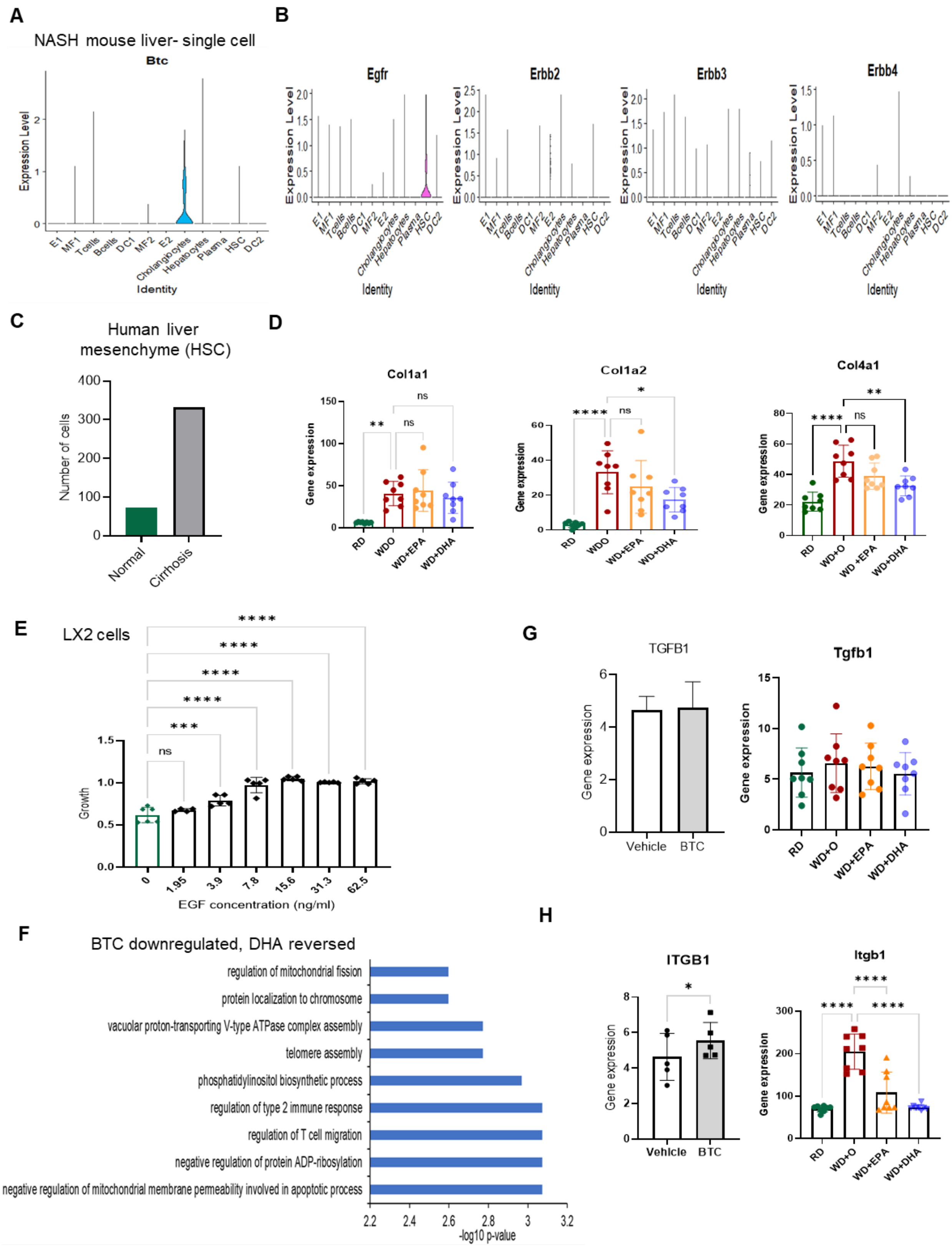

Appendix Figure S1. A. The expression of Btc in Mouse NASH liver single cell RNA sequence data shown here with the maximum expression in liver cholangiocytes (MF1-KC; MF2 -NAM).

B. The expression of Egfr and other Erbbs in the NASH mouse model single cell RNA sequence data.
C. The number of Mesenchymal cells (Hepatic stellate cells) in Human liver samples with enrichment in Cirrhosis with more than 3-fold increase in numbers than normal liver samples.
D. The different collagen genes expression in the NASH preventive model shown is in bar graphs colored by treatment effects (Data are mean +/- SD, N=8 mice/treatment group. Ordinary One-way ANOVA, with multiple comparisons test with WD+O, ns (not significant), *p<0.05, ** p<0.001, ****p<0.0001).
E. The growth of LX2 cells in response to EGF in a dose dependent manner shown in the bar graph. (Ordinary One-way ANOVA, with multiple comparisons test with Control, ns (not significant), *** p<0.005, ****p<0.0001)
F. The gene enrichment analysis shown in a bar plot, regulation of mitochondrial fission and mitochondrial membrane permeability mediated apoptotic pathway are significantly down regulated by BTC treatment in LX2 cells while they are reversed by DHA treatment in the *in vivo* mouse model.
G. TGFB1 expression in LX2 cells treated with BTC (grey) (20 ng/ml; N=5 experiments, paired, one-sided t-test, ns (not significant) and in the NASH preventive model is shown in bar graphs colored by treatment effects (Ordinary One-way ANOVA, with multiple comparisons test with WD+O, ns (not significant).
H. ITGB1 expression in LX2 cells treated with BTC (grey) (20 ng/ml; N=5 experiments, paired, one-sided t-test, *p<0.05) and in the NASH preventive model is shown in bar graphs colored by treatment effects (Data are mean +/- SD, N=8 mice/treatment group. Ordinary One-way ANOVA, with multiple comparisons test with WD+O, ns (not significant), *p<0.05, ** p<0.001, *** p<0.005, ****p<0.0001).

Appendix Figure S2

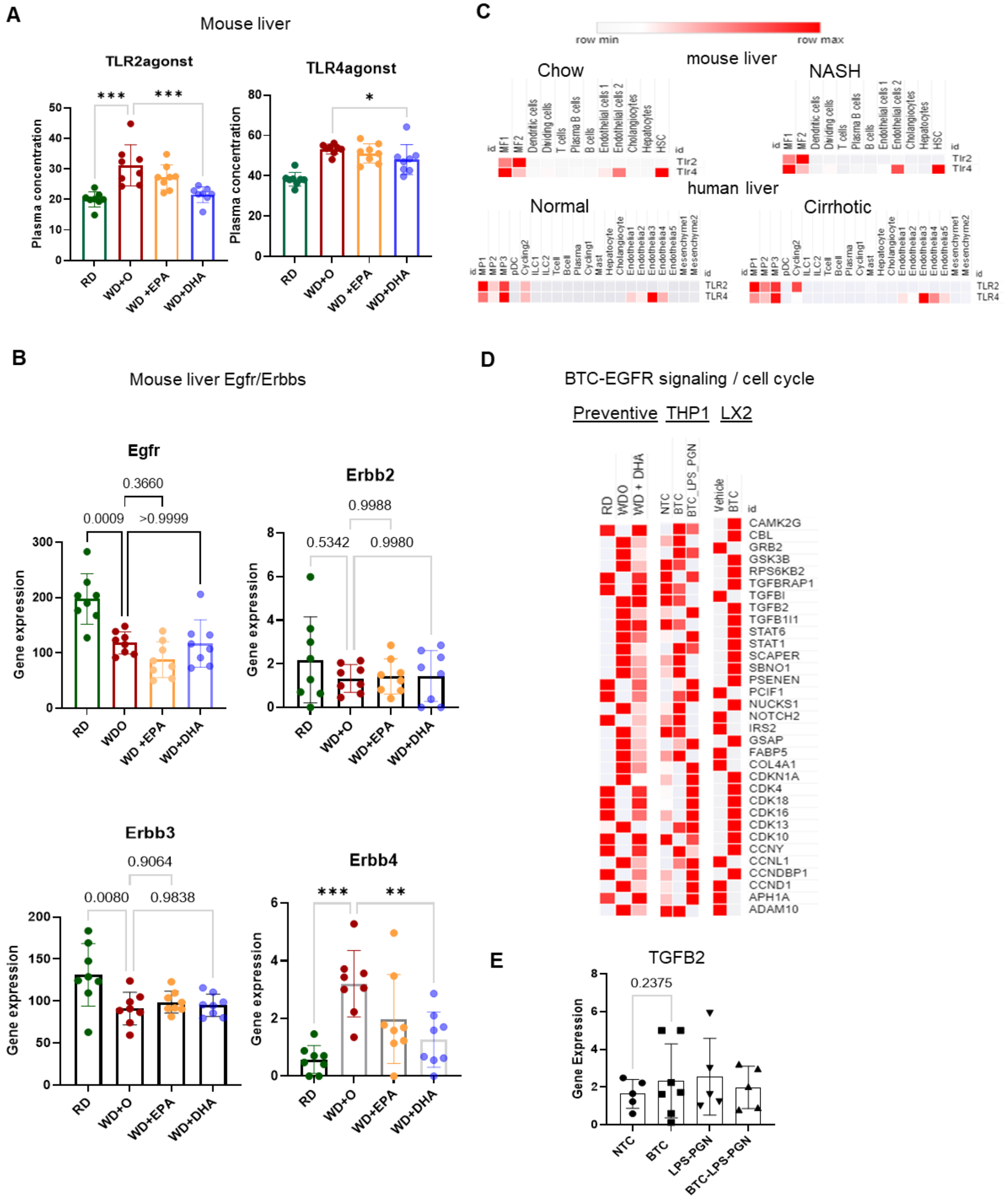

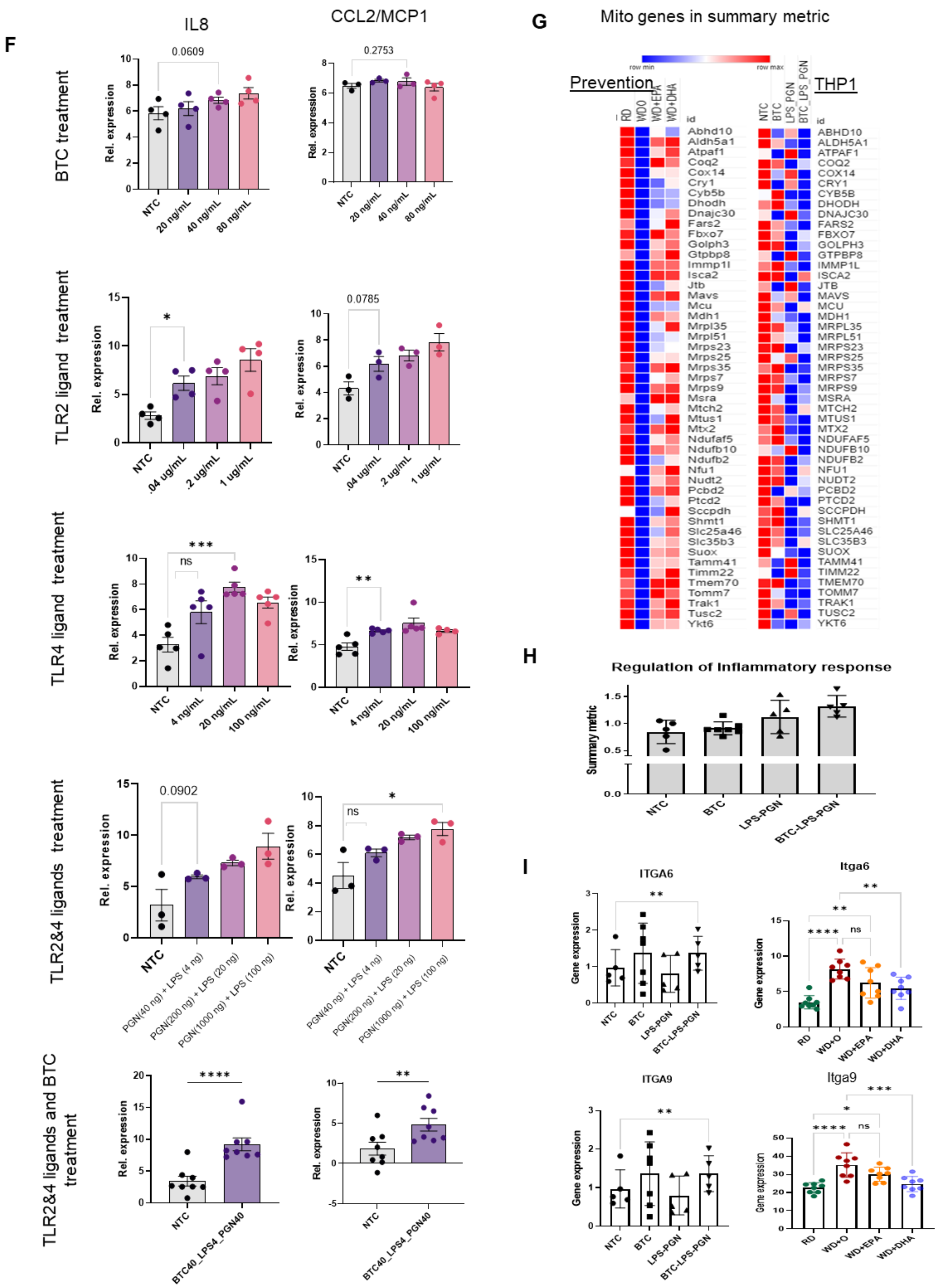
F
IL8
CCL2/MCP1
BTC treatment
Rel. expression
0.0609
0.2753
NTC
20 ng/mL
40 ng/mL
80 ng/mL
TLR2 ligand treatment
0.0785
.04 ug/mL
.2 ug/mL
1 ug/mL
TLR4 ligand treatment
ns
4 ng/mL
20 ng/mL
100 ng/mL
TLR2&4 ligands treatment
0.0902
PGN(40 ng) + LPS (4 ng)
PGN(200 ng) + LPS (20 ng)
PGN(1000 ng) + LPS (100 ng)
TLR2&4 ligands and BTC treatment
BTC40_LPS4_PGN40
G
Mito genes in summary metric
row min
row max
Prevention
THP1
RD
WDO
WD+EPA
WD+DHA
NTC
BTC
LPS_PGN
BTC_LPS_PGN
id
Abhd10
Aldh5a1
Atpaf1
Coq2
Cox14
Cry1
Cyb5b
Dhodh
Dnajc30
Fars2
Fbxo7
Golph3
Gtpbp8
Immp1l
Isca2
Jtb
Mavs
Mcu
Mdh1
Mrpl35
Mrpl51
Mrps23
Mrps25
Mrps35
Mrps7
Mrps9
Msra
Mtch2
Mtus1
Mtx2
Ndufaf5
Ndufb10
Ndufb2
Nfu1
Nudt2
Pcbd2
Ptcd2
Sccpdh
Shmt1
Slc25a46
Slc35b3
Suox
Tamm41
Timm22
Tmem70
Tomm7
Trak1
Tusc2
Ykt6
ABHD10
ALDH5A1
ATPAF1
COQ2
COX14
CRY1
CYB5B
DHODH
DNAJC30
FARS2
FBXO7
GOLPH3
GTPBP8
IMMP1L
ISCA2
JTB
MAVS
MCU
MDH1
MRPL35
MRPL51
MRPS23
MRPS25
MRPS35
MRPS7
MRPS9
MSRA
MTCH2
MTUS1
MTX2
NDUFAF5
NDUFB10
NDUFB2
NFU1
NUDT2
PCBD2
PTCD2
SCCPDH
SHMT1
SLC25A46
SLC35B3
SUOX
TAMM41
TIMM22
TMEM70
TOMM7
TRAK1
TUSC2
YKT6
H
Regulation of Inflammatory response
Summary metric
LPS-PGN
BTC-LPS-PGN
I
ITGA6
Itga6
ITGA9
Itga9
Gene expression
RD
WD+O
WD+EPA
WD+DHA

Appendix Figure S2. A. Expression of TLR2/4 agonists in the NASH preventive model is shown in bar graphs colored by treatment effects (Data are mean +/- SD, N=8 mice/treatment group). (Ordinary One-way ANOVA, with multiple comparisons test with WD+O, *p<0.05, *** p<0.005)

B. The Egfr/Erbbs expression in the NASH preventive model is shown in bar graphs colored by treatment effects (Data are mean +/- SD, N=8 mice/treatment group). (Ordinary One-way ANOVA, with multiple comparisons test with WD+O, ns (not significant), ** p<0.001, *** p<0.005)
C. The mouse and human liver (with or without NASH/Cirrhosis) cluster wise average TLR2/4 gene expression from the single cell RNA sequence data. The color scale is indicated from high expression of the genes in red to low in white.
D. The gene expression heatmap from BTC/TLR2/4 ligands treated THP-1 and LX2 cells shows genes involved in EGFR pathway and cell cycle pathway that are induced. These set of genes were revered by DHA in the mouse NASH preventive model. The color scale in heatmap is indicated from high expression of the genes in red to low in white.
E. Normalized TGFB2 expression in THP-1 cells treated with BTC and or TLR2/4 ligands (5 separate experiments, paired, one-sided t-test, ns (not significant), *p<0.05).
F. The dose-response standardization experiments with series of concentrations of BTC, TLR2/4 ligands on THP-1 cells before identifying the lowest concentration for combination of all three together. The well-known cytokines were chosen as markers of gene expression with treatments (IL6 and CCL2; 5-8 experiments, paired, one-sided t-test, ns (not significant), *p<0.05, ** p<0.001, *** p<0.005, ****p<0.0001).
G. A heatmap from gene list derived from the enrichment analysis of mitochondria, in summary metric for BTC/TLR2/4 ligand treatment effects in THP1 cells revered by DHA treatment *in vivo* model. The color scale is indicated from high expression of the genes in red to low in blue.
H. A summary metric bar graph for BTC/TLR2/4 ligand treatment effects in THP1 cells revered by DHA treatment in vivo model from the enrichment analysis.
I. The integrin (ITGA6 and ITGA9) expression in the NASH preventive model is shown in bar graphs colored by treatment effects (Data are mean +/- SD, N=8 mice/treatment group). (Ordinary One-way ANOVA, with multiple comparisons test with WD+O, ns (not significant), *p<0.05, ** p<0.001, *** p<0.005, ****p<0.0001) and in THP-1 cells treated with BTC and or TLR2/4 ligands (N=5 experiments, paired, one-sided t-test, ns (not significant), *p<0.05).

Appendix Table S1

Legend: THP-1 cells response to TLR and BTC stimulation was assessed by qRT-PCR, the primer sequences as follows.

Gene	Forward	Reverse	Organism
IL8	ACTCCAAACCTTTCCACCCCA	CCCTCTGCACCCAGTTTTCCT	Human
TMEM59	GCTTCATAACCTCTTCATGGAC	GGATTTCTGGCTTAGACTGGA	Human
CCL2/MCP-1	CCCCAGTCACCTGCTGTTAT	AGATCTCCTTGGCCACAATG	Human
