# Supplementary material for: Multi‐omic network analysis identified betacellulin as a novel target of omega‐3 fatty acid attenuation of western diet‐induced nonalcoholic steatohepatitis
Source: EMBO Mol Med. 2023 Oct 20;15(11):e18367. doi: 10.15252/emmm.202318367 (PMC10630881; doi:10.15252/emmm.202318367)
Supplement: Supplementary file 12 [file EMMM-15-e18367-s010.pdf]

EMBO Molecular Medicine

Multi-omic network analysis identified betacellulin as a novel target of omega- 3 fatty acid attenuation of western diet-induced nonalcoholic steatohepatitis

Jyothi Padiadpu, Manuel Garcia-Jaramillo, Nolan Newman, jacob Pederson, Richard Rodrigues, Zhipeng Li, Sehajvir Singh, Philip Monnier, Giorgio Trinchieri, Kevin Brown, Amiran Dzutsev, Natalia Shulzhenko, Donald Jump, and Andrey Morgun
DOI: 10.15252/emmm.202318367

Corresponding authors: Natalia Shulzhenko (natalia.shulzhenko@oregonstate.edu) , Donald Jump (Donald.Jump@oregonstate.edu), Andrey Morgun (andriy.morgun@oregonstate.edu)

Review Timeline:

Transfer from Review Commons:	19th Jul 23
Editorial Decision:	29th Aug 23
Revision Received:	19th Sep 23
Accepted:	21st Sep 23

Editor: Zeljko Durdevic

Transaction Report:

This manuscript was transferred to EMBO Molecular Medicine following peer review at Review Commons.

(Note: With the exception of the correction of typographical or spelling errors that could be a source of ambiguity, letters and reports are not edited. Depending on transfer agreements, referee reports obtained elsewhere may or may not be included in this compilation. Referee reports are anonymous unless the Referee chooses to sign their reports.)

Review #1

1. Evidence, reproducibility and clarity:

Evidence, reproducibility and clarity (Required)

This work by Padiadpu and colleagues investigate the mechanism by which pufa of the n-3 series (mostly DHA) may influence NAFLD progression using systems biology analysis and multiple omics analysis.

The work is interesting and may provide a novel view of the topic.

However, there are a number of issues the authors may wish to consider in order to improve their manuscript.

Major issues:

Clarity:

Since the authors refer to previously published experiments they must refer to this work in the figure legends and improve the clarity of such legends.
Here are a list of issues that must be fixed:
Fig.1 : Firts panel is not clear. What does the table tell the reader?
What are the effects of the different diets on NAFLD? I understand that the results are published elsewhere, but the authors must provide information regarding the NAFLD/ NASH scores.
Fig.4: Why is there sometimes a DHA diet, sometimes DHA and EPA.
Legend is not clear. What does WD + Mean? I guess it is olive oil... But the legend must be improved.

One issue the authors may consider trying to fix is the specificity of the effect of DHA on BTC. Is it really specific? It seems to me that EPA has more or less the same effect. If the effect is DHA-specific, than make this clearer through the text. In this current version of the manusript, the authors alternatively use the term DHA or W3.
Related to this issue, it would be nice to know what the composition of the WD is? More specifically, it would be important to know whether it might be W3 deficient.

Another issue the authors may wish to investigate is the relationship between W3 consumption and BTC expression in studies performed by other labs (if available on Gene expression omnibus?).

Finally, a key issue would be to identify the mechanism by which DHA inhibits BTC expression? How does this happen? could such inhibition be induced by other fatty acids of the W3 series? I understand that this is not easy to address but it would significantly strengthen the manuscript.
Moreover, it might be possible to identify the set of genes highly co-regulated with BTC

expression and to investigate the possible transcription factors at play in the control of such gene set.

Minor: the authors use the term "beneficial" transcriptome alterations by DHA. I do not think it is correct to use "beneficial".

2. Significance:

Significance (Required)

Strenght:

This paper uses new approaches to investigate the relationship between W3 consumption and liver gene expression and its relevance to chronic metabolic liver diseases.
The experiments and data set used to perform systems biology are from and excellent lab (the authors lab) who has published a lot of important and reproducible discoveries in the field of regulation of gene expression by dietary fatty acids.

Limitation:

The mechanistic link between DHA consumption and BTC expression is not very clear. The specificity of this effect could also be tested (DHA vs other W3 and/or W6).

The work has high translational relevance in medicine / hepatology / metabolism.

I am not a qualified reviewer to assess the systems biology that has been done.

3. How much time do you estimate the authors will need to complete the suggested revisions:

Estimated time to Complete Revisions (Required)

(Decision Recommendation)

Cannot tell / Not applicable

4. *Review Commons* values the work of reviewers and encourages them to get credit for their work. Select 'Yes' below to register your reviewing activity at [Web of Science Reviewer Recognition Service]() (formerly Publons); note that

the content of your review will not be visible on Web of Science.

Web of Science Reviewer Recognition

Yes

Review #2

1. Evidence, reproducibility and clarity:

Evidence, reproducibility and clarity (Required)

The authors files a manuscript describing the impact of the suppression of betacellulin as a key mechanism to counteract fibrosis and inflammation in NASH by modulating fatty acids in WD-fed mice.

Major Comments:

- (i) No histological analysis was presented and indeed this is of clinical relevance for NASH since diagnosis is still based on biopsy.
- (ii) Human comparative analysis: is done with HCC not with NASH patients. This cancer-related dataset is most likely obtained from different etiologies. I would suggest comparing these mouse datasets with GSE48452 (human NAFLD-NASH spectra).
- (iii) to compare the inflammation and fibrosis (also lipid metabolism), one can compare these mouse datasets with GSE222576 and cite this preprint (https://doi.org/10.21203/rs.3.rs-2009380/v1)

Minor comments:

- (i) The heatmap in Figure 1B and another heatmap should show all mice not the average to see the variability

2. Significance:

Significance (Required)

The authors files a manuscript describing the impact of the suppression of betacellulin as a key mechanism to counteract fibrosis and inflammation in NASH by modulating fatty acids. This is

well designed experiment and the results are of interest to hepatologists and should be indeed published after consideration of the following points

Strength is multiOMICs approach

Weakness is human applicability

3. How much time do you estimate the authors will need to complete the suggested revisions:

Estimated time to Complete Revisions (Required)

(Decision Recommendation)

Between 3 and 6 months

4. *Review Commons* values the work of reviewers and encourages them to get credit for their work. Select 'Yes' below to register your reviewing activity at Web of Science Reviewer Recognition Service (formerly Publons); note that the content of your review will not be visible on Web of Science.

Web of Science Reviewer Recognition

Yes

Full Revision

Manuscript number: RC -2023-01904
Corresponding author(s): Andrey Morgun

[Please use this template only if the submitted manuscript should be considered by the affiliate journal as a full revision in response to the points raised by the reviewers.

If you wish to submit a preliminary revision with a revision plan, please use our "Revision Plan" template. ***It is important to use the appropriate template to clearly inform the editors of your intentions.****]*

1. General Statements [optional]

This section is optional. Insert here any general statements you wish to make about the goal of the study or about the reviews.

We would like to thank reviewers for their insightful comments.

Overall, there were two major concerns/suggestions:

1- Applicability to humans of the increase of BTC in non-alcoholic steatohepatitis (NASH) and mechanisms of downregulation of BTC by omega-3.
We now analyzed **3** additional human gene expression datasets and show that BTC not only is increased in human NASH (as we have already shown for liver cancer meta-analysis), but is also decreased in livers of patients who received omega-3.

2- One of the reviewers suggested investigating a potential mechanism of how BTC is regulated by omega3 fatty acids.
Although a complete answer to this question would require entirely new studies to be done, we still performed additional investigation that was possible within a reasonable timeframe. We found that transcription factor FOXO3 (well-known inhibitor of carcinogenesis) is a highly probable mediator of the DHA inhibitory effect on BTC.

See all details of items 1 and 2 as well as answers to other (less critical concerns) below in blue after each specific question.

2. Point-by-point description of the revisions

This section is mandatory. Please insert a point-by-point reply describing the revisions that were already carried out and included in the transferred manuscript.

Reviewer #1 (Evidence, reproducibility and clarity (Required)):
This work by Padiadpu and colleagues investigate the mechanism by which pufa of the n-3 series (mostly DHA) may influence NAFLD progression using systems biology analysis and

Review COMMONS

multiple omics analysis. The work is interesting and may provide a novel view of the topic. However, there are a number of issues the authors may wish to consider in order to improve their manuscript.

Major issues: Clarity: Since the authors refer to previously published experiments, they must refer to this work in the figure legends and improve the clarity of such legends. Here are a list of issues that must be fixed:

Fig.1: First panel is not clear. What does the table tell the reader? What are the effects of the different diets on NAFLD?

All the transcriptomic data are newly generated from the samples of previously published studies. The table shows the number of features changed by DHA and/or EPA in each of the -omics and phenotypic data used in the analysis.

I understand that the results are published elsewhere, but the authors must provide information regarding the NAFLD/ NASH scores.

We now added a supplementary table 1a showing the scores.

Fig.4: Why is there sometimes a DHA diet, sometimes DHA and EPA. Legend is not clear. What does WD + Mean? I guess it is olive oil... But the legend must be improved.

We added details in the legend for more clarity. Specifically, WD+O means WD + olive oil added as a control for WD+DHA, WD+EPA. As described in the 2nd paragraph of results, when both EPA and DHA had a similar and significant effects in reversing WD effect, it was defined as "EPA&DHA category" of parameters. When only WD+DHA or WD+EPA were significantly changed vs WD+O, those were assigned as "DHA category" or "EPA category", respectively.

One issue the authors may consider trying to fix is the specificity of the effect of DHA on BTC. Is it really specific? It seems to me that EPA has more or less the same effect. If the effect is DHA-specific, than make this clearer through the text.

Although BTC expression was reduced by both DHA and EPA comparing to WD, DHA had a statistically significant more potent effect than EPA (Fig. 3D).

In this current version of the manuscript, the authors alternatively use the term DHA or W3. Related to this issue, it would be nice to know what the composition of the WD is? More specifically, it would be important to know whether it might be W3 deficient.

WD does not contain DHA or EPA. We added this information in M&M and refer to our previous publications where all details of diet composition are provided (https://pubmed.ncbi.nlm.nih.gov/23303872/).

Another issue the authors may wish to investigate is the relationship between W3 consumption and BTC expression in studies performed by other labs (if available on Gene expression omnibus?).

Thanks for the suggestion. We used publicly available data of human and mouse studies that showed significant increase in liver BTC gene expression in NASH in multiple datasets while a human trial with Omega 3 treatment for one year showed its significant reduction (Figures 3F, S3G).

Finally, a key issue would be to identify the mechanism by which DHA inhibits BTC expression? How does this happen? could such inhibition be induced by other fatty acids of the W3 series? I understand that this is not easy to address but it would significantly strengthen the manuscript.

Review COMMONS

Thanks to your question we investigated and found at least one of potential mechanisms contributing to how “DHA inhibits BTC expression”. See details in the answer to next question. As for “other fatty acids” while we agree this is important question, it is outside of the scope of the current study but will be investigated in future studies.

Moreover, it might be possible to identify the set of genes highly co-regulated with BTC expression and to investigate the possible transcription factors at play in the control of such gene set.
We really appreciate this question as our efforts in this direction provided one potential mechanism. A direct screen of transcription factor (TF) motifs in genes co-regulated with BTC did not provide any clear results. Therefore, we implemented a combination of network analysis and screen for motifs in BTC gene with the in vivo and in vitro treatment results and found FOXO3 as a candidate TF regulated by DHA upstream of BTC.
See details of the analysis and results in a new Supplementary Figure S6 and corresponding text located at the end of the results.

Minor: the authors use the term "beneficial" transcriptome alterations by DHA.
I do not think it is correct to use "beneficial".
We agree and removed the word "beneficial”.

Reviewer #1 (Significance (Required)):
Strength: This paper uses new approaches to investigate the relationship between W3 consumption and liver gene expression and its relevance to chronic metabolic liver diseases.

The experiments and data set used to perform systems biology are from an excellent lab (the authors lab) who has published a lot of important and reproducible discoveries in the field of regulation of gene expression by dietary fatty acids.

Limitation: The mechanistic link between DHA consumption and BTC expression is not very clear. The specificity of this effect could also be tested (DHA vs other W3 and/or W6).
Although BTC expression was reduced by both DHA and EPA comparing to WD, DHA had a significantly more potent effect than EPA (Fig. 3D). Other omega fatty acids were not tested but it can be done in future studies.

The work has high translational relevance in medicine / hepatology / metabolism.
I am not a qualified reviewer to assess the systems biology that has been done.

Reviewer #2 (Evidence, reproducibility and clarity (Required)):
The authors files a manuscript describing the impact of the suppression of betacellulin as a key mechanism to counteract fibrosis and inflammation in NASH by modulating fatty acids in WD-fed mice.

Major Comments:
(i) No histological analysis was presented and indeed this is of clinical relevance for NASH since diagnosis is still based on biopsy.
Histological evaluation was presented in the originally published papers (PMID: 28422962, 23303872) and is now provided in Supplementary table S1a.

(ii) Human comparative analysis: is done with HCC not with NASH patients.
This cancer-related dataset is most likely obtained from different etiologies.
I would suggest comparing these mouse datasets with GSE48452 (human NAFLD-NASH spectra).
Thanks for this important question. We now analyzed available human data of NASH and show significant increase of BTC expression in two datasets while a human trial with omega-3 treatment for one year showed its significant reduction of BTC expression (Figure 3F) resembling our observations in mice.

(iii) to compare the inflammation and fibrosis (also lipid metabolism), one can compare these mouse datasets with GSE222576 and cite this preprint (https://doi.org/10.21203/rs.3.rs-2009380/v1)

Using the suggested dataset of a chemically induced liver fibrosis, we first observed that Btc gene expression was significantly increased over 10 weeks of the model and now included this result in Fig. S3G.
We also queried the 66 genes from the network modules described by the authors for their changes in our NASH model. We observed that 28 genes were differentially expressed in NASH with 14 of them belonging to the module that authors named as “Pathways in Cancer”. Other genes were from the lipid metabolism (4 genes), immunity (2) and inflammation (2 genes).
In addition, we observed that several genes regulated by omega-3 and changed in the chemical model contained other inflammatory genes such as classical macrophage genes (Mmp12, Lgals3, Cd68, Trem2), fibrosis (Col4a1, Col27a1, Itga2b, Itga8) and lipid metabolism (Scd2, Lpl, Soat1).
Of note, the preprint has been published and we now included the corresponding citation.

Minor comments:
(i) The heatmap in Figure 1B and another heatmap should show all mice not the average to see the variability
The supplementary figure with all the individual mouse data as another heatmap is added to show the variability and similarity (Figure S1D).

Reviewer #2 (Significance (Required)): The authors files a manuscript describing the impact of the suppression of betacellulin as a key mechanism to counteract fibrosis and inflammation in NASH by modulating fatty acids.

This is well designed experiment, and the results are of interest to hepatologists and should be indeed published after consideration of the following points

Strength is multiOMICs approach.

Weakness is human applicability.
We improved human applicability by investigating 3 additional human datasets of NASH (Fig. 3F) and finding consistent changes in BTC expression closely resembling our observations in mouse NASH model.

29th Aug 2023

Dear Dr. Morgun,

Thank you for the submission of your revised manuscript to EMBO Molecular Medicine, and please accept my apologies for the delay in getting back to you. I am pleased to inform you that we will be able to accept your manuscript pending the following final amendments:

1) Your manuscript is currently being reviewed by our data editors. I will send you the manuscript with the track changes suggested by our data editors as soon as all the checks are completed.
2) We note that you currently have together with you, a total of 3 co-corresponding authors. Is that correct? Do you confirm equal contribution of these 3 people, able to take full responsibility for the paper and its content? While there is no limit per se to the number of co-corresponding authors, 3 is rare, and may not reflect as intended to the community.
3) Please place individual sections of the manuscript in the following order: Title page - Abstract & Keywords - Introduction - Results - Discussion - Materials & Methods - Data Availability - Acknowledgements - Disclosure Statement & Competing Interests - References - Figure Legends - Expanded View Figure Legends.
4) Figures: Please remove all figures from main manuscript file and leave only main figure legends placed after the references. There are 7 suppl. figures. Up to 5 can be made EV figures. Each figure will need to fit onto one page. The legends should stay in the manuscript, with the heading Expanded View Figures Legends, and placed after the main figure legends. The remaining two can be compiled in an appendix file, with the legends under each figure, and renamed Appendix Figure S1 and S2. The appendix should be uploaded in PDF format and needs a table of content with page numbers. Please make sure to update the callouts of all figures in the main manuscript text. Main figures and EV figures should be uploaded as individual, high-resolution files. Please check "Author Guidelines" for more information:
https://www.embopress.org/page/journal/17574684/authorguide#figureformat
5) Tables: Please uploaded all tables as one .xsl file per table and rename them to Dataset EV1-7 (Dataset EV1 should consist of two tabs with Dataset EV1a and Dataset EV1b). Each dataset will need its legend removed from the manuscript and added to the corresponding file in a separate tab. Please update their callouts in main manuscript text. The table with primers in M&M section should be moved to "Appendix", named Appendix Table S1 with a legend and called out in M&M.
6) Please check "Author Checklist" carefully and complete all relevant questions. Currently information about studies involving experimental animals is missing.
7) In the main manuscript file, please do the following:
- Reduce keywords to max. 5.
- Please rename "Competing Interest" to "Disclosure Statement & Competing Interests". We updated our journal's competing interests policy in January 2022 and request authors to consider both actual and perceived competing interests. Please review the policy https://www.embopress.org/competing-interests and update your competing interests if necessary.
- Author contributions: Please remove it from the manuscript and specify author contributions in our submission system. CRediT has replaced the traditional author contributions section because it offers a systematic machine-readable author contributions format that allows for more effective research assessment. You are encouraged to use the free text boxes beneath each contributing author's name to add specific details on the author's contribution. More information is available in our guide to authors:
https://www.embopress.org/page/journal/17574684/authorguide#authorshipguidelines
- Data availability: The metabolomic dataset is not publicly available. Please be aware that all deposited datasets should be freely accessible upon publication. Also, please use the following format to report the accession number of your data:

The datasets produced in this study are available in the following databases:
[data type]: [full name of the resource] [accession number/identifier] ([doi or URL or identifiers.org/DATABASE:ACCESSION])

Please check "Author Guidelines" for more information.
https://www.embopress.org/page/journal/17574684/authorguide#availabilityofpublishedmaterial
- Correct the reference citation in the reference list. Where there are more than 10 authors on a paper, 10 will be listed, followed by "et al.". Please check "Author Guidelines" for more information.
https://www.embopress.org/page/journal/17574684/authorguide#referencesformat
8) The Paper Explained: Please provide "The Paper Explained" and add it to the main manuscript text. Please check "Author Guidelines" for more information. https://www.embopress.org/page/journal/17574684/authorguide#researcharticleguide
9) Synopsis:
- Synopsis image: Please remove it from the manuscript and upload it as a high-resolution jpeg file 550 px-wide x (250-400)-px high.
- Synopsis text: Please provide a short standfirst (maximum of 300 characters, including space), limit the bullet points to max. 5 and upload it as a separate .doc file. Please write the bullet points to summarise the key NEW findings. They should be designed to be complementary to the abstract - i.e. not repeat the same text. We encourage inclusion of key acronyms and quantitative information (maximum of 30 words / bullet point). Please use the passive voice.
- Please check your synopsis text and image before submission with your revised manuscript. Please be aware that in the proof

stage minor corrections only are allowed (e.g., typos).
10) For more information: This space should be used to list relevant web links for further consultation by our readers. Could you identify some relevant ones and provide such information as well? Some examples are patient associations, relevant databases, OMIM/proteins/genes links, author's websites, etc...
11) As part of the EMBO Publications transparent editorial process initiative (see our Editorial at http://embomolmed.embopress.org/content/2/9/329), EMBO Molecular Medicine will publish online a Review Process File (RPF) to accompany accepted manuscripts. This file will be published in conjunction with your paper and will include the anonymous referee reports, your point-by-point response and all pertinent correspondence relating to the manuscript. Let us know whether you agree with the publication of the RPF and as here, if you want to remove or not any figures from it prior to publication. Please note that the Authors checklist will be published at the end of the RPF.
12) Please provide a point-by-point letter INCLUDING my comments as well as the reviewer's reports and your detailed responses (as Word file).

I look forward to reading a new revised version of your manuscript as soon as possible.

Yours sincerely,

Zeljko Durdevic

Zeljko Durdevic
Editor
EMBO Molecular Medicine

*** Instructions to submit your revised manuscript ***

*** PLEASE NOTE *** As part of the EMBO Publications transparent editorial process initiative (see our Editorial at https://www.embopress.org/doi/pdf/10.1002/emmm.201000094), EMBO Molecular Medicine will publish online a Review Process File to accompany accepted manuscripts.

In the event of acceptance, this file will be published in conjunction with your paper and will include the anonymous referee reports, your point-by-point response and all pertinent correspondence relating to the manuscript. If you do NOT want this file to be published, please inform the editorial office at contact@embomolmed.org.

When submitting your revised manuscript, please include:

1) a .docx formatted version of the manuscript text (including Figure legends and tables)

2) Separate figure files*

3) supplemental information as Expanded View and/or Appendix. Please carefully check the authors guidelines for formatting Expanded view and Appendix figures and tables at
https://www.embopress.org/page/journal/17574684/authorguide#expandedview

4) a letter INCLUDING the reviewer's reports and your detailed responses to their comments (as Word file).

5) The paper explained: EMBO Molecular Medicine articles are accompanied by a summary of the articles to emphasize the major findings in the paper and their medical implications for the non-specialist reader. Please provide a draft summary of your article highlighting
- the medical issue you are addressing,
- the results obtained and
- their clinical impact.
This may be edited to ensure that readers understand the significance and context of the research.
Please refer to any of our published articles for an example.

6) For more information: There is space at the end of each article to list relevant web links for further consultation by our readers. Could you identify some relevant ones and provide such information as well? Some examples are patient associations, relevant databases, OMIM/proteins/genes links, author's websites, etc...

7) Author contributions: the contribution of every author must be detailed in a separate section.

8) EMBO Molecular Medicine now requires a complete author checklist (https://www.embopress.org/page/journal/17574684/authorguide) to be submitted with all revised manuscripts. Please use the checklist as guideline for the sort of information we need WITHIN the manuscript. The checklist should only be filled with page numbers were the information can be found. This is particularly important for animal reporting, antibody dilutions (missing) and exact values and n that should be indicted instead of a range.

9) Every published paper now includes a 'Synopsis' to further enhance discoverability. Synopses are displayed on the journal webpage and are freely accessible to all readers. They include a short stand first (maximum of 300 characters, including space) as well as 2-5 one sentence bullet points that summarise the paper. Please write the bullet points to summarise the key NEW findings. They should be designed to be complementary to the abstract - i.e. not repeat the same text. We encourage inclusion of key acronyms and quantitative information (maximum of 30 words / bullet point). Please use the passive voice. Please attach these in a separate file or send them by email, we will incorporate them accordingly.

You are also welcome to suggest a striking image or visual abstract to illustrate your article. If you do please provide a jpeg file 550 px-wide x 400-px high.

10) A Conflict of Interest statement should be provided in the main text

11) Please note that we now mandate that all corresponding authors list an ORCID digital identifier. This takes <90 seconds to complete. We encourage all authors to supply an ORCID identifier, which will be linked to their name for unambiguous name identification.

Currently, our records indicate that the ORCID for your account is 0000-0002-8924-6108.

Please click the link below to modify this ORCID:
Link Not Available

12) The system will prompt you to fill in your funding and payment information. This will allow Wiley to send you a quote for the article processing charge (APC) in case of acceptance. This quote takes into account any reduction or fee waivers that you may be eligible for. Authors do not need to pay any fees before their manuscript is accepted and transferred to our publisher.

*Additional important information regarding Figures

Each figure should be given in a separate file and should have the following resolution:
Graphs 800-1,200 DPI
Photos 400-800 DPI
Colour (only CMYK) 300-400 DPI"

Figures are not edited by the production team. All lettering should be the same size and style; figure panels should be indicated by capital letters (A, B, C etc). Gridlines are not allowed except for log plots. Figures should be numbered in the order of their appearance in the text with Arabic numerals. Each Figure must have a separate legend and a caption is needed for each panel.

*Additional important information regarding figures and illustrations can be found at https://bit.ly/EMBOPressFigurePreparationGuideline. See also figure legend preparation guidelines: https://www.embopress.org/page/journal/17574684/authorguide#figureformat

***** Reviewer's comments *****

Referee #1 (Comments on Novelty/Model System for Author):

One strength of the manuscript is the number of models used and the use of both mouse and human data analysis.

Referee #1 (Remarks for Author):

The authors have made a very good effort at addressing the comments raised in the first round of review (review in commons). This work is very interesting and innovative in the field of regulation of liver biology by polyunsaturated fatty acids of the n-3 series

***

Rev_Com_number: RC-2023-01904
New_manu_number: EMM-2023-18367
Corr_author: Morgun
Title: Multi-omic network analysis identified betacellulin as a novel target of omega-3 fatty acid attenuation of western diet-induced nonalcoholic steatohepatitis

The authors addressed the minor editorial issues.

21st Sep 2023

Dear Dr. Shulzhenko,

We are pleased to inform you that your manuscript is accepted for publication and is now being sent to our publisher to be included in the next available issue of EMBO Molecular Medicine.

Please read below for additional IMPORTANT information regarding your article, its publication and the production process.

Congratulations on your interesting work,

Zeljko Durdevic

Zeljko Durdevic
Editor
EMBO Molecular Medicine

Follow us on Twitter @EmboMolMed
Sign up for eTOCs at embopress.org/alertsfeeds

*** *** *** IMPORTANT INFORMATION *** *** ***

SPEED OF PUBLICATION
The journal aims for rapid publication of papers, using using the advance online publication "Early View" to expedite the process: A properly copy-edited and formatted version will be published as "Early View" after the proofs have been corrected. Please help the Editors and publisher avoid delays by providing e-mail address(es), telephone and fax numbers at which author(s) can be contacted.

Should you be planning a Press Release on your article, please get in contact with embomolmed@wiley.com as early as possible, in order to coordinate publication and release dates.

LICENSE AND PAYMENT:

All articles published in EMBO Molecular Medicine are fully open access: immediately and freely available to read, download and share.

EMBO Molecular Medicine charges an article processing charge (APC) to cover the publication costs. You, as the corresponding author for this manuscript, should have already received a quote with the article processing fee separately. Please let us know in case this quote has not been received.

Once your article is at Wiley for editorial production you will receive an email from Wiley's Author Services system, which will ask you to log in and will present you with the publication license form for completion. Within the same system the publication fee can be paid by credit card, an invoice, pro forma invoice or purchase order can be requested.

Payment of the publication charge and the signed Open Access Agreement form must be received before the article can be published online.

PROOFS

You will receive the proofs by e-mail approximately 2 weeks after all relevant files have been sent o our Production Office. Please return them within 48 hours and if there should be any problems, please contact the production office at embopressproduction@wiley.com.

Please inform us if there is likely to be any difficulty in reaching you at the above address at that time. Failure to meet our deadlines may result in a delay of publication.

All further communications concerning your paper proofs should quote reference number EMM-2023-18367-V2 and be directed to the production office at embopressproduction@wiley.com.

Thank you,

Zeljko Durdevic
Editor
EMBO Molecular Medicine

***
Rev_Com_number: RC-2023-01904
New_manu_number: EMM-2023-18367-V2
Corr_author: Shulzhenko
Title: Multi-omic network analysis identified betacellulin as a novel target of omega- 3 fatty acid attenuation of western diet-induced nonalcoholic steatohepatitis

EMBO Press Author Checklist

Corresponding Author Name:	
Journal Submitted to:	
Manuscript Number:	

USEFUL LINKS FOR COMPLETING THIS FORM
The EMBO Journal - Author Guidelines
EMBO Reports - Author Guidelines
Molecular Systems Biology - Author Guidelines
EMBO Molecular Medicine - Author Guidelines

Reporting Checklist for Life Science Articles (updated January
This checklist is adapted from Materials Design Analysis Reporting (MDAR) Checklist for Authors. MDAR establishes a minimum set of requirements in transparent reporting in the life sciences (see Statement of Task: 10.31222/osf.io/9sm4x). Please follow the journal's guidelines in preparing your
Please note that a copy of this checklist will be published alongside your article.

Abridged guidelines for figures
1. Data
The data shown in figures should satisfy the following conditions:
- the data were obtained and processed according to the field's best practice and are presented to reflect the results of the experiments in an accurate and unbiased manner.
- ideally, figure panels should include only measurements that are directly comparable to each other and obtained with the same assay.
- plots include clearly labeled error bars for independent experiments and sample sizes. Unless justified, error bars should not be shown for technical
- if n<5, the individual data points from each experiment should be plotted. Any statistical test employed should be justified.
- Source Data should be included to report the data underlying figures according to the guidelines set out in the authorship guidelines on Data

2. Captions
Each figure caption should contain the following information, for each panel where they are relevant:
- a specification of the experimental system investigated (eg cell line, species name).
- the assay(s) and method(s) used to carry out the reported observations and measurements.
- an explicit mention of the biological and chemical entity(ies) that are being measured.
- an explicit mention of the biological and chemical entity(ies) that are altered/varied/perturbed in a controlled manner.
- the exact sample size (n) for each experimental group/condition, given as a number, not a range;
- a description of the sample collection allowing the reader to understand whether the samples represent technical or biological replicates (including how many animals, litters, cultures, etc.).
- a statement of how many times the experiment shown was independently replicated in the laboratory.
- definitions of statistical methods and measures:
  - common tests, such as t-test (please specify whether paired vs. unpaired), simple χ2 tests, Wilcoxon and Mann-Whitney tests, can be unambiguously identified by name only, but more complex techniques should be described in the methods section;
  - are tests one-sided or two-sided?
  - are there adjustments for multiple comparisons?
  - exact statistical test results, e.g., P values = x but not P values < x;
  - definition of 'center values' as median or average;
  - definition of error bars as s.d. or s.e.m.

Please complete ALL of the questions below.
Select "Not Applicable" only when the requested information is not relevant for your study.

Materials

Newly Created Materials	Information included in the manuscript?	In which section is the information available? (Reagents and Tools Table, Materials and Methods, Figures, Data Availability Section)
New materials and reagents need to be available; do any restrictions apply?	Yes	Materials and Methods, Figures, Data avilability Section

Antibodies	Information included in the manuscript?	In which section is the information available? (Reagents and Tools Table, Materials and Methods, Figures, Data Availability Section)
For **antibodies** provide the following information:<br>- Commercial antibodies: RRID (if possible) or supplier name, catalogue number and or/clone number<br>- Non-commercial: RRID or citation	Not Applicable	

DNA and RNA sequences	Information included in the manuscript?	In which section is the information available? (Reagents and Tools Table, Materials and Methods, Figures, Data Availability Section)
**Short novel DNA or RNA including primers, probes**: provide the sequences.	Not Applicable	

Cell materials	Information included in the manuscript?	In which section is the information available? (Reagents and Tools Table, Materials and Methods, Figures, Data Availability Section)
**Cell lines:** Provide species information, strain. Provide accession number in repository **OR** supplier name, catalog number, clone number, and/**OR** RRID.	Yes	Materials and Methods, Figures, Data avilability Section
**Primary cultures:** Provide species, strain, sex of origin, genetic modification status.	Not Applicable	
Report if the cell lines were recently **authenticated** (e.g., by STR profiling) and tested for mycoplasma contamination.	Yes	Materials and Methods

Experimental animals	Information included in the manuscript?	In which section is the information available? (Reagents and Tools Table, Materials and Methods, Figures, Data Availability Section)
**Laboratory animals or Model organisms:** Provide species, strain, sex, age, genetic modification status. Provide accession number in repository **OR** supplier name, catalog number, clone number, **OR** RRID.	Yes	Materials and Methods, Figures, Data avilability Section
**Animal observed in or captured from the field:** Provide species, sex, and age where possible.	Not Applicable	
Please detail **housing and husbandry conditions**.	Yes	Materials and Methods

Plants and microbes	Information included in the manuscript?	In which section is the information available? (Reagents and Tools Table, Materials and Methods, Figures, Data Availability Section)
**Plants:** provide species and strain, ecotype and cultivar where relevant, unique accession number if available, and source (including location for collected wild specimens).	Not Applicable	
**Microbes:** provide species and strain, unique accession number if available, and source.	Not Applicable	

Human research participants	Information included in the manuscript?	In which section is the information available? (Reagents and Tools Table, Materials and Methods, Figures, Data Availability Section)
If collected and within the bounds of privacy constraints report on age, sex and gender or ethnicity for all study participants.	Not Applicable	

Core facilities	Information included in the manuscript?	In which section is the information available? (Reagents and Tools Table, Materials and Methods, Figures, Data Availability Section)
If your work benefited from core facilities, was their service mentioned in the acknowledgments section?	Yes	Materials and Methods, Acknowledgements

Design

Study protocol	Information included in the manuscript?	In which section is the information available? (Reagents and Tools Table, Materials and Methods, Figures, Data Availability Section)
If study protocol has been **pre-registered, provide DOI in the manuscript**. For clinical trials, provide the trial registration number **OR** cite DOI.	Not Applicable	
Report the **clinical trial registration number** (at ClinicalTrials.gov or equivalent), where applicable.	Not Applicable	

Laboratory protocol	Information included in the manuscript?	In which section is the information available? (Reagents and Tools Table, Materials and Methods, Figures, Data Availability Section)
Provide DOI OR other citation details if **external detailed step-by-step protocols** are available.	Not Applicable	

Experimental study design and statistics	Information included in the manuscript?	In which section is the information available? (Reagents and Tools Table, Materials and Methods, Figures, Data Availability Section)
Include a statement about **sample size** estimate even if no statistical methods were used.	Yes	Materials and Methods
Were any steps taken to minimize the effects of subjective bias when allocating animals/samples to treatment (e.g. **randomization procedure**)? If yes, have they been described?	Yes	Materials and Methods
Include a statement about **blinding** even if no blinding was done.	Not Applicable	
Describe **inclusion/exclusion criteria** if samples or animals were excluded from the analysis. Were the criteria pre-established?<br><br>If sample or data points were omitted from analysis, report if this was due to attrition or intentional exclusion and provide justification.	Not Applicable	
For every figure, are **statistical tests** justified as appropriate? Do the data meet the assumptions of the tests (e.g., normal distribution)? Describe any methods used to assess it. Is there an estimate of variation within each group of data? Is the variance similar between the groups that are being statistically compared?	Yes	Materials and Methods , Figure Legends

Sample definition and in-laboratory replication	Information included in the manuscript?	In which section is the information available? (Reagents and Tools Table, Materials and Methods, Figures, Data Availability Section)
In the figure legends: state number of times the experiment was **replicated** in laboratory.	Yes	Materials and Methods, Figure Legends
In the figure legends: define whether data describe **technical or biological replicates**.	Yes	Materials and Methods, Figure Legends

Ethics

Ethics	Information included in the manuscript?	In which section is the information available? (Reagents and Tools Table, Materials and Methods, Figures, Data Availability Section)
Studies involving **human participants**: State details of **authority granting ethics approval** (IRB or equivalent committee(s), provide reference number for approval.	Not Applicable	
Studies involving **human participants**: Include a statement confirming that **informed consent** was obtained from all subjects and that the experiments conformed to the principles set out in the WMA Declaration of Helsinki and the Department of Health and Human Services Belmont Report.	Not Applicable	
Studies involving **human participants:** For publication of **patient photos**, include a statement confirming that consent to publish was obtained.	Not Applicable	
Studies involving experimental **animals**: State details of **authority granting ethics approval** (IRB or equivalent committee(s), provide reference number for approval. Include a statement of compliance with ethical regulations.	Yes	Materials and Methods
Studies involving **specimen and field samples:** State if relevant **permits** obtained, provide details of authority approving study; if none were required, explain why.	Not Applicable	

Dual Use Research of Concern (DURC)	Information included in the manuscript?	In which section is the information available? (Reagents and Tools Table, Materials and Methods, Figures, Data Availability Section)
Could your study fall under dual use research restrictions? Please check biosecurity documents and list of **select agents and toxins** (CDC): https://www.selectagents.gov/sat/list.htm	Not Applicable	
If you used a select agent, is the security level of the lab appropriate and reported in the manuscript?	Not Applicable	
If a study is subject to dual use research of concern regulations, is the name of the **authority granting approval and reference number** for the regulatory approval provided in the manuscript?	Not Applicable	

Reporting

The MDAR framework recommends adoption of discipline-specific guidelines, established and endorsed through community initiatives. Journals have their own policy about requiring specific guidelines and recommendations to complement MDAR.

Adherence to community standards	Information included in the manuscript?	In which section is the information available? (Reagents and Tools Table, Materials and Methods, Figures, Data Availability Section)
State if relevant guidelines or checklists (e.g., **ICMJE, MIBBI, ARRIVE, PRISMA**) have been followed or provided.	Not Applicable	
For **tumor marker prognostic studies,** we recommend that you follow the **REMARK** reporting guidelines (see link list at top right). See author guidelines, under 'Reporting Guidelines'. Please confirm you have followed these guidelines.	Not Applicable	
For **phase II and III randomized controlled trials**, please refer to the **CONSORT** flow diagram (see link list at top right) and submit the CONSORT checklist (see link list at top right) with your submission. See author guidelines, under 'Reporting Guidelines'. Please confirm you have submitted this list.	Not Applicable	

Data Availability

Data availability	Information included in the manuscript?	In which section is the information available? (Reagents and Tools Table, Materials and Methods, Figures, Data Availability Section)
Have **primary datasets** been deposited according to the journal's guidelines (see 'Data Deposition' section) and the respective accession numbers provided in the Data Availability Section?	Yes	Data Availability Section
Were **human clinical and genomic datasets** deposited in a public access-controlled repository in accordance to ethical obligations to the patients and to the applicable consent agreement?	Not Applicable	
Are **computational models** that are central and integral to a study available without restrictions in a machine-readable form? Were the relevant accession numbers or links provided?	Yes	Data Availability Section
If publicly available data were reused, provide the respective **data citations in the reference list**.	Yes	Tables, References
